# Phylogenetically Driven Sequencing of Extremely Halophilic Archaea Reveals Strategies for Static and Dynamic Osmo-response

**DOI:** 10.1371/journal.pgen.1004784

**Published:** 2014-11-13

**Authors:** Erin A. Becker, Phillip M. Seitzer, Andrew Tritt, David Larsen, Megan Krusor, Andrew I. Yao, Dongying Wu, Dominique Madern, Jonathan A. Eisen, Aaron E. Darling, Marc T. Facciotti

**Affiliations:** 1 Microbiology Graduate Group, University of California, Davis, Davis, California, United States of America; 2 Genome Center, University of California, Davis, Davis, California, United States of America; 3 Department of Biomedical Engineering, University of California, Davis, Davis, California, United States of America; 4 Proteome Software, Portland, Oregon, United States of America; 5 Department of Energy Joint Genomes Institute, Walnut Creek, California, United States of America; 6 Department of Earth and Planetary Sciences, University of California, Davis, Davis, California, United States of America; 7 Université Grenoble Alpes, Institut de Biologie Structurale, Grenoble, France; 8 Centre National de la Recherche Scientifique, Institut de Biologie Structurale, Grenoble, France; 9 Commissariat à l'énergie Atomique et aux Énergies Alternatives, Département du Science du Vivant, Institut de Biologie Structurale, Grenoble, France; 10 ithree Institute, University of Technology, Sydney, Australia; University of Illinois at Urbana-Champaign, United States of America

## Abstract

Organisms across the tree of life use a variety of mechanisms to respond to stress-inducing fluctuations in osmotic conditions. Cellular response mechanisms and phenotypes associated with osmoadaptation also play important roles in bacterial virulence, human health, agricultural production and many other biological systems. To improve understanding of osmoadaptive strategies, we have generated 59 high-quality draft genomes for the haloarchaea (a euryarchaeal clade whose members thrive in hypersaline environments and routinely experience drastic changes in environmental salinity) and analyzed these new genomes in combination with those from 21 previously sequenced haloarchaeal isolates. We propose a generalized model for haloarchaeal management of cytoplasmic osmolarity in response to osmotic shifts, where potassium accumulation and sodium expulsion during osmotic upshock are accomplished via secondary transport using the proton gradient as an energy source, and potassium loss during downshock is via a combination of secondary transport and non-specific ion loss through mechanosensitive channels. We also propose new mechanisms for magnesium and chloride accumulation. We describe the expansion and differentiation of haloarchaeal general transcription factor families, including two novel expansions of the TATA-binding protein family, and discuss their potential for enabling rapid adaptation to environmental fluxes. We challenge a recent high-profile proposal regarding the evolutionary origins of the haloarchaea by showing that inclusion of additional genomes significantly reduces support for a proposed large-scale horizontal gene transfer into the ancestral haloarchaeon from the bacterial domain. The combination of broad (17 genera) and deep (≥5 species in four genera) sampling of a phenotypically unified clade has enabled us to uncover both highly conserved and specialized features of osmoadaptation. Finally, we demonstrate the broad utility of such datasets, for metagenomics, improvements to automated gene annotation and investigations of evolutionary processes.

## Author Summary

The ability to adjust to changing osmotic conditions (osmoadaptation) is crucial to the survival of organisms across the tree of life. However, significant gaps still exist in our understanding of this important phenomenon. To help fill some of these gaps, we have produced high-quality draft genomes for 59 osmoadaptation “experts” (extreme halophiles of the euryarchaeal family *Halobacteriaceae*). We describe the dispersal of osmoadaptive protein families across the haloarchaeal evolutionary tree. We use this data to suggest a generalized model for haloarchaeal ion transport in response to changing osmotic conditions, including proposed new mechanisms for magnesium and chloride accumulation. We describe the evolutionary expansion and differentiation of haloarchaeal general transcription factor families and discuss their potential for enabling rapid adaptation to environmental fluxes. Lastly, we challenge a recent high-profile proposal regarding the evolutionary origins of the haloarchaea by showing that inclusion of additional genomes significantly reduces support for a proposed large-scale horizontal gene transfer into the ancestral haloarchaeon from the bacterial domain. This result highlights the power of our dataset for making evolutionary inferences, a feature which will make it useful to the broader evolutionary community. We distribute our genomic dataset through a user-friendly graphical interface.

## Introduction

Organisms across the tree of life routinely experience changes in osmotic conditions. The ability to adjust physiological responses to these osmotic fluxes plays a role in processes ranging from desiccation tolerance and virulence of pathogenic bacteria [1], [2], to drought resistance in food crops [3], to mammalian reproduction [4]. In humans, osmotic response is essential for proper functioning of the heart [5], kidneys [6] and nervous system [7], and defects in osmo-response are implicated in a variety of chronic disorders [8]. Although there exists a large body of work on osmoadaptation, there remain a number of gaps in our knowledge. For example, how are different osmoadaptation strategies dispersed across phylogenetic space? Does there exist a strict delimitation between obligate halophiles and halotolerant organisms, or do these designations obscure a more nuanced biological reality? How do organisms with a wide range of salinity tolerances regulate the large physiological changes required to rapidly adapt to fluctuations in environmental salinity? Is there a fitness trade-off between static adaptation to constant level of high-salinity and the ability to adapt to changing salinity levels? How did the halophilic phenotype arise in evolutionary history? Here we use comparative genomics of a large number of extreme halophiles to begin to fill in some of the gaps in our current understanding of osmoadaptation.

The haloarchaea (a family of microorganisms belonging to the domain Archaea) have mastered the art of osmoadaptation. Members of this family thrive in extremely saline environments (up to NaCl saturation), and must constantly adapt to large shifts in salinity due to rainfall and evaporation. Although united by their ability to live in hypersaline environments (salinity greater than that of ocean water), the haloarchaea exhibit a diverse set of metabolic capabilities and span a broad range of environmental phenotypes [9], [10], including psychrotolerance (growth below 10°C), thermotolerance (growth above 45°C), and alkaliphilicity (maximum growth in basic environments). This diversity, along with the presence of well-developed genetic and biochemical toolkits [11], [12], makes this clade an excellent target of study for expanding our understanding of osmo-response.

To investigate the genetic potential for osmo-response in this clade, we have sequenced high-quality draft genomes for 59 species of haloarchaea isolated from 20 countries across six continents and environments ranging from fermented fish sauce to Permian age salt deposits (see Table S1). Combined with 21 previously sequenced haloarchaeal genomes, this dataset provides a rich opportunity for insight into osmoadaptation. Here we present an analysis highlighting adaptations to high salt at the gene and protein levels as well as analysis of ion transport capabilities and transcriptional machinery likely to play a role in mediating responses to changing osmotic conditions.

This genomic dataset will also be of use to the broader genomic and archaeal research communities. Although the archaea play major roles in global element cycling and ecosystem stability, this domain has been understudied. Our sequencing project nearly quintuples the number of available genomes for the haloarchaea and increases by ∼30% the number of sequenced archaea. We demonstrate several utilities of this dataset for defining community structure in metagenomic studies, improving automated genome annotation, and inferring the timing of evolutionary events along the haloarchaeal tree.

To facilitate large-scale use of this dataset, we have made sequence and annotation information available through an SQL database as well as the NCBI genome repository (for accession numbers, see Table S2). We provide gene calls and annotations derived using two independent automated annotation pipelines - the Rapid Annotation using Subsystems Technology (RAST) server [13] and NCBI's Prokaryotic Genome Annotation Pipeline (PGAAP) [14]. Using a combination of BLAST [15] and TRIBE-MCL [16], we have generated clusters of homologous proteins representing distinct protein families. We have made genome data and homology clusters for all 80 sequenced haloarchaea available through the genome context visualization tool JContextExplorer [17] (see Text S1 for access instructions). We believe that this genomic data will provide a rich source of information for the archaeal, genomics, evolutionary biology, and systems biology research communities for many years to come.

## Results/Discussion

### Sequencing, assembly and annotation

**Figure 1:**
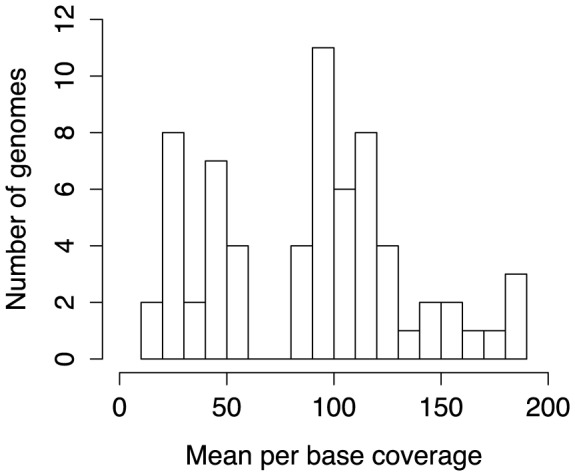
Sequencing depth of newly sequenced haloarchaeal genomes. Histogram showing distribution of mean per base coverage for the 59 newly-sequenced and seven previously published draft genomes [109] included in this study.

Fifty-nine haloarchaeal isolates from 17 genera were sequenced on Illumina GAII and HiSeq platforms using a combination of paired-end (85 nt reads), mate-pair (6 Kbp fragments), PCR-free paired-end, and unbarcoded “SOUP” libraries. SOUP libraries were prepared by pooling unbarcoded libraries for several species, which were phylogenetically distant enough to enable unambiguous read mapping. For library preparation details, see Materials and Methods; for information on methods used for each genome, see Table S2. Mean per base sequencing depth ranged from 13x to 188x, with a mean coverage of 90x (Figure 1). Sequence reads were assembled into contigs using the a5 pipeline [18], with a cross-species mean of 75 and median of 66 contigs. Genome assemblies ranged from 3.06 to 4.94 Mbp in size and all demonstrated high G+C content (mean of 62%) and high coding density (mean of 82%), as expected. Contig assemblies have been deposited in the NCBI genomic database along with annotations derived from the Prokaryotic Genome Annotation Pipeline (PGAAP) [14]. This annotation pipeline called between 2,945 and 4,645 putative protein coding regions, depending on the species.

A large fraction of proteins (∼41%) were annotated as hypothetical or of unknown function, likely a consequence of low experimental coverage of the archaeal domain. As sequencing projects study organisms of increasingly distant relationship to experimentally characterized model organisms, our ability to accurately analogize functions based on homology to previously characterized proteins declines. As such, these 124,149 unannotated haloarchaeal proteins represent a rich set of potential experimental targets for uncovering mechanisms of salt adaptation and other aspects of archaeal biology. Progress in understanding these mechanisms will benefit from an experimental focus on highly-conserved haloarchaeal proteins, as these are most likely to be involved in physiological processes integral to haloarchaeal biology. A significant fraction of these proteins are widely distributed, with 44% present in at least 10 of the 23 haloarchaeal genera with sequenced members, and 34% present in at least half of the included 80 genomes. To facilitate informed selection of targets for experimental work, we provide the distribution of these proteins in Dataset S1. Incorporation of our dataset into existing curated databases and automated workflows will facilitate downstream extrapolation of functional information learned from experimental approaches.

Since the beginning of our study, genome data from independently conducted sequencing projects have been released for several species included in the present study. For a comparison of sequencing statistics for these independently sequenced genomes see Table S3.

### An updated haloarchaeal phylogeny

**Figure 2:**
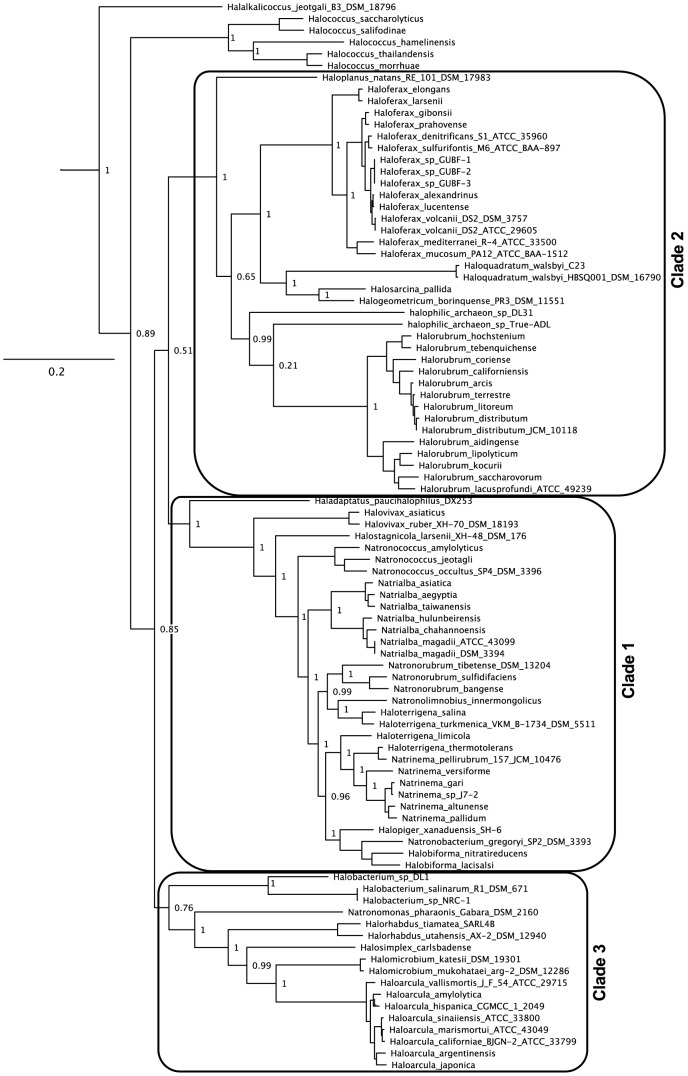
Updated haloarchaeal phylogeny. Multi-marker concatenated phylogeny of the 80 genomes included in this study and other haloarchaeal genomes gathered from IMG. The unrooted tree was built from a concatenated alignment of 40 PhyEco markers using PHYML. Branch support values for top-level branches have been removed for ease of visualization. The full tree file can be accessed through Dataset S19. Grey boxes represent haloarchaeal clades as described in [19], expanded as described in text. Tree roots in the Methanocella (not shown).

Previous phylogenetic studies have described two major haloarchaeal clades and several smaller groups with poorly defined relationships to these clades [19]. Here we update this previous work based on a phylogeny constructed using a concatenated set of 40 highly conserved genes (Figure 2) [20]. We expand the previously defined clades as follows: we consider a species to belong to a clade if a member of that genus was previously assigned to that clade and the genus is not paraphyletic or polyphyletic, and we also include any species which group with that clade with at least 75% support. Using this process for determining clade relationship, the increased resolution in the multi-marker phylogeny allows us to assign *Halovivax* to Clade 1. On the basis of the same phylogeny, we also propose designation of a third haloarchaeal clade, including the *Halobacterium*, *Natronomonas*, *Halorhabdus*, *Halosimplex*, *Halomicrobium*, and *Haloarcula* genera.

Previous studies have commented on the poorly resolved relationship between the *Haloterrigena* and *Natrinema* genera, which were originally designated based on lipid composition and DNA-DNA hybridization patterns [21]. Although Tindall [21] suggests that difficulties in genera-level assignment of some *Haloterrigena* and *Natrinema* species are simply the result of experimental error (including faulty DNA-DNA hybridization data), our results suggests that these genera, as currently defined, are actually polyphyletic. Species within these genera should therefore be reassigned using modern phylogenetic metrics. The multi-marker phylogeny was also instrumental in resolving other apparent genera-level paraphylies and polyphylies. These include the *Natronorubrum* and *Halobiforma* genera, which appear to be non-monophyletic when only *rpoB*' DNA or protein sequence similarity is considered [22].

### Breadth of the haloarchaeal pangenome

**Figure 3:**
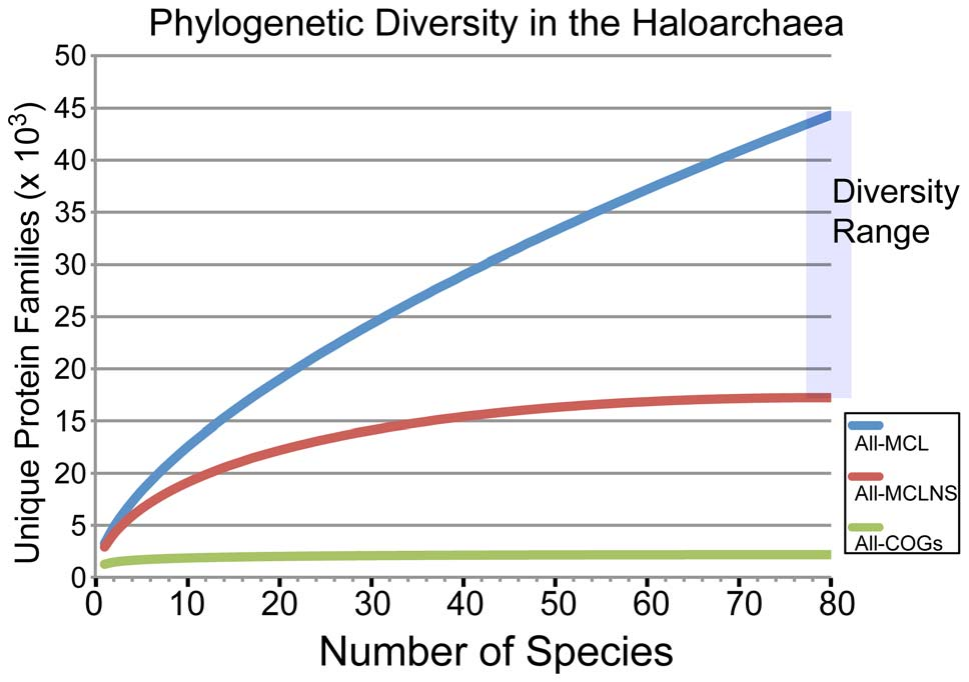
Rarefaction analysis of sampled haloarchaeal protein space. A rarefaction curve of protein diversity was created for the 80 haloarchaea included in this study using three alternative methods to define protein families: COG number (green), TRIBE-MCL clusters removing singletons (red), and TRIBE-MCL clusters without removing singletons (blue). The COG database is expected to represent an under-estimate of the true diversity. Similarly, using TRIBE-MCL clusters with all singleton genes excluded underestimates true diversity. The true diversity of the set is likely located between the blue and red curves.

To estimate the fraction of haloarchaeal phylogenetic diversity represented by this set of 80 haloarchaea, we performed rarefaction analysis, plotting the number of unique protein families against the number of randomly drawn genomes (Figure 3). Accurately grouping proteins into families is a non-trivial problem that has sparked the development of a large number of protein clustering algorithms [23]. As there is no experimental data for the vast majority of haloarchaeal proteins, clustering must rely on computational sequence similarity metrics. We therefore selected three methods to define protein families and generated rarefaction curves for each. The methods were as follows: (1) COG orthology groups [24], (2) in-house homology clusters defined using the clustering algorithm TRIBE-MCL [16] (see Materials and Methods, Dataset S2, Figures S1 & S2)), and (3) TRIBE-MCL defined homology clusters excluding those with only a single member (singletons).

As the COG database is limited to proteins with putative orthologs in at least three major phylogenetic groups, and only two archaeal lineages are included in the genomic dataset used to build the COGs (Euryarchaeota and Crenarchaeota), protein families unique to the archaea are not present in this database. Thus, only protein families that existed prior to the eukaryotic-archaeal split or were subsequently exchanged by horizontal gene transfer between eukaryotes or bacteria and archaea are included. As such, the rarefaction curve of COG proteins in the haloarchaea saturates very quickly, with only 2,172 COGs being present in the haloarchaea, and only 17 genomes being required to discover 90% of these families.

In contrast, using the TRIBE-MCL derived homology clusters, which do not exclude proteins specific to the archaea, the number of unique protein families in the sequenced set of haloarchaeal genomes was 17,223. Forty-two genomes were required to discover the first 90% of these families, when singleton genes were excluded. Including singletons, however, it is apparent that much more haloarchaeal diversity remains to be discovered, as more than 300 new protein families are added with each new genome. Taking into account the likelihood that many singleton gene families are the result of spurious gene calls, the true sampled phylogenetic diversity of the haloarchaea lies between the singleton-excluded and the singleton-included cases. By revealing the diversity of the haloarchaeal pangenome, this analysis highlights the importance of deep sequencing for phylogenetically informed selection of experimental targets.

### The haloarchaea did not originate via mass-acquisition of 1,000 bacterial genes by a methanogen

A recent paper analyzing 10 haloarchaeal genomes posited that the evolution of the haloarchaeal phenotype was the result of a single mass horizontal transfer of ∼1,000 bacterial genes into an ancestral archaeal methanogen [25]. This conclusion was based on 1) the finding that a large number of gene trees (1,089), built from a set of protein families with both archaeal and bacterial members (1,479), placed haloarchaeal genes in a monophyletic group with bacterial rather than archaeal homologs and 2) diverse phylogenetic evidence supporting the Methanomicrobia as sister group to the Haloarchaea [26]–[28] (see also Dataset S3). Further supporting this hypothesis, several of these transferred genes were associated with functions required for the proposed physiological transformation of an obligately anaerobic, autotrophic methanogen to a heterotrophic, facultatively aerobic haloarchaeon [25].

Accuracy of inferred evolutionary events is strongly influenced by the selection of representative species on which the inference is based. We tested whether the conclusions made by Nelson-Sathi, et al. [25] from an analysis of 10 haloarchaeal genomes were robust to a reanalysis against our more phylogenetically diverse dataset. In our analysis we also observed a large amount of horizontal gene transfer from the bacteria, however, we found that this exchange of genetic material was not limited to a single acquisition event at the haloarchaeal root, but rather occurred in many different transfer events throughout haloarchaeal evolution.

For each of the protein families in the original set reported by Nelson-Sathi, et al. [25], we added homologs from 65 additional haloarchaeal genomes and rebuilt gene trees using the same software tools and parameters (obtained via correspondence with the authors). More than two-thirds (67.2%) of the protein families originally designated as basal acquisitions no longer retained this characteristic after incorporating the additional haloarchaeal homologs (Table 1). Analysis of the re-computed gene trees revealed that, not only did most transfers not happen near the base of the haloarchaeal clade, but, for many protein families, multiple independent transfer events from bacteria to the haloarchaea have occurred. Depending on the gene, these additional transfers either predate (Figure S3) or follow (Figure S4) the acquisition discovered by Nelson-Sathi, et al. Both cases are inconsistent with a single, basal transfer scenario: rather, our results are consistent with previous findings that horizontal gene transfer is rampant among bacteria and archaea [29]. This interpretation is further supported by the fact that the putatively transferred genes do not appear to have been transferred from a common bacterial phylum, as indicated by the phylogenetic affiliation of the most closely related bacterial homolog for each protein family (see Table S2 in [25]).

**Table 1:** Number of inferred basal bacterial imports decreases with added genomes.

Dataset	Trees assessed	Basal imports	Non-monophylies
Original	1479	1089 (73.6%)	390 (26.4%)
Re-analysis	1479	656 (44.4%)	823 (55.6%)
Haloarchaeal-extended	543	178 (32.8%)	365 (67.2%)

The simplest explanation for the difference in findings between the original study and our reanalysis is rooted in the nature of the two genomic datasets investigated. Due to the limited number of genomes available at the time, Nelson-Sathi, et al. worked under the assumption that the 10 haloarchaeal genomes they sampled reasonably represented haloarchaeal diversity. This lead to the identification of genes as bacterial transfers to the last common ancestor of the haloarchaea on the basis of as few as two haloarchaeal homologs. Based on the distribution of genomes used (two from Clade 1, two from Clade 2, and six from Clade 3, see Figure 2), a gene present in only two haloarchaea will often represent a transfer to a single clade, rather than to the haloarchaeal root. By contrast, our genomic dataset, representing a more even phylogenetic sampling of the haloarchaea, revealed multiple, clade specific transfer events indicative of a complicated history of gene transfer between the haloarchaea and bacteria. This analysis highlights the value of large, phylogenetically informed genomic data sets for increasing the accuracy with which we can make evolutionary inferences, and reveals the dangers in making wide-reaching evolutionary claims based on limited genomic data.

### Insights into salt adaptation from the haloarchaeal core genome

Despite their metabolic and physiological diversity [9], [10], all members of the haloarchaeal clade share an obligately halophilic lifestyle. To understand the common mechanisms underlying this lifestyle, we investigated the haloarchaeal core genome. A total of 304 of the in-house defined protein families (see Materials and Methods) were present in all of the 80 investigated genomes (Table S4). Of these 304 core proteins, 55 (18.1%) were predicted to be involved in translation, transcription, or regulation thereof. In addition to the expected ribosomal proteins, RNA polymerase subunits, and known general transcription factors, the core genome included a number of predicted transcription factors whose functional importance in regulating haloarchaeal gene expression is underexplored. These include a ArsR-family transcription factor involved in alleviation of heavy metal toxicity, an AsnC-family member involved in feast/famine response, a CBS domain-containing protein of unknown function, and a PadR-family protein, possibly involved in regulation of phenolic acid metabolism [30]. As the level of functional specificity provided by domain-level matches is limited (for example, PadR-family proteins have also been shown to be involved in regulation of multidrug pumps [31]) the contributions of these conserved transcriptional regulators to haloarchaeal biology will need to be experimentally determined. However, their wide distribution across 23 haloarchaeal genera suggests that these proteins likely play important roles in regulating physiological responses to shifting environmental parameters routinely experienced in hypersaline environments, including changes in oxygen availability, salinity, and concentrations of heavy metals.

The core genome includes 10 protein families predicted to be involved in stress response, including both cold shock and heat shock members as well as stress response proteins with no predicted specific function. Previous studies of haloarchaea have shown upregulation of heat and cold shock genes in response to salinity changes, indicating that these chaperones may play a wider role in mediating stress responses than previously believed [32], [33]. As such, these 10 core haloarchaeal stress response proteins are candidates for experimental work to study the complex interplay among different stress response mechanisms.

DNA mismatch (MutSL), homologous recombination (RadAB) and base excision repair mechanisms are also universally conserved in this clade. Conspicuously lacking from the haloarchaeal core genome is a photolyase, responsible for correcting UV induced thymidine-dimers. Seven species were missing an annotated photolyase, all of which were Clade 1 haloarchaea (see Text S1 and Figure 2). If these genes are indeed absent – not hiding in unassembled regions of the genome - their absence would be surprising, given that many haloarchaeal species are routinely exposed to high levels of UV radiation due to evaporation of shallow hypersaline lagoons. Only two of these species (*Natrialba taiwanensis* and *Natrialba aegyptia*) encode genes annotated as belonging to the UVR system of UV damage repair, with each encoding only one of the five proteins in this repair system. Although haloarchaeal high G+C content has been proposed as an adaptation for avoiding UV-induced dimerization [34], potentially reducing the need for a photolyase repair system, the link between G+C content and UV damage in this clade is far from certain and alternate explanations for high G+C content have been proposed [35].

A number of transport-related protein families were also universally conserved, including several ABC transporters with peptides, amino acids and/or metals as predicted substrates. ABC transporters were extremely abundant, making up six of only eleven protein families with greater than 400 members. Although precautions were taken to filter out spurious domain-level matches (see Materials and Methods), each of these ABC transporter families may be composed of many members with divergent substrate specificities. Four protein families related to phosphate transport were also conserved, including two low-affinity phosphate transporters and two regulators of phosphate transport.

Not surprisingly, a number of proteins involved in biosynthesis of isoprenoid lipids were conserved across the haloarchaea. Isoprenoids are characteristic of haloarchaeal cell membranes [36], and are known to reduce membrane permeability to Na^+^ and Cl^−^ ions [37], a necessary prerequisite for regulating ionic composition at high salinities. The committed step in isoprenoid synthesis may be upregulated under high salt conditions [38], further suggesting their importance to cell maintenance in hypersaline environments. However, as isoprenoids serve as precursors for a number of other compounds, alternative hypotheses must also be considered.

Fifty-five of the 304 conserved haloarchaeal proteins (∼20%) had either no assigned functional annotation or only a domain-level match to a previously characterized protein. Based on their high conservation, these proteins likely play important roles in the haloarchaeal biology, including adaptation to hypersaline environments. These 55 protein families, therefore, represent a manageable set of targets for exploring the genetic mechanisms of halophilicity.

### A generalized model for haloarchaeal osmoadaptation and ion transport

Haloarchaea are generally considered “salt-in” strategists – actively accumulating potassium and chloride ions to prevent water efflux in hypersaline environments. In contrast, the “salt-out” strategy entails accumulation or synthesis of organic compatible solutes to increase internal osmolarity without increasing cytoplasmic salinity. Although recent work has shown that some haloarchaea may utilize compatible solutes in some situations [39]–[41], and many halotolerant organisms transiently accumulate moderate levels of intracellular K^+^ ions in the initial stage of osmoadaptation [42], [43], distinguishing between salt-in and salt-out strategists remains useful for differentiating between obligate and facultative halophiles.

**Figure 4:**
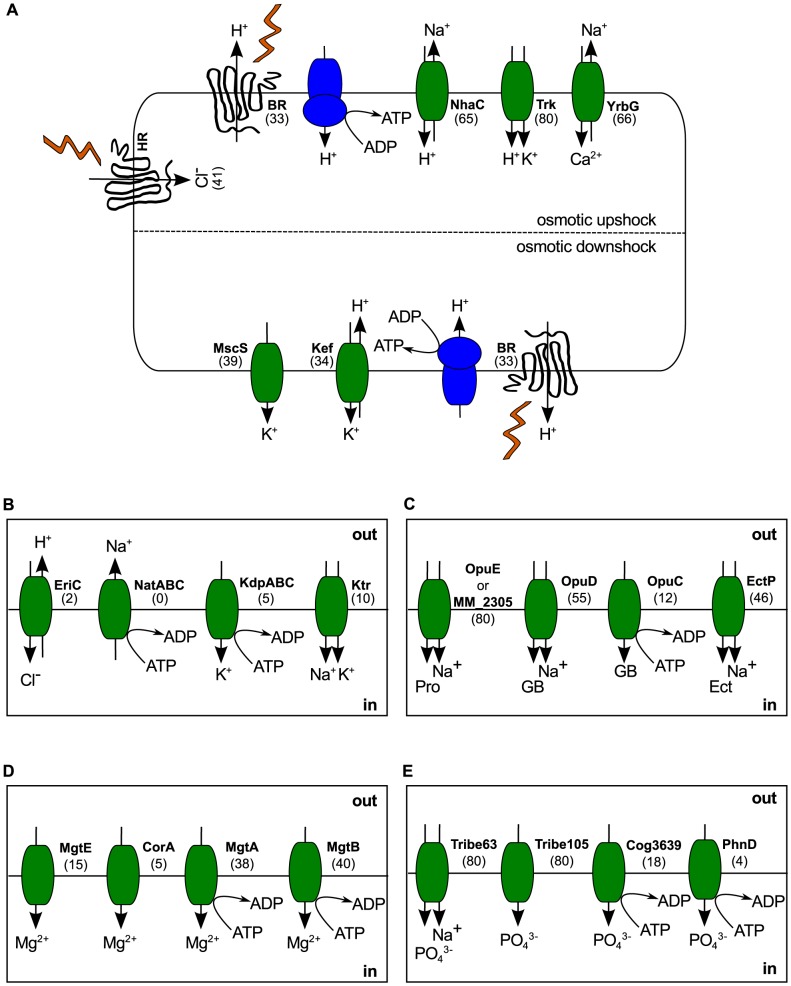
Haloarchaeal osmoadaptation. (A) Generalized model for osmoadaptation in the haloarchaea. During osmotic upshock, potassium is imported through Trk H^+^/K^+^ symporters and Na^+^ is expelled using a combination of NhaC H^+^/Na^+^ antiporters and/or YrbG Ca^2+^/Na^+^ antiporters. During osmotic downshock, excess potassium is removed through a combination of Kef H^+^/K^+^ antiporters and the non-specific mechanosensitive channel MscS. (B) Ion transport strategies not generally encoded by the haloarchaea. Sodium export and potassium import using ABC transporters (NatABC and KdpABC) may be less energetically efficient than secondary transport systems. Use of Ktr K^+^/Na^+^ symporters for potassium uptake would result in over-accumulation of sodium. (C) Compatible solute transport systems. Na+/proline symporters (OpuE and MM_2305) are ubiquitous in the haloarchaea. Glycine betaine uptake is mediated by OpuD through symport with sodium or, rarely, through the ABC transporter OpuC. The ectoine/sodium symporter EctP is also widespread. For transport systems with multiple substrates, a representative compound is shown. (D) Magnesium uptake is mediated by primary active transport (MgtA/B) or, rarely, by facilitated diffusion (MgtE/CorA). (E) Potassium accumulation is possible in all 80 haloarchaea via both secondary active transport (Tribe63) and facilitated diffusion (Tribe105). Some species also possess ATP-dependent potassium transporters (Cog3639/PhnD). Numbers in parenthesis represent the number of haloarchaeal species possessing the transporter gene. BR – bacteriorhodopsin, HR – halorhodopsin, GB – glycine betaine, Pro - proline, Ect – ectoine. Kef, Ktr and Trk each represent a class of transporters, rather than a single homolog. Tribe63 and Tribe105 refer to protein families defined in this study.

Due to the dynamic nature of hypersaline environments, the haloarchaea possess a range of ion transporters for accommodating fluctuating salinity levels. We investigated the phylogenetic distribution of a number of ion transporter genes potentially involved in osmoadaptation to hyper-osmotic or hypo-osmotic shock, as well as compatible solute import and biosynthesis genes (Figure S5). This analysis enabled us to propose a generalized haloarchaeal strategy for dynamic osmoadaptation (Figure 4A).

During osmotic upshock, potassium import and sodium extrusion are mediated by secondary transport, using the proton gradient generated either by direct light-activated proton translocation through bacteriorhodopsin (BR) or through respiration. All 80 haloarchaea investigated possess a H^+^/K^+^ symporter of the Trk family for potassium uptake, while few (10 species) also possess the closely related Na^+^/K^+^ symporter of the Ktr family (Figure 4B). This observation is consistent with Corratgé-Faillie et al.'s. prediction that use of the Trk system may be evolutionarily advantageous in hypersaline environments by avoiding Na^+^ uptake [44]. Many haloarchaea (66 species) may power sodium extrusion through YrbG Na^+^/Ca^2+^ antiporters, although this strategy would be limited to environments with high calcium concentration. During osmotic downshock, it is vital for organisms using a salt-in strategy to rapidly rid the cytoplasm of excess salts to avoid hypertonic cell lysis. The haloarchaea have the genetic potential to export excess potassium ions through a combination of secondary transport with Kef-like H^+^/K^+^ antiporters and non-specific ion loss through the mechanosensitive channel MscS, which has been shown to play an important role in potassium efflux during osmotic downshock in *E. coli* [45].

In contrast to the prevalence of these predicted secondary transporters, only a small number of genomes encode ATP-dependent transporters for osmoadaptation (Figure 4B). Prominently lacking in most haloarchaeal genomes are the outwardly rectifying Na^+^ pump NatABC and the inwardly rectifying K^+^ pump KdpABC, both of which depend on ATP hydrolysis to power ion transport. Previous researchers have calculated a large difference in energetic cost between salt-in and salt-out strategies, with production of compatible solutes being more costly than establishment of ionic gradients [46]. We propose that, in situations where large amounts of material must be transported, even a small difference in energetic efficiency between secondary and primary transport would result in a bias towards secondary transport systems, which require fewer steps. If true, this would explain the observed bias of haloarchaeal genomes for secondary transport systems for K^+^ accumulation and Na^+^ extrusion. However, experimental work will be required to calculate the ion exchange stoichiometry of these transporters and to determine the relative efficiency of secondary versus primary transport in this system.

Haloarchaeal strategies for uptake of chloride are difficult to decipher from the genomic data, as metabolism of this important counterion is not well understood [47]. The most recent review of the topic states that chloride is imported through “cotransport with sodium ions and/or using the light-driven primary chloride pump halorhodopsin” [47]. However, although experimental work has suggested the presence of a light-independent chloride uptake system in *Halobacterium sp.* NRC-1 [48], neither the energy source for this system, nor a genetic mechanism for its implementation have been identified. As only 41 of the organisms analyzed here possess a halorhodopsin homolog, some alternate strategy for chloride import must exist. We screened for homologs to archaeal and bacterial chloride transport proteins, including a predicted (Na^+^/K^+^)/Cl^−^ symporter from *Methanosarcina acetivorans* [49], a predicted bacterial cation chloride transporter from *Aminomonas paucivorans*, and EriC, a H^+^/Cl^−^ antiporter [50] involved in acidic shock tolerance in *Escherichia coli* [51]. Of these, we discovered only two EriC homologs, belonging to the alkalitolerant *Natrialba aegyptia* [52] and the alkaliphilic *Natrialba magadii* [53]. Based on these observations we propose a possible role for EriC homologs in Cl^−^ uptake in alkaliphilic environments where export of protons down their concentration gradient would enable accumulation of a high intracellular level of chloride ions.

Due to recent interest in haloarchaeal use of compatible solutes (as part of a broader osmoadaptation strategy which also includes potassium accumulation) [39]–[41], we analyzed the phylogenetic distribution of many genes involved in compatible solute transport and biosynthesis (Dataset S4). This analysis reveals prevalent uptake mechanisms for the compatible solutes glycine betaine, ectoine, and proline (Figure 4C), commonly used as osmoprotectants by facultative halophiles. Chemotaxis towards, and accumulation of the trimethylammonium compounds glycine betaine, carnitine, and choline, has been demonstrated in a model haloarchaeaon [39]. We find that, although putative homologs to the binding and transducer proteins for this system (CosB and CosT) are widely distributed, only 10 species possess both members (Figure S5). Due to high levels of sequence similarity between CosB and the trimethylammonium compound transporters OpuCC/OpuBC [39], we cannot confidently assert presence of a trimethylammonium compatible solute chemotaxis system in these species, and caution that experimental work must be done to validate these results. The abundance of compatible solute transporters within haloarchaeal genomes does not, however, necessarily indicate utilization of a salt-out strategy. During periods of decreased environmental salinity haloarchaea often coexist with halotolerant microorganisms, which are generally salt-out strategists. As evaporation increases salinity, these organisms lyse and the released compatible solutes may then be used as carbon and nitrogen sources by extreme halophiles [54]. Compatible solutes have also been shown to have thermoprotective effects in both mesophilic bacteria [55] and hyperthermophilic archaea [56]. Experimental evidence of compatible solute accumulation suggests that some haloarchaea may utilize a salt-out strategy under certain conditions [39]–[41].

Recently, biosynthesis of the compatible solute trehalose was found to be widespread in the haloarchaea [41]. We interrogated our genomes for genes associated with compatible solute biosynthesis, including the ProJH pathway for proline synthesis during osmotic upshock [57], the N^e^-acetyl-β-lysine and cyclic 2,3-bisphosphoglycerate (cBPG) synthesis pathways [43], [58], and the BetAB/GbsBA pathways for oxidation of choline to glycine betaine [59], [60] (Figure S5). We found that, in contrast to the widespread mechanisms for compatible solute uptake, pathways for biosynthesis of these compatible solutes were rare. Complete pathways for osmotically regulated proline synthesis and N^e^-acetyl-β-lysine production were absent in all 80 genomes. Although putative homologs to ProH were present in six species of *Halorubrum*, their function is unclear in the absence of ProJ, which catalyzes initial transformation of glutamate for proline biosynthesis [57]. One species (*Natronorubrum tibetense*) was found to encode an intact pathway for biosynthesis of cBPG, previously thought to be restricted to methanogens [58]. Only nine species were found to possess homologs for the three components required for glycine betaine synthesis from extracellular choline: 1) a choline transporter, 2) a choline dehydrogenase, and 3) a glycine betaine aldehyde dehydrogenase. In eight of these species, the preferred strategy appears to be choline uptake via OpuB, oxidation to glycine betaine aldehyde using GbsB, and final oxidation to glycine betaine via GbsA/BetB. The ninth species (*Halococcus saccharolyticus*) appears to utilize the flavin adenine dinucleotide-bound choline dehydrogenase BetA rather than the type III alcohol dehydrogenase GbsB [58] for initial choline oxidation.

We also investigated uptake mechanisms for the biologically important magnesium and phosphate ions (Figure 4D & E). For phosphate accumulation, all 80 sequenced haloarchaea encode two members of the PHO4 superfamily (PF01384), annotated as an anion permease (TRIBE-MCL cluster 105; Tribe105), and a sodium dependent phosphate transporter (Tribe63). This second annotation potentially enables active phosphate uptake at the expense of the sodium gradient in hypersaline environments. As discussed above in the context of potassium and sodium transport, ATP-dependent phosphate uptake appears not to be favored, with only 22 homologs of ABC-type phosphate transporters encoded in this genome set. The opposite was found to be the case with magnesium transport, with the ATP-dependent transporters MgtA and MgtB being far more common than the inwardly rectifying Mg^2+^ channels MgtE and CorA. However, with only 52 of 80 sequenced haloarchaea encoding at least one of these Mg^2+^ transport mechanisms, it is clear that alternate strategies for magnesium uptake remain to be discovered. As magnesium concentrations have been shown to play important roles in stabilizing halophilic enzymes [61], discovery of these alternative magnesium uptake strategies is vital to understanding the nature of halophilic proteins.

Our investigations into haloarchaeal ion transport reveal both a core set of highly conserved strategies (eg. Trk-based K^+^ uptake, Na^+^-mediated phosphate accumulation) as well as more sparsely distributed abilities (eg. alkaliphilic chloride import via EriC). We have observed that secondary transport seems to be preferred to ATP-dependent primary transport for maintenance of large ion gradients in hypersaline environments, and have identified important gaps in our understanding of chloride and magnesium accumulation. The generalized nature of our model of dynamic osmoadaptation in haloarchaea, based on a broad and deep sampling scheme, contrasts with the specificity of previous models, which were largely limited to single model systems widely spaced across the phylogenetic tree. By highlighting strategies conserved across the haloarchaea, we hope to help build a general understanding of osmoadaptation across the tree of life.

### Proteome acidification and exceptions to the rule

**Figure 5:**
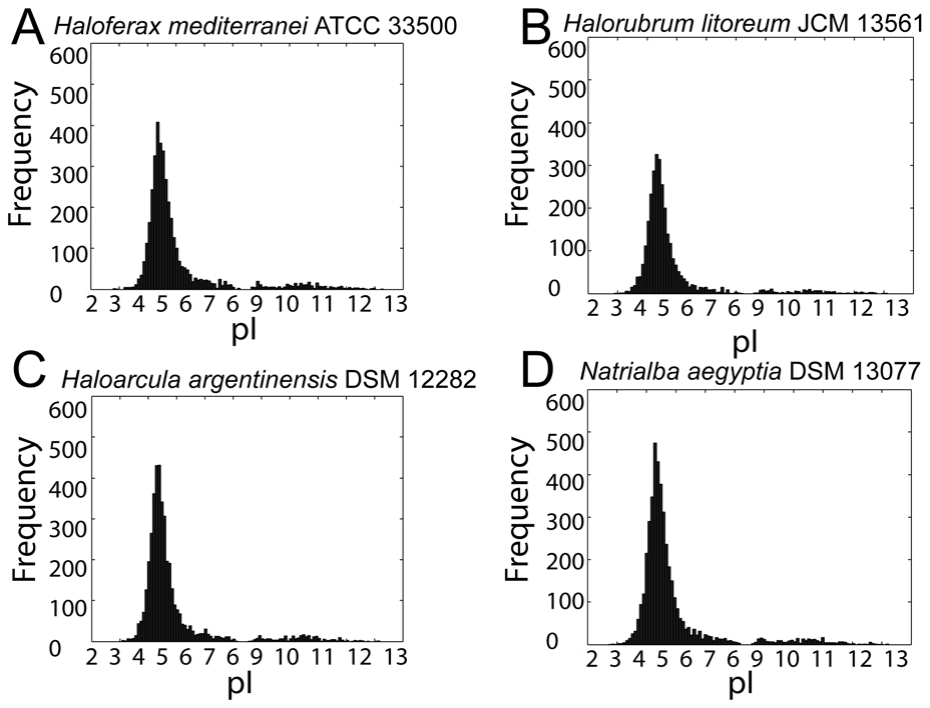
Proteome-wide isoelectric point distributions. Histograms of computed pI values for (A) *Haloferax mediterranei*, (B) *Halorubrum litoreum*, (C) *Haloarcula argentinensis*, and (D) *Natrialba aegyptia*. Each of these four species is a representative from the four most populous genera in the set, which collectively contain 66% of the organisms in the study. For all sequenced haloarchaea, the pI histograms exhibited a bimodal distribution with a major mode at about 4.5 and much smaller minor mode around 10. For all isoelectric point plots see Data Dryad package [67].

In addition to the ability to rapidly adapt to changing environmental salinities, haloarchaeal adaptations to high salt also include intrinsic physiological features present under all environmental conditions. These adaptations include an acidified proteome and high genomic G+C content [35], [62], [63], discussed here and in the subsequent section, respectively. Proteome acidification may be beneficial for salt-in strategists by prevention of protein aggregation via the ability of acidic amino acid residues to reorganize protein-solvent interactions [61], [64], [65], although alternative explanations have been proposed [66]. As expected, all 59 organisms whose draft genome we report here have both high genomic G+C content (ranging from 59–69%) and a highly acidified proteome. Histograms of predicted isoelectric points of haloarchaeal proteomes revealed an asymmetric bimodal distribution, with a dramatically larger major mode around pH 4.5, a minor mode around pH 10.0, and a consistent overall shape across haloarchaeal species (Figure 5 and Data Dryad package [67]). Corroborating previous work, this major mode was shifted towards lower pI values compared to non-haloarchaea [68].

**Figure 6:**
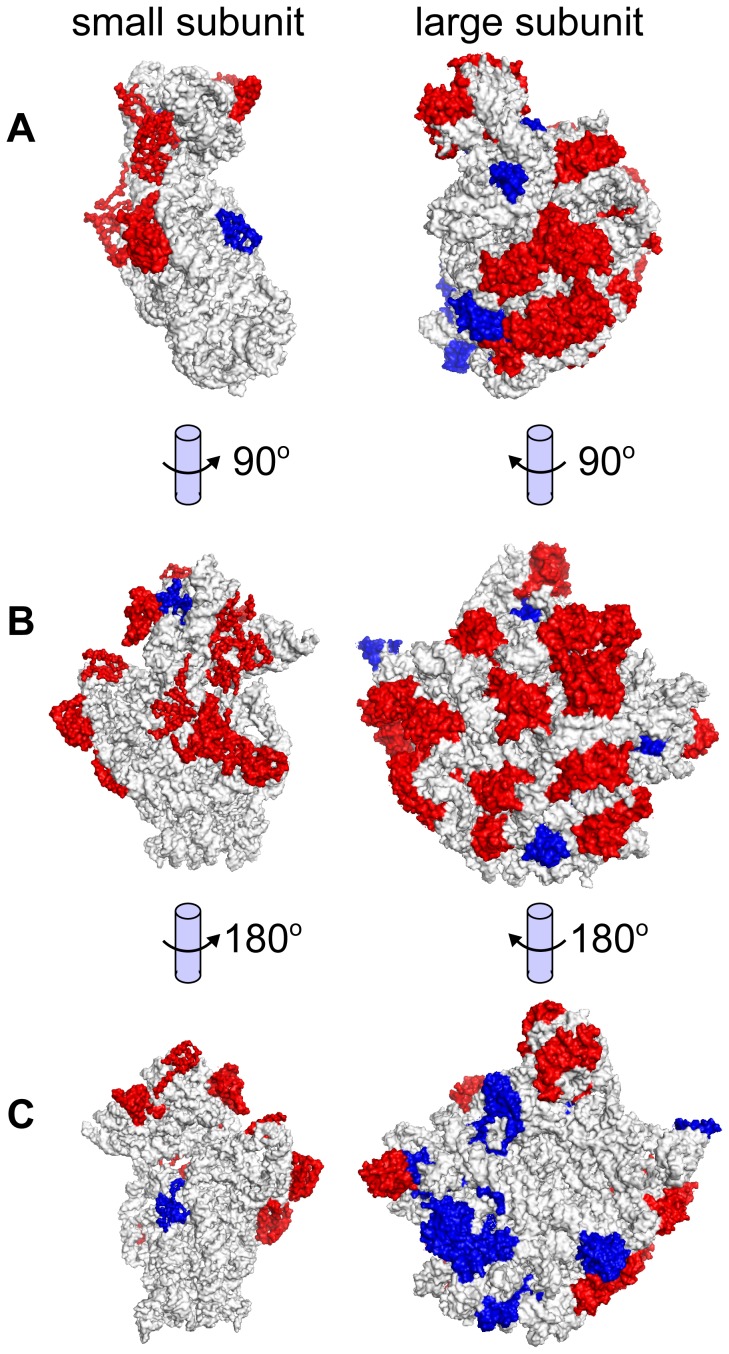
Ribosomal subunit isoelectric points. Structural mappings of the large and small ribosomal subunits, showing protein monomers with predicted low (red) and high (blue) pI. Subunits are oriented according to (A) complex formation, (B) cytoplasmic view of each subunit, and (C) internal view of each subunit. Subunits exposed to the cytoplasm tend to be acidified (red subunits, visible in B), while subunits buried within the ribosome tend to have an alkaline isoelectric point (blue subunits, visible in C). Structural models used were those for *Thermus thermophilus* (1FKA) [97] and *Haloarcula marismortui* (1QVG) [96].

Certain haloarchaeal proteins were not acidified, including many ribosomal subunits, membrane proteins, and DNA-binding proteins (Table S5). Potential reasons for non-acidification include 1) shielding from the hypersaline cytoplasm (eg. internal ribosome subunits, membrane proteins), and 2) presence of a selective force against acidification (eg. DNA-binding proteins). Structural mapping of the ribosome demonstrates that subunits exposed to the hypersaline cytosol tend to be acidified, while shielded internal subunits have high pI (Figure 6). Large regions of transporters and other membrane–associated proteins are likewise shielded from the saline cytoplasm by the cell membrane, mitigating selective pressure to acidify. DNA-binding proteins must retain positively charged residues to interact efficiently with negatively charged DNA, and so also tend not to acidify (shown for the general transcription factor TATA-binding protein (TBP), Figure S6, and ribosome elongation factor α-1, Figure S7). A large number (11,087) of non-acidified proteins were unannotated. Based on functional consistency we identified among other high pI proteins, we propose these proteins as candidates for exploratory research seeking novel DNA-binding or membrane associated proteins such as transcription factors, transporters, and chemotaxis/sensory receptors.

### Local variation in genomic G+C as a proxy for horizontal gene transfer events

In addition to their acidified proteomes, the highly G+C biased genomes of the haloarchaea are also predicted to be an adaptation for life in high salt. Although the mechanism for this adaptive benefit is unknown, several possibilities have been proposed, including decreased risk of thymine dimers resulting from high UV exposure in shallow brine pools [34], or selective pressure driven by A+T bias of insertion sequence elements [35]. Notably, the only known haloarchaeon lacking a G+C biased genome – *Haloquadratum walsbyi* (48%) – possesses a large number of photolyase genes, postulated to enable it to mitigate the effects of UV induced pyrimidine dimerization [69]. The Nanohaloarchaea, an uncultured clade of halophilic archaea proposed as a sister group to the Haloarchaea, also have low G+C content (43 and 56% for the two members of this clade with draft genomes), although they inhabit the same hypersaline environments as the high G+C Haloarchaea [70]. The evolutionary rationale behind this difference is unknown.

**Figure 7:**
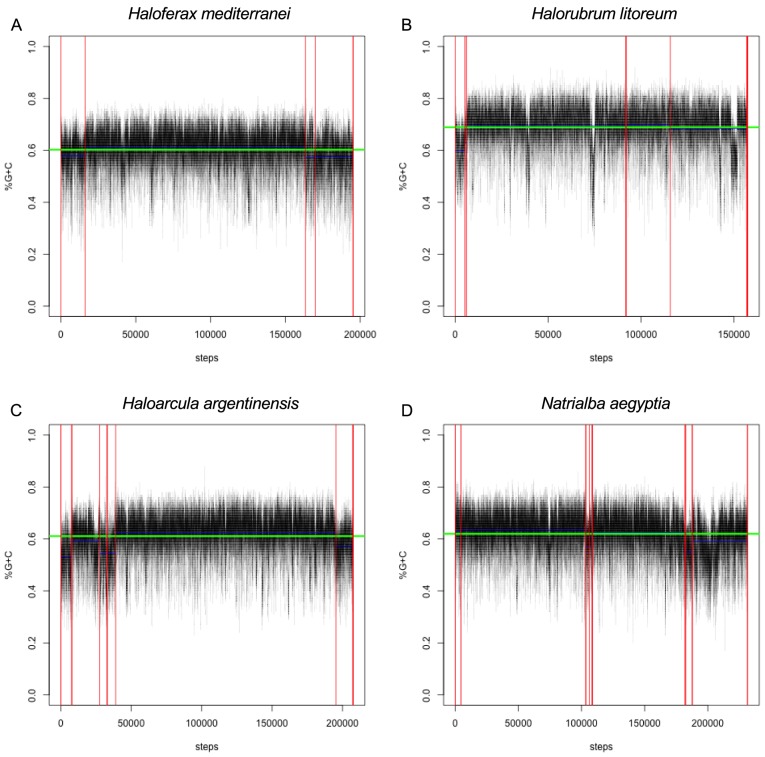
Sliding-window G+C content analysis. Representative G+C content plots for (A) *Haloferax mediterranei*, (B) *Halorubrum litoreum*, (C) *Haloarcula argentinensis*, and (D) *Natrialba aegyptia*. Each of these four species is a representative from the four most populous genera in the set, which collectively contain 66% of the organisms in the study. Black line represents calculated G+C percent for each 100 bp window. Contig boundaries are represented as vertical red lines, contig mean G+C as horizontal blue lines, and genome mean G+C as a horizontal green line. The horizontal axis displays the number of 20 bp steps taken along the genome. For all G+C plots see Data Dryad package [67].

Regardless of the mechanism for its maintenance, genome-wide G+C bias offers a method for identification of candidates for horizontal gene transfer from organisms with G+C content differing from the recipient species, as horizontally transferred genes are often A+T shifted relative to the host genome [71]. We examined the G+C content of the 80 haloarchaeal genomes, using a sliding 100 bp window, and conducted change-point analysis to extract regions with local G+C content differing from the genome average. It is important to note here that haloarchaeal plasmids, including minichromosomes and megaplasmids, are known to have decreased G+C content compared with primary replicons (“chromosomes”) [63]. As the mechanisms for maintaining decreased G+C content in smaller replicons are unknown, and in order to accommodate draft genomes where the identity of the primary replicons are unknown, we have chosen to be replicon size neutral. Some regions of the genome are also expected to have low local G+C content due to selective pressure for maintaining a higher A+T percentage (eg. origin of replication sites). In addition, we have been neutral as to the direction of divergence from genome-wide G+C average, in order to allow detection of regions of unusually high as well as unusually low G+C (Figure 7, Figure S8, Datasets S6 & S7).

We found these regions to be highly enriched in protein families involved in DNA metabolism and transcriptional regulation, transmembrane transport (and other membrane proteins), and horizontal transfer of genetic information. Specifically, of the seventy-nine functionally annotated protein families with at least five members which were enriched at least eight-fold in the divergent G+C regions, thirty-six (45%) were annotated with DNA/RNA-binding capabilities, eleven (14%) were associated with horizontal gene transfer mechanisms, and seven (9%) were associated with the cell membrane or cell surface (Table S6). These results are consistent with our investigations into non-acidified haloarchaeal proteins, in that both analyses identified nucleic acid binding and transmembrane proteins as potentially shielded from selective pressure to acidify and accumulate high G+C content. However, the specific proteins identified by these analyses were not identical and previous work has indicated that G+C bias and acidification are not correlated for individual proteins [35]. We speculate that many of the 138 unannotated protein families enriched in these regions of abnormal G+C content may be involved in DNA or RNA binding. We provide these regions in our Data Dryad package [67], as a rich source of data for identification of novel nucleic acid binding proteins and investigation of functionally important horizontal gene transfer events into the haloarchaea.

**Figure 8:**
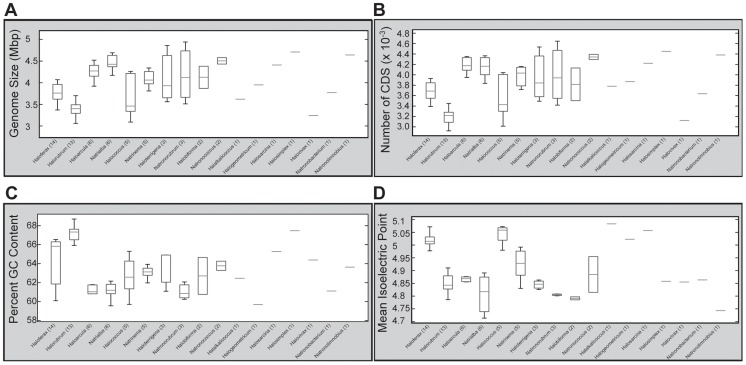
Genera-level comparisons of genomic features. Assembled genome size (Mbp) (A), number of predicted protein coding sequences (B), %G+C (C), and mean protein isoelectric point (D) were extracted from each genome, organized by genus, and boxplots calculated using MATLAB's Statistics toolbox. Boxplots were generated using 25^th^ and 75^th^ percentile as box edges, with median demarcated with horizontal line within box. Genera are ordered by descending number of species sequenced, with the number of species shown in parentheses. Genera with only a single sequenced member are shown as horizontal lines.

In addition to local variation in G+C content, we also investigated variation at the genus level. We found that, although some genera display little variability in genomic G+C content (eg. *Halorubrum*, *Haloarcula*), others exhibit a wide range (eg. *Haloferax*, *Halococcus*) (Figure 8C). This wide deviation in G+C content cannot be attributed to tolerance of a wide range of salinities, as the known NaCl tolerance range of *Haloarcula* and *Halococcus* species are very similar (3.2 M and 3.5 M respectively), as are those for *Haloferax* and *Halorubrum* species (4.1 M and 4.2 M respectively) [72]. Thus, the link between high G+C content and salinity tolerance in the haloarchaea appears to be more complex than previously appreciated.

### Multiple general transcription factors provide potential for rapid adaptation to environmental fluxes

Recent work has uncovered surprising roles for eukaryotic and archaeal general transcription factors in mediating differential gene regulation during cellular differentiation and environmental response [73]–[75]. In the haloarchaea, both the TATA-binding protein (TBP) and transcription factor B (TFB, known as transcription factor IIB in eukaryotes) families have undergone extensive expansion [75], [76]. TFB paralogs of *Halobacterium sp.* NRC-1 have been shown to differentially contribute to fitness under stresses commonly encountered in hypersaline environments, including variations in salinity and heavy metal concentration [75]. Multiple TBP and TFB paralogs may enable haloarchaeal species to quickly and efficiently modify transcriptional response to these environmental fluxes.

**Figure 9:**
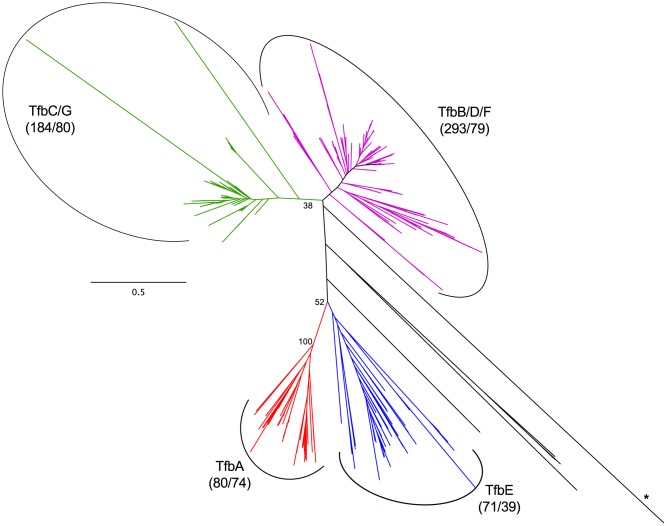
Haloarchaeal transcription factor B phylogeny. Unrooted phylogenetic tree of haloarchaeal transcription factor B (TFB) homologs. Green - TfbC/G, magenta - TfbB/D/F, blue - TfbE, red - TfbA, black - unassigned. Number of sequences and species represented in each clade are shown in parenthesis. Bootstrap support values over 30% are shown for major clades. Branch marked with asterisk is truncated. See Dataset S5 for tree file.

We examined the evolutionary history of haloarchaeal TBP and TFB homologs in order to understand their potential impact on environmental response. Phylogenetic distribution of paralog classes suggest that expansions of the TFB family are ancient, with several duplications occurring prior to haloarchaeal diversification (Figure 9, Dataset S5, Figure S9, Dataset S6). Homologs of five of the seven TFB paralogs from the model haloarchaeon *Halobacterium* sp. NRC-1 were present in at least 79 of 80 sequenced isolates, while another (*tfbA*) was present in 74 isolates. The remaining paralog, *tfbE*, was found in only 39 of the 80 genomes sequenced, suggesting either that this paralog emerged from a relatively late gene duplication event, or has been lost from a large number of genomes.

**Figure 10:**
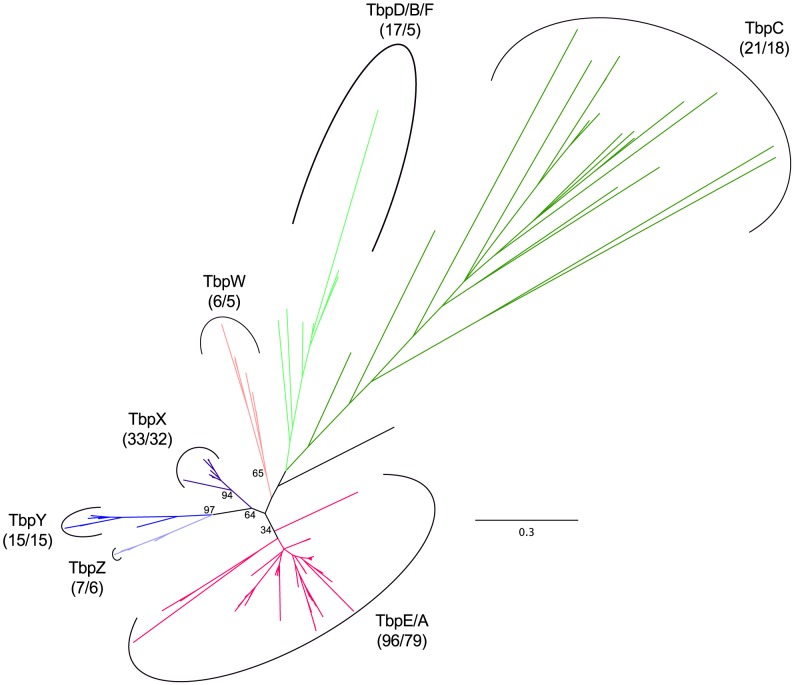
Haloarchaeal TATA-binding protein phylogeny. Unrooted phylogenetic tree of haloarchaeal TATA-binding protein (TBP) homologs. Dark green - TbpC, light green - TbpD/B/F, magenta - TbpE/A, salmon - TbpW, dark purple - TbpX, blue – TbpY, light purple – TbpZ, black - unassigned. Number of sequences and species represented in each clade is shown in parenthesis. Bootstrap support values over 30% are shown for major clades. See Dataset S7 for tree file.

Evolutionary expansion of the TBP family appears to be a more recent phenomenon, with three haloarchaeal lineages showing distinct patterns of duplication and divergence (Figure 10, Dataset S7, Figure S10, Dataset S8). Phylogenetic distribution of TBP paralogs suggests that the ancestral haloarchaeal TBP was most similar to *tbpE* of *Hbt*. sp. NRC-1, with only one species (*Natrinema pallidum*) apparently lacking this homolog. We speculate that this gene may be present at a contig boundary in the assembly for this organism (which consists of 116 contigs), and may later be uncovered by additional sequencing. The ancestral nature of the *tbpE* homolog is also supported by it being the only TBP in the natural TBP knockout strain *Halobacterium salinarum* PHH4 [77].

The previously recognized *tbpD/B/F* expansion appears to be limited to *Halobacterium* species and three haloalkalitolerant Clade 1 haloarchaea (*Natrialba aegyptia, Natrialba taiwanensis,* and *Natrinema pellirubrum*). In addition to this well-known expansion in the *Halobacterium* clade, we have uncovered two additional clade-specific diversifications. The smaller of these two expansions appears to be the result of a single gene duplication event (giving rise to *tbpW*) at the base of the *Halococcus* genus. An additional expansion has occurred near the base of the Clade 2 haloarchaea, with each species possessing at least one (*tbpX*) and in the *Haloferax* genus, up to three (*tbpX*, *tbpY*, and *tbpZ*) TBP homologs derived from this duplication event.

Finally, our analysis of haloarchaeal TBPs revealed a large number of *tbpC*-like homologs. For eukaryotes and archaea, TATA-binding protein normally consists of two domains derived from a duplication event. In each domain, DNA-binding is dependent upon a pair of intercalating phenylalanines [78]. In *Halobacterium* spp., the *tbpC* gene has lost the N-terminal phenylalanine pair, while retaining the C-terminal pair. The *tbpC* gene is easily knocked-out in *Hbt*. sp. NRC-1 [76] and was not detected at the transcriptional level under any growth condition tested in *Hbt*. *salinarum* PHH1 [77]. These data suggest that TbpC may be either nonfunctional or may play a very specialized role in transcriptional regulation under non-laboratory conditions. It is unclear whether defunctionalization may be a result of loss of DNA-binding ability by the N-terminal TBP domain or if loss of the intercalating phenylalanines may be part of an overall loss of function resulting from relaxed selective pressure. Our phylogenetic analysis grouped 21 TBP homologs from 18 species with *Halobacterium* spp. *tbp*C, of which 18 sequences were missing the N-terminal phenylalanine pair, one was missing a single phenylalanine from the N-terminal pair, and two possessed all four phenylalanine residues. Interestingly, sequences missing only the C-terminal phenylalanines, and several sequences missing the N-terminal pair, were not grouped with the *tbp*C homologs, suggesting formation of this clade is not merely an artifact of long-branch attraction. Collectively, this evidence suggests multiple losses of DNA-binding ability in either the N-terminal or C-terminal TBP domain (presumably, sequences having lost both pairs of DNA-intercalating phenylalanines have lost transcription factor function).

Specialization of haloarchaeal general transcription factor paralogs has been implicated in regulating response to a number of environmental perturbations, including variations in temperature, salinity, pH and concentration of heavy metals [75]. Understanding the complicated history of haloarchaeal TBP and TFB diversification will facilitate design of evolutionarily informed experiments for investigating the contribution of general transcription factor paralogs to fitness in the dynamic environments in which these species live.

### Bioinformatic applications of this dataset

In addition to the applications we have already discussed (primarily focused on learning about osmoadaptation), our dataset has the power to address diverse problems in genomics, metagenomics, and other areas of bioinformatics. Here we illustrate some examples highlighting the diverse applicability of our dataset.

#### Genera-level metagenomic profiling of saline environments

The first step in understanding a complex community is often determining what phylogenetic groups are present. This task may be facilitated by use of molecular markers – genes unique to and universal within a specific clade (following the example of [20]). We have developed a set of molecular markers for three deeply sequenced haloarchaeal genera (the *Haloarcula*, *Halorubrum* and *Haloferax*), and provide a list of these protein families as well as the protein sequences, alignments and hidden Markov models for each proposed molecular marker in our Data Dryad package [67]. These markers may be integrated into metagenomics platforms such as Phylosift [79], for mining of metagenomic datasets from hypersaline environments. In addition, we provide a more extensive list of genera-specific protein families for 23 haloarchaeal genera (Dataset S9). This list includes any protein family for which members are found only within a specific genus, regardless of copy number or universality within the genus. Ranging from 1,456 unique protein families (*Haloferax*) to only two (*Halopiger*), this list provides a rich resource for exploring genera-specific haloarchaeal biology.

#### Improvements to automated gene calling algorithms

Automated genome annotation programs inevitably result in both spurious and missed gene calls [80]. The two annotation programs used in our analysis, NCBI's PGAAP [14] and the GLIMMER gene caller used in the RAST annotation pipeline [13] have been shown to exhibit this behavior [80]. As previously reported [80], we found that analyzing gene neighborhoods from closely related species can be helpful in detecting gene call errors. We identified an example of a highly conserved gene neighborhood where three of the fifteen sequenced *Haloferax* genomes had a gap in a location where a gene call was expected based on the other *Haloferax* genomes (Figure S11). Manual investigation revealed a possible protein-coding region for each of these three species, which aligned at the protein level with the twelve called genes (Figure S12). We were also able to identify, in the *Haloarcula* genus, regions with possible missing gene calls, erroneous start and stop sites, and a short hypothetical protein which may represent a spurious gene call (Figure S13). These examples highlight the potential for deep phylogenetic sampling in conjunction with gene neighborhood analysis, to aid automated gene calling programs. Genomic neighborhood analysis of closely related species has already been implemented in the genome annotation modification tool GenePrimp [80].

#### Improvements to automated gene annotation algorithms

Deep phylogenetic sampling also has the potential to improve gene annotation. By investigating patterns of protein family presence and absence across the haloarchaea, we were able to form testable hypotheses about the function of otherwise unannotated genes. We grouped protein families with similar phylogenetic distributions using hierarchical clustering. In cases where several protein families in a cluster were annotated as being involved in a particular process, unannotated members of that cluster were hypothesized to also be involved in this process. Two examples are discussed here, with additional examples in Dataset S10. First, a group of nine unannotated proteins were found to have similar phylogenetic distribution to six annotated redox proteins, including two menaquinol cytochrome-c reductases, two ferredoxins, a ubiquinol cytochrome-c reductase and a sulfite oxidase. Due to this redundancy, we speculate that this cluster represents more than one respiratory chain, one possibly using a sulfite as an electron donor (Figure S14). We hypothesize that several of the nine unannotated protein families in this cluster are also electron transport chain components. Secondly, using this method, we have identified two potentially novel members of the cobalamin biosynthesis pathway (Figure S15). A protein family cluster was identified in which 15 of 16 annotated members are involved in cobalamin biosynthesis. We therefore hypothesize that the two unannotated members of this cluster are also involved in this process. We present these cases as specific examples of the potential utility of phylogenetic profiling in improving automatically generated gene annotations. Additional examples can be found in Dataset S10 and Figures S16–S22.

In this section we have illustrated a few of the ways in which deep sequencing projects such as ours can benefit the genomics and metagenomics communities. These examples should not be taken as a complete set of problems to which our dataset may be applied, but rather as an indication of its broad utility and an invitation to the community to explore and utilize this data. To facilitate its widespread use, we have made the data available not only through the NCBI genome repository, but also through an SQL database and as a loadable ”popular genome set” within the visualization tool JContextExplorer [17]. We believe that lessons gleaned from explorations of this dataset will continue to enrich the genomics, halophile, archaeal, evolutionary and broader biology research communities for years to come.

## Materials and Methods

### Strain growth and DNA isolation

Strains were acquired as desiccated cells from the American Type Culture Collection (ATCC) in Manassas, Virginia, USA; the Leibniz Institute DSMZ German Collection of Microorganisms and Cell Cultures (DSM) in Braunschweig, Germany; and the Japan Collection of Microorganisms (JCM) in Ibaraki, Japan, as indicated in Table S2. Cells were rehydrated in recommended media according to culture collection center protocols and grown to stationary phase at 37°C in liquid culture. Genomic DNA was harvested with Wizard Genomic DNA purification kit (Promega).

### Sequencing, assembly, and annotation

Sequence libraries were constructed using a combination of standard fragmentation (200–500 bp), mate-pair fragmentation (6 Kbp), and PCR-free transposon-mediated insertion of sequencing primers (Epicentre Nextera) [81]. Transposase was purified from E. coli BL21 (DE3) containing the cloning vector pWH1891. For mate-pair libraries, 6 Kb pair-end libraries were constructed and the terminal 50 bases of each end were sequenced, according to standard protocols. Additional libraries were constructed by combining DNA fragments from haloarchaeal species distantly enough related to enable unambiguous assignment of non-barcoded reads (“SOUP”). All sequencing was performed on Illumina HiSeq and GAII platforms. The paired-end information and trimming information were specified using annotation strings on the description line of the reads. Reads were assembled using the a5 pipeline [18]. Following assembly, genomic DNA contamination arising from transpose-mediated library preparation was removed by searching assembled reads against a local BLAST database consisting of E. coli BL21 (DE3) genomic DNA and the cloning vector pWH1891. BLAST hits with an E-value ≤ 10−20 were considered to be significant matches and candidates for contamination. Contigs with matches covering ≥80% of the contig length and contigs ≤1 Kbp with matches covering any portion of the contig were treated as contamination and discarded from further analysis. Long contigs for which only a small portion of the contig matched to the local BLAST database were also discarded if there were either no annotated features, or if the annotated features were E. coli genes. These criteria resulted in a total of 497 contigs equaling 265.57 Kbp being removed from the final assemblies.

A dual annotation pipeline was implemented in order to take advantage of the strengths of different existing automated annotation tools. Assembled genomes were first submitted to the Rapid Annotation using Subsystem Technology (RAST) server at the National Microbial Pathogen Data Resource. RAST-based gene calls and annotations were used for building of protein families, core genome and pan genome analyses, phylogenetic reconstruction, analysis of general transcription factor expansions, GC-bias analysis, building of molecular marker sets and phylogenetically informed re-annotation. These annotations can be accessed as a loadable “popular genome set” through the genome context viewer JContextExplorer [17] as well as a custom MySQL database (see Text S1 for instructions). The RAST annotation system was particularly useful in enabling comparison of our genomes with previously sequenced haloarchaea, by allowing standardization via rapid reannotation of existing genomes.

In addition, the newly sequenced genomes were annotated using NCBI's Prokaryotic Genome Annotation Pipeline (PGAAP) [14]. PGAAP gene calls and annotations were used for COG analysis, proteome acidification calculations, and genera-based genomic feature comparisons. These annotations can be accessed through the NCBI website using the accession numbers listed in Table S2 as well as through our custom MySQL database.

### Phylogeny

A phylogeny was constructed for all archaeal genomes available through the Integrated Microbial Genomes database along with our sequenced haloarchaea using a concatenated set of 40 conserved marker genes [20]. Peptide sequences were downloaded for all archaeal genomes from the IMG 4.0 database on January 4, 2013. HMM profiles of 40 bacterial and archaeal PhyEco markers were searched against these peptide sequences. We excluded genomes with less than 35 of these markers, as well as duplicate genomes which were included in both our analysis and the IMG database. A total of 151 IMG archaeal genomes and all 80 haloarchaeal genomes were included in the phylogenetic tree building. For each PhyEco marker family, only single-copy members from the genomes were included. Independent alignments were built using MUSCLE [82] for each gene families and then concatenated. A phylogenetic tree was built from the concatenated alignment using PHYML 3.0 [83] with the LG substitution model. Tree topology and branch lengths were optimized by the program and aLRT SH-like statistics was used for branch support estimation. The R package ape was used to remove duplicate genomes [84] and the final tree was visualized using FigTree [85]. The tree files are available as Dataset S3 (all archaea) and Dataset S11 (haloarchaea only).

### Protein families

Homologous protein families were constructed using a Markov clustering-based approach. First, an all-vs-all BLASTp search of all protein coding genes called by RAST in the 80 haloarchaeal genomes was conducted using an E-value cutoff of 10^−5^, soft masking, and the Smith-Waterman alignment algorithm. These options have been shown to improve homolog detection over other BLAST methods [86]. To enable detection of large homology groups, the number of returned matches was set at 100,000. To remove spurious, domain-level matches, BLAST results were post-filtered and matches retained only if bi-directional query-match coverage was ≥80%, and each sequence was ≥75% of the length of the other. Matches with E-values>10^−10^ were also excluded at this stage (Text S2).

BLAST results were then clustered into homology groups using the clustering algorithm TRIBE-MCL [16], which utilizes the protein-protein similarity network implicit in the BLAST scores. This method has the benefit of building inclusive homology families including orthologs, xenologs and both in- and out-paralogs, unlike reciprocal best BLAST methods which often overlook complex gene relationships in the search for one-to-one ortholog mapping. To determine an appropriate inflation parameter for TRIBE-MCL clustering, homology clusters derived from inflation parameters ranging from 1.4 to 8.0 were compared and benchmarked using manually curated protein families for haloarchaeal TATA-binding proteins and opsins. The inflation parameter I = 2.5 was selected, as it most closely recapitulated known ortholog/paralog relationships for the selected protein families. A total of 17,591 homology clusters (protein families) were obtained with this method, comprising 276,364 of 303,129 total haloarchaeal proteins. The remaining proteins were singletons, lacking significant sequence similarity to other sequences in the dataset. These sequences may represent species-specific innovation, recent gene influx from horizontal gene transfer, or simply a lack of sequencing depth along some sampled haloarchaeal lineages.

### Rarefaction curves

Random subsets of haloarchaeal genomes (ranging from zero to eight genomes) were selected from the set of RAST-annotated haloarchaea, using the Java Samplers package [87]. For each selection the following quantities were tabulated: (1) the number of unique COGs, (2) the number of unique TRIBE-MCL determined homology clusters, and (3) the number of unique non-singleton TRIBE-MCL determined homology clusters. Ten thousand random selections were performed for each sample size and the average number of unique protein families obtained for each sample were plotted in Microsoft Excel (Figure 3).

### Reanalysis of bacterial gene mass acquisition study

The 1,479 protein families used in the analysis described in [25] were obtained from the authors. All genes were renamed with an “E”, “A”, or “H” tag prepended to their original NCBI gene ID number, according to their taxonomic classification as, respectively, (eu)bacteria, archaea, or haloarchaea. Proteins from 65 haloarchaeal species not included in the original study were assembled into a set of “additional haloarchaeal proteins”, and named with an “H” tag and unique ID number. Five haloarchaeal genomes in our set were either different strains of a species included in the original study, or a re-sequencing of the same strain, and so were excluded from re-analysis. Gene trees were re-constructed for all 1,479 of the original protein families, using the alignment program MAFFT [88], with command line options –legacygappenalty, –anysymbol, and –quiet; the protein model determination tool ProtTest [89], using command line options –all-matrices, all-distributions, -S 2 –F –t1; and the gene tree creation tool PhyML [83]. All command line options were obtained from the authors of [25]). Gene trees were determined to be single transfers from the bacteria only if (1) all haloarchaeal homologs formed a monophyletic group and (2) this monophyletic group rooted with bacterial rather than archaeal homologs. For gene trees matching these criteria, corresponding protein families were extended to include homologs predicted from the set of “additional haloarchaeal proteins”, using BLAST with an E-value cutoff of 10^−10^ and a minimum percent identity of 30%. Gene trees were generated for these extended protein families using the parameters described above. Extended gene trees were re-evaluated according to the criteria described above to determine whether they could still be classified as single bacterial transfers. Some (17.2%) of the extended protein families contained a sufficiently large number of members such that determination of gene trees was infeasible, and so were excluded from re-analysis. Gene tree classification results are provided in Table 1. Phylogenetic trees visualized in Figures S4 and S3 were made using Phyfi [90].

### Phylogenetic distribution of osmoadaptation genes

A list of 74 genes involved in ion transport and compatible solute transport or synthesis was compiled. Representative protein sequences for each of the selected genes were obtained by searching the NCBI protein database by annotation (Dataset S12). When available, sequences from the model organisms *Escherichia coli* and *Bacillus subtilis* were selected. Genes with “MM” prefix derive from *Methanosarcina mazei* Go1. BLASTp searches for these query sequences were conducted against a local BLAST database containing 80 haloarchaeal genomes. In order to obtain all potential homologs, the maximum target sequences parameter was set to 100,000 and an E-value cutoff of 1 was used. Resulting matches were then filtered using custom perl scripts to remove spurious domain-level matches by requiring a bidirectional query-target coverage of ≥80%, and a bidirectional query-target length of ≥75%. To reduce the number of non-specific family-level matches (for example, to differentiate between the closely related Trk H^+^/K^+^ and Ktr Na^+^/K^+^ symporters), sequences were also filtered to remove matches with an E-value of>1e^−20^. A presence/absence matrix was constructed from these filtered BLAST results using custom bash and R scripts, and visualized in iTOL [91], [92]. Genes were grouped into functional categories based on substrate specificity and directionality for ion transporters, substrate imported for compatible solute transporters, or product for compatible solute biosynthesis proteins. As 20 of the query genes were not detected in the set of investigated species, only 54 columns appear in Figure S5. Gene presence/absence data was scaffold on the multi-marker concatenated haloarchaeal phylogeny (Dataset S11).

### Genera-based comparisons

Whole-genome feature data for the 59 newly sequenced, PGAAP-annotated haloarchaea were retrieved from an in-house MySQL database using custom scripts [67]. For each organism the genome size, number of contigs, percent G+C content, percent coding, number of coding regions, and number of insertion sequence elements were extracted. Genomes were organized into genera and box plots for each of the above traits were drawn for each genus using MATLAB's Statistics toolbox [93]. Images were exported using the supplementary export_fig function [94]. See Figure S23.

### Isoelectric point analysis

All isoelectric point analyses were carried out exclusively on the PGAAP gene call set. The pI of every protein coding sequence was computationally predicted using the protein sequence isoelectric point prediction method included in the BioJava framework [95]. pI histograms for each genome were computed using MATLAB's statistics toolbox [93], sorting proteins into 100 equally spaced bins ranging from a pI of 2.0 to 13.0. All proteins with a pI of 7.5 or greater were collected and a list of all represented gene annotations was procured. For each gene annotation in this list, all instances were tabulated, including the number of instances with a pI greater than or equal to 7.5. A table showing these results, sorted by frequency of instances with high pI can be found in Table S5. All proteins annotated as ribosomal subunits were collected, and all subunits which could confidently be mapped to experimentally characterized structures for the large (1QVG) [96] and small (1FKA) [97] ribosomal subunits were mapped. Subunits with a pI>7.5 in 60% or more of cases were colored red, and subunits with a pI<7.5 in 60% or more of cases were colored blue. Any subunits which could not be confidently mapped or which had variable pI across species were left the default color of green. All mappings were done using the PyMOL protein visualization program [98].

Comparative acidification visualizations of TBP and ribosome elongation factor α-1 proteins for haloarchaea and non-halophilic organisms were generated using the SWISS-MODEL web interface [99] and 1D3U [100] and 3VMF [101] as the structural templates, respectively.

### GC bias analysis

To investigate localized variations in G+C content across each genome, custom scripts were created (Text S3 & S4). First, %G+C was calculated for 100 bp windows across the genome, with a 20 bp step size. The terminal <100 bps of each contig were not included in calculation, so as not to artificially inflate the final G+C calculation for each contig. Windows with>10% ambiguous nucleotides were assumed to have %G+C equal to the contig mean. Plots were generated showing a) the overall mean G+C percent for the entire genome as a horizontal green line, b) the contig boundaries as vertical red lines, c) the mean G+C percent for each contig as horizontal blue lines, and d) all G+C percentages for individual 100 bp segments as a black line [67]. Steps where the mean %G+C inflects away from the local mean were calculated using the R changepoint package [102], and these changepoints were plotted over the stepwise G+C percentages as vertical green lines [67]. After manual curation of changepoints for each species, annotated features between changepoints were retrieved from each species' general feature format (GFF) file. Ten species were excluded from this analysis, either due to having a very large number of contigs, or having no observable inflections in local mean G+C content. For a list of excluded organisms and reasons, see Text S1. The set of features extracted from regions of abnormal G+C content were analyzed for enrichment of homologous protein families (TRIBE-MCL clusters). The frequency of each protein family in the abnormal G+C feature set was compared to its frequency in the entire genome set, and families with at least eight-fold enrichment were investigated further. To avoid including families which were enriched artificially by having a small number of members which are all present in abnormal G+C regions, families with fewer than five members were excluded from enrichment analysis.

### General transcription factors

A local BLAST database was constructed using RAST-derived gene calls for the 80 haloarchaea. This database, and the NCBI non-redundant protein database (as of October 30^th^, 2012), were queried for homologs to the archaeal and eukaryotic general transcription factors TATA-binding protein (TBP) and transcription factor B/IIB (TFB) using a query set of 19 curated TBP homologs and 27 curated TFB homologs from six archaeal and one eukaryotic species (Datasets S18 & S19). A BLASTp search was conducted (BLAST+2.2.27) using a maximum expect value of 10^−5^ and maximum target sequence of 100,000. For each set of homologs, a multi-sequence alignment was constructed using MUSCLE v3.8.31 [82] and manually curated to remove poorly aligning regions. Highly divergent and fragmentary sequences were manually removed. One thousand bootstrapped alignments were created by resampling the curated alignment using Seqboot in the Phylip toolkit (v3.69) [103]. Phylogenetic trees were created for each bootstrapped alignment using FastTree v2.1.5 SSE3 [104] and a consensus tree was produced by comparing these trees to a guide tree, also constructed using FastTree, using the tree comparison tool CompareToBootstrap.pl [105]. For all clades in the guide tree, the fraction of bootstrapped trees in which that clade appeared were tabulated and recorded as the bootstrap support values for that clade. Because this approach is limited in that only clades appearing in the initial guide tree are considered, we also constructed a consensus tree with the Consense program in the Phylip package [105] using the extended majority rule option. Each clade in the resulting consensus tree represents the most frequent grouping of those species in the 1000 bootstrap replicate trees, independent of any guide tree. For Consense tree files, see Datasets S20–S23. Branch lengths and node labels in Consense tree represent the number of bootstrapped tree replicates for which each clade was observed, rather than sequence divergence. Trees were visualized in FigTree [85].

### Genera-specific protein families

Marker genes were identified by the following procedure: Homology clusters were identified that were (1) universal to the genus of interest, (2) not found in any other haloarchaeal species, and (3) single copy. Homologs were aligned using MUSCLE [82] with default parameters, and a hidden Markov model was generated from each alignment using HMMer [106] with default parameters. Additionally, a list of marker homology clusters was generated, as were the genomic coordinates for each marker gene [67]. This list may be loaded into JContextExplorer [17] as a custom context set.

### Improvements to automated gene calls

Putative missed gene calls were identified using JContextExplorer [17], with the Haloarchaea genome set loaded. To retrieve the context surrounding Tribe4688 (with gene calls in 12 *Haloferax* species) a “Between” context set was created with a 5 Kbp limit, and the query “966; 458” was carried out under “Cluster Search”. Regions were visualized by selecting all instances and pushing the “View Contexts” button. This produced a set of either two or three gene groupings, in the order Tribe458 (always) – Tribe4688 (sometimes) – Tribe966 (always). The sequence between instances of Tribe458 and Tribe966 in the three *Haloferax* species that did not contain an instance of Tribe4688 was extracted, and every contiguous region of 117 nucleotides was translated into a protein using MATLAB's bioinformatics toolbox [107]. Each match was manually compared to the gene members of protein sequences of Tribe4688, and three matches were identified, one in each of the three organisms lacking an instance of Tribe4688 (*Haloferax alexandrinus, Hfx. elongans,* and *Hfx. sulfurifontis*). The sequence alignments were visualized, with amino acids colored according to the properties of their side chains. To retrieve the region of poor gene call consistency in *Haloarcula*, the same between context set was used with a 5 Kbp limit, with the query “254; 369” under “Cluster Search”. Genomic segments were visualized by selecting all instances and pushing the “View Contexts” button.

### Improvements to automated gene annotations

A presence/absence matrix was constructed for each TRIBE-MCL protein family across the 80 haloarchaea and transformed into a distance matrix using Euclidean distances. Hierarchical clustering of this distance matrix was performed using Mev [108], and the resulting matrix was visualized. Groups of protein families which clustered together have a similar phylogenetic dispersal pattern, and may therefore perform functionally related tasks. This distribution was manually interrogated to find groups of clustered protein families with functionally related annotations. Unannotated protein families in that cluster were then hypothesized to also be involved in that task.

## Supporting Information

Figure S1 **Size distribution of homologous protein families.** Density distribution of number of members per homologous protein family (cluster) predicted by TRIBE-MCL (inflation parameter  = 2.5). Cluster size distribution is bimodal, with the majority of protein families being very small (≤15 members) and a small group of clusters possessing ∼80 proteins.
(PDF)

Figure S2 **Number of species per homologous protein family.** Density distribution of number of species per homologous protein family (cluster) predicted by TRIBE-MCL (inflation parameter  = 2.5). Haloarchaeal core protein families are those represented by the minor mode at the right edge of the distribution.
(PDF)

Figure S3 **New clade appears in haloarchaea-extended gene tree.** The gene tree for protein family 8319 from [25] is consistent with a basal HGT event from bacteria into the haloarchaeal root (A). However, after adding predicted homologs from 65 additional haloarchaeal genomes, the gene tree includes an new group of haloarchaeal homologs not monophyletic with the previously identified group (B, red circle). This is inconsistent with the basal HGT hypothesis, and could indicate an HGT event preceding that identified by [25] for this gene. E =  (eu)bacteria, H =  haloarchaea. Numerical IDs not containing a dash refer to NCBI gene IDs, those containing a dash refer to proteins from the set of newly-sequenced haloarchaea. Collapsed nodes in (B) contain exclusively haloarchaea.
(PDF)

Figure S4 **Potential secondary HGT event in haloarchaea-extended gene tree.** The gene tree for protein family 12957 from [25] is consistent with a basal HGT event from bacteria into the haloarchaeal root (A). However, after adding predicted homologs from 65 additional haloarchaeal genomes, the gene tree includes a single haloarchaeal homolog which groups with bacterial rather than the remaining haloarchaeal homologs (B, red circle). This is inconsistent with the basal HGT hypothesis, and could indicate a subsequent HGT event, following that identified by [25] for this gene. E =  (eu)bacteria, H =  haloarchaea. Numerical IDs not containing a dash refer to NCBI gene IDs, those containing a dash refer to proteins from the set of newly-sequenced haloarchaea.
(PDF)

Figure S5 **Phylogenetic distribution of genes involved in ion transport, and compatible solute transport and biosynthesis.** Presence/absence pattern of 54 genes involved in ion transport, compatible solute transport or compatible solute biosynthesis. Homologs were detected by BLAST searches, filtered according to metrics given in Materials and Methods, and presence/absence data superimposed on haloarchaeal phylogenetic tree (Dataset S11). Gray squares represent presence of a particular homolog. Homologs are grouped by functional category - listed across the top of the figure. Each column represents one homolog – listed along the bottom of the figure. Tree roots in the Methanocella (not shown). Genes in search set with no haloarchaeal homologs are not shown (see Datasets S4 & S12).
(PDF)

Figure S6 **Pattern of TATA-binding protein acidification. Comparative acidification of TATA-binding protein (TBP) binding to DNA for three non-halophilic archaea (left, black text) and two haloarchaea (right, red text).** Acidified regions are shown in red, while basic regions are blue. Close-up of DNA-binding site reveals that these regions remain unacidified in the haloarchaea. The opposite side of the TBP protein (bottom), which does not interact directly with the DNA, demonstrates significant acidification in haloarchaea. *A. fulgidus*  =  *Archaeoglobus fulgidus*, *M. hungatei*  =  *Methanospirillum hungatei*, *M. paludicola*  =  *Methanocella paludicola*, *Nmn*.  =  *Natronomonas*, *Har*.  =  *Haloarcula*. Structural model used was that for *Pyrococcus woesei* (ID3U) [100].
(PDF)

Figure S7 **Pattern of ribosome elongation factor α-1 acidification.** Comparative acidification of tRNA-binding versus non-tRNA-binding side of ribosomal elongation factor α-1 for five non-halophilic archaea (left) and three haloarchaea (right). Acidified regions are shown in red, while basic regions are blue. No acidic enrichment was detected for the tRNA-binding face (top), while acidic enrichment was apparent in solvent-exposed face of haloarchaeal homologs (bottom). *P. torridus*  =  *Picrophilus torridus*, *A. fulgidus*  =  *Archaeoglobus fulgidus*, *M. maripaludis*  =  *Methanococcus maripaludis*, *M. acetivorans*  =  *Methanosarcina acetivorans*, *M. mazei*  =  *Methanosarcina mazei*, *Hqt.*  =  *Haloquadratum*, *Hbt.*  =  *Halobacterium*, *Nmn.*  =  *Natronomonas*. Structural model used was that for *Aeropyrum pernix* K1 (3VMF) [101].
(PDF)

Figure S8 **Representative selected changepoints for G+C content analysis. Representative G+C content plots for (A) *Haloferax mediterranei*, (B) *Halorubrum litoreum*, (C) *Haloarcula argentinensis*, and (D) *Natrialba aegyptia* showing manually curated changepoints.** Each of these four species is a representative from the four most populous genera in the set, which collectively contain 66% of the organisms in the study. Black line represents calculated G+C percent for each 100 bp window and vertical green lines represent points of inflection in the mean G+C percent which were selected as changepoints. Genomic regions between changepoints were taken as regions of abnormal G+C content, extracted and analyzed for enrichment of functional groups. The horizontal axis displays the number of 20 bp steps taken along the genome. Representative species selected correspond to those shown in Figure 7.
(PDF)

Figure S9 **Universal transcription factor B phylogeny.** Unrooted phylogenetic tree of transcription factor B (TFB) homologs from NCBI's non-redundant protein database and recently sequenced haloarchaeal genomes. Red - haloarchaea, brown - other euryarchaeota, bright purple - crenarchaeota, yellow - thaumarchaeota, dark blue - other/unclassified archaea, salmon - metazoa, bright green - fungi, hot pink - viridiplantae, orange - other eukaryotes. See Datasets S6 for tree file.
(PDF)

Figure S10 **Universal TATA-binding protein phylogeny.** Unrooted phylogenetic tree of TATA-binding protein (TBP) homologs from NCBI's non-redundant protein database and recently sequenced haloarchaeal genomes. Red - haloarchaea, brown - other euryarchaeota, bright purple - crenarchaeota, yellow - thaumarchaeota, dark blue - other/unclassified archaea, dark green - chromalveolata, salmon - metazoa, dark purple - cryptophyta, lavender - excavata, bright green - fungi, hot pink - viridiplantae, orange - other eukaryotes. See Dataset S7 for tree file.
(PDF)

Figure S11 **Genomic regions in *Haloferax* suggestive of a missed gene call.** Analysis of genomic neighborhoods for 15 *Haloferax* species revealed a small hypothetical protein (Tribe4688, magenta) between a hypothetical protein (Tribe966, light blue) and a protein annotated as co-occurring with transport systems (Tribe458, dark blue), in most species. However this gene was not called in three species. The high degree of genomic context conservation suggests that the RAST gene caller may have missed a copy of Tribe4688 in these species. Species name is shown in the upper left of each segment. Black arrow  =  forward strand, red arrow  =  reverse strand. Grey boxes represent genes not involved in this analysis.
(PDF)

Figure S12 **Alignments of Tribe4688 with predicted missed gene calls.** The complete protein sequence is shown for every instance of Tribe4688 in the *Haloferax*, with three additional protein sequences identified in the gap regions where a protein was expected to be found. The homologs sorted into two groups, with the three proposed missing genes (red boxes) sorting among these groups, suggesting that gene caller misses were not based on sequence conservation thresholds. The high degree of sequence conservation and match in length strongly suggests that the observed absence of this gene in *Hfx. alexandrinus*, *sulfurifontis*, and *elongans* is the result of gene caller errors rather than a biological phenomenon.
(PDF)

Figure S13 **A region of poor gene call consistency in *Haloarcula spp*.** Genomic regions are shown between Tribe254 and Tribe369 when these genes fall within 5 Kb. Only four of the intervening genes are consistent across all six *Haloarcula* species (245, magenta; 5724, dark blue; 6141, cyan; and 369, red). Tribe7403 (white) may be an example of a missed gene call (absent in two species) (B). This may also be the case for Tribe9660 (purple)/Tribe18431 (grayish-blue), which is missing in three species and apparently truncated in one (A). Tribes 6775 (orange), 18947 (lime green), 8276 (pink), 12986 (light yellow), and 19374 (green) may be cases where translation start and stop sites have not been correctly called (C and D). Finally, the short, hypothetical protein Tribe18194 (dark green) may be a spurious gene call (E). This region serves as an example to highlight the necessity for improvements in automated gene call prediction.
(PDF)

Figure S14 **Phylogenetic profiling assisted gene annotation (electron transport chain).** Phylogenetic distribution patterns of unannotated genes assist with prediction of gene function. Cases where unannotated genes have similar phylogenetic distribution to a number of genes with predicted functions allow for hypotheses to be made about the functions of unannotated group members. Visualization and hierarchical clustering of protein presence and absence data was done using Mev [108]. Black represents absence and red represents presence of a protein family. Consense annotations and numbers corresponding to TRIBE-MCL protein families are shown on the right.
(PDF)

Figure S15 **Phylogenetic profiling assisted gene annotation (cobalamin metabolism). **Phylogenetic distribution patterns of unannotated genes assist with prediction of gene function. Cases where unannotated genes have similar phylogenetic distribution to a number of genes with predicted functions allow for hypotheses to be made about the functions of unannotated group members. Visualization and hierarchical clustering of protein presence and absence data was done using Mev [108]. Black represents absence and red represents presence of a protein family. Consense annotations and numbers corresponding to TRIBE-MCL protein families are shown on the right.
(PDF)

Figure S16 **Phylogenetic profiling assisted gene annotation (CRISPR-associated proteins).** Phylogenetic distribution patterns of unannotated genes assist with prediction of gene function. Cases where unannotated genes have similar phylogenetic distribution to a number of genes with predicted functions allow for hypotheses to be made about the functions of unannotated group members. Visualization and hierarchical clustering of protein presence and absence data was done using Mev [108]. Black represents absence and red represents presence of a protein family. Consense annotations and numbers corresponding to TRIBE-MCL protein families are shown on the right.
(PDF)

Figure S17 **Phylogenetic profiling assisted gene annotation (arsenic resistance).** Phylogenetic distribution patterns of unannotated genes assist with prediction of gene function. Cases where unannotated genes have similar phylogenetic distribution to a number of genes with predicted functions allow for hypotheses to be made about the functions of unannotated group members. Visualization and hierarchical clustering of protein presence and absence data was done using Mev [108]. Black represents absence and red represents presence of a protein family. Consense annotations and numbers corresponding to TRIBE-MCL protein families are shown on the right.
(PDF)

Figure S18 **Phylogenetic profiling assisted gene annotation (light & redox response).** Phylogenetic distribution patterns of unannotated genes assist with prediction of gene function. Cases where unannotated genes have similar phylogenetic distribution to a number of genes with predicted functions allow for hypotheses to be made about the functions of unannotated group members. Visualization and hierarchical clustering of protein presence and absence data was done using Mev [108]. Black represents absence and red represents presence of a protein family. Consense annotations and numbers corresponding to TRIBE-MCL protein families are shown on the right.
(PDF)

Figure S19 **Phylogenetic profiling assisted gene annotation (nitrate respiration I).** Phylogenetic distribution patterns of unannotated genes assist with prediction of gene function. Cases where unannotated genes have similar phylogenetic distribution to a number of genes with predicted functions allow for hypotheses to be made about the functions of unannotated group members. Visualization and hierarchical clustering of protein presence and absence data was done using Mev [108]. Black represents absence and red represents presence of a protein family. Consense annotations and numbers corresponding to TRIBE-MCL protein families are shown on the right.
(PDF)

Figure S20 **Phylogenetic profiling assisted gene annotation (nitrate respiration II).** Phylogenetic distribution patterns of unannotated genes assist with prediction of gene function. Cases where unannotated genes have similar phylogenetic distribution to a number of genes with predicted functions allow for hypotheses to be made about the functions of unannotated group members. Visualization and hierarchical clustering of protein presence and absence data was done using Mev [108]. Black represents absence and red represents presence of a protein family. Consense annotations and numbers corresponding to TRIBE-MCL protein families are shown on the right.
(PDF)

Figure S21 **Phylogenetic profiling assisted gene annotation (nitrous oxide respiration).** Phylogenetic distribution patterns of unannotated genes assist with prediction of gene function. Cases where unannotated genes have similar phylogenetic distribution to a number of genes with predicted functions allow for hypotheses to be made about the functions of unannotated group members. Visualization and hierarchical clustering of protein presence and absence data was done using Mev [108]. Black represents absence and red represents presence of a protein family. Consense annotations and numbers corresponding to TRIBE-MCL protein families are shown on the right.
(PDF)

Figure S22 **Phylogenetic profiling assisted gene annotation (pH adaptation & K^+^ efflux).** Phylogenetic distribution patterns of unannotated genes assist with prediction of gene function. Cases where unannotated genes have similar phylogenetic distribution to a number of genes with predicted functions allow for hypotheses to be made about the functions of unannotated group members. Visualization and hierarchical clustering of protein presence and absence data was done using Mev [108]. Black represents absence and red represents presence of a protein family. Consense annotations and numbers corresponding to TRIBE-MCL protein families are shown on the right.
(PDF)

Figure S23 **Genera-level comparisons of genomic features (part II).** Number of contigs (A), number of insertion elements (B) and percent coding DNA (C) were extracted from each genome, organized by genus, and boxplots calculated using MATLAB's Statistics toolbox [93]. Boxplots were generated using 25^th^ and 75^th^ percentile as box edges, with median demarcated with horizontal line within box. Genera are ordered by descending number of species sequenced, with the number of species shown in parentheses. Genera with only a single sequenced member are shown as horizontal lines.
(PDF)

Table S1 **Strain isolation information.**
(XLSX)

Table S2 **Genome features and sequencing statistics.**
(XLSX)

Table S3 **Comparison of independently sequenced genomes.** [110]–[115].
(XLSX)

Table S4 **Haloarchaeal core genome.**
(XLSX)

Table S5 **Protein families with high pI members.**
(XLSX)

Table S6 **Protein families enriched in abnormal G+C regions.**
(XLSX)

Dataset S1 **Unannotated proteins dataframe.** Analysis of predicted protein coding regions lacking annotations and belonging to a non-singleton protein family. From left to right, columns represent: protein ID (from RAST gene calls), protein family to which the protein belongs, and number of members, species and genera in that protein family.
(TXT)

Dataset S2 **TRIBE-MCL clusters.** Homologous protein families predicted by TRIBE-MCL clustering of all-vs-all BLAST results. Each line of this file represents one protein family, arranged in order of size. Tribe1 corresponds to first line of file and largest protein family. Families may include xenologs and paralogs as well as orthologs. Protein IDs are from the RAST gene call set. Protein families best explored via JContextExplorer (see Text S1 for detailed instructions).
(TXT)

Dataset S3 **Multi-marker concatenated archaeal phylogeny tree file.** A phylogenetic tree was built from the concatenated alignment of 40 PhyEco markers using PHYML 3.0 with the LG substitution model. Tree topology and branch lengths were optimized by the program and aLRT SH-like statistics was used for branch support estimation. Tree file is in Newick format.
(TXT)

Dataset S4 **Osmoadaptation genes with annotations.** A list of query proteins with functions related to osmoadaptation and their associated annotations.
(TXT)

Dataset S5 **Haloarchaeal TFB CompareToBootstrap tree file.** Manually curated alignment of haloarchaeal transcription factor B protein sequences was resampled 1000 times and trees constructed for each bootstrap replicate using FastTree [104]. The consensus tree was produced by comparing these 1000 trees to a guide tree, also produced using FastTree, using the tree comparison tool CompareToBootstrap.pl [105]. Bootstrap support values for each clade represent the fraction of 1000 bootstrapped trees in which that clade appeared. Tree file is in Nexus format.
(TXT)

Dataset S6 **Universal TFB CompareToBootstrap tree file.** Manually curated alignment of archaeal and eukaryotic transcription factor B protein sequences was resampled 1000 times and trees constructed for each bootstrap replicate using FastTree [104]. The consensus tree was produced by comparing these 1000 trees to a guide tree, also produced using FastTree, using the tree comparison tool CompareToBootstrap.pl [105]. Bootstrap support values for each clade represent the fraction of 1000 bootstrapped trees in which that clade appeared. Tree file is in Nexus format.
(TXT)

Dataset S7 **Haloarchaeal TBP CompareToBootstrap tree file.** Manually curated alignment of haloarchaeal TATA-binding protein sequences was resampled 1000 times and trees constructed for each bootstrap replicate using FastTree [104]. The consensus tree was produced by comparing these 1000 trees to a guide tree, also produced using FastTree, using the tree comparison tool CompareToBootstrap.pl [105]. Bootstrap support values for each clade represent the fraction of 1000 bootstrapped trees in which that clade appeared. Tree file is in Nexus format.
(TXT)

Dataset S8 **Universal TBP CompareToBootstrap tree file.** Manually curated alignment of archaeal and eukaryotic TATA-binding protein sequences was resampled 1000 times and trees constructed for each bootstrap replicate using FastTree [104]. The consensus tree was produced by comparing these 1000 trees to a guide tree, also produced using FastTree, using the tree comparison tool CompareToBootstrap.pl [105]. Bootstrap support values for each clade represent the fraction of 1000 bootstrapped trees in which that clade appeared. Tree file is in Nexus format.
(TXT)

Dataset S9 **Genera-specific protein families.** A list of TRIBE-MCL protein families limited to, but not necessarily universal within, each haloarchaeal genus with sequenced members in this study.
(TXT)

Dataset S10 **Reannotations based on phylogenetic profiling.** A list of hypothesized gene functions for unannotated genes. Proposed reannotations are based on unannotated genes having similar phylogenetic distribution to a number of annotated genes in the same functional category as discovered via hierarchical clustering of protein presence and absence data across all 80 sequenced haloarchaeal genomes.
(TXT)

Dataset S11 **Haloarchaeal phylogeny for use with JContextExplorer.** A version of the multi-marker archaeal phylogeny (Dataset S3) where species not included in the set of 80 haloarchaea used in this study were removed using the R package ape. This tree file can be loaded into JContextExplorer for exploratory analyses of haloarchaeal genomic context evolution. Tree file is in Newick format.
(TXT)

Dataset S12 **Query sequences for osmoadaptation analysis.** Fasta file containing all 74 query sequences used for BLASTp searches for genes potentially involved in osmoadaptation and other ion transport processes.
(FASTA)

Dataset S13 **Query TBP sequences.** Manually curated set of 19 archaeal and eukaryotic sequences used for detection of TATA-binding protein homologs via BLASTp searches.
(FASTA)

Dataset S14 **Query TFB sequences.** Manually curated set of 27 archaeal and eukaryotic sequences used for detection of transcription factor B homologs via BLASTp searches.
(FASTA)

Dataset S15 **Haloarchaeal TFB consensus tree file.** Manually curated alignment of haloarchaeal transcription factor B sequences was resampled 1000 times and trees constructed for each bootstrap replicate using FastTree [104]. The consensus tree was produced with the Consense program in the Phylip package using the extended majority rule option. Each clade in the resulting consensus tree represents the most frequent grouping of those species in the 1000 bootstrap replicate trees, independent of any guide tree. Tree file is in Nexus format.
(TXT)

Dataset S16 **Universal TFB consensus tree file.** Manually curated alignment of archaeal and eukaryotic transcription factor B sequences was resampled 1000 times and trees constructed for each bootstrap replicate using FastTree [104]. The consensus tree was produced with the Consense program in the Phylip package using the extended majority rule option. Each clade in the resulting consensus tree represents the most frequent grouping of those species in the 1000 bootstrap replicate trees, independent of any guide tree. Tree file is in Nexus format.
(TXT)

Dataset S17 **Haloarchaeal TBP consensus tree file.** Manually curated alignment of haloarchaeal TATA-binding protein sequences was resampled 1000 times and trees constructed for each bootstrap replicate using FastTree [104]. The consensus tree was produced with the Consense program in the Phylip package using the extended majority rule option. Each clade in the resulting consensus tree represents the most frequent grouping of those species in the 1000 bootstrap replicate trees, independent of any guide tree. Tree file is in Nexus format.
(TXT)

Dataset S18 **Universal TBP consensus tree file.** Manually curated alignment of archaeal and eukaryotic TATA-binding protein sequences was resampled 1000 times and trees constructed for each bootstrap replicate using FastTree [104]. The consensus tree was produced with the Consense program in the Phylip package using the extended majority rule option. Each clade in the resulting consensus tree represents the most frequent grouping of those species in the 1000 bootstrap replicate trees, independent of any guide tree. Tree file is in Nexus format.
(TXT)

Dataset S19 **Tree file for multi-marker haloarchaeal phylogeny.** A version of the multi-marker archaeal phylogeny (Dataset S3) where non-haloarchaeal species were removed using the R package ape [84]. Tree file is in Newick format.
(TXT)

Text S1 **Miscellaneous. Includes (A–C) instructions for accessing haloarchaeal genomic data through JContextExplorer and SQL database, (D) a list of other haloarchaeal genome sequencing projects, (E) a list of species without annotated photolyases, and (F) a list of species not included in GC bias analysis.**
(DOCX)

Text S2 **BLAST result filtering.** Perl script.
(TXT)

Text S3 **G+C bias calculation and plot generation.** IPython notebook.
(TXT)

Text S4 **Analysis of protein families enriched in abnormal G+C regions.** R code.
(TXT)
