# Supplementary figures and images for: Phylogenetically Driven Sequencing of Extremely Halophilic Archaea Reveals Strategies for Static and Dynamic Osmo-response
Source: PLoS Genet. 2014 Nov 13;10(11):e1004784. doi: 10.1371/journal.pgen.1004784 (PMC4230888; doi:10.1371/journal.pgen.1004784)

# TribeMCL cluster size distribution

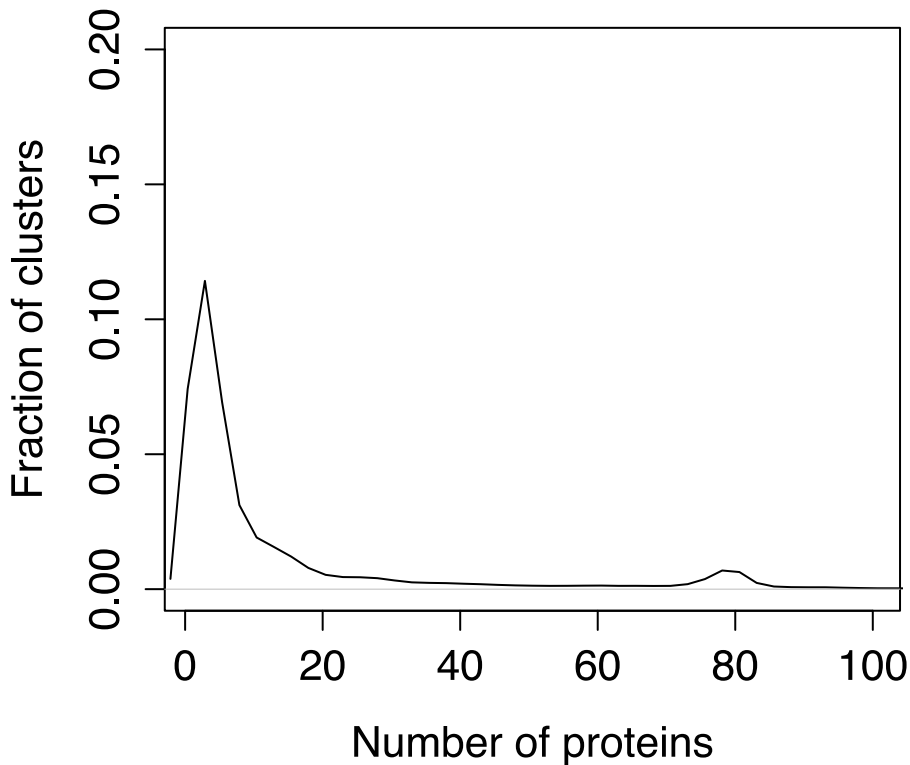

Supplement: Figure S1 — Size distribution of homologous protein families. Density distribution of number of members per homologous protein family (cluster) predicted by TRIBE-MCL (inflation parameter = 2.5). Cluster size distribution is bimodal, with the majority of protein families being very small (≤15 members) and a small group of clusters possessing ∼80 proteins. (PDF) [file pgen.1004784.s001.pdf]

# Number of species per cluster

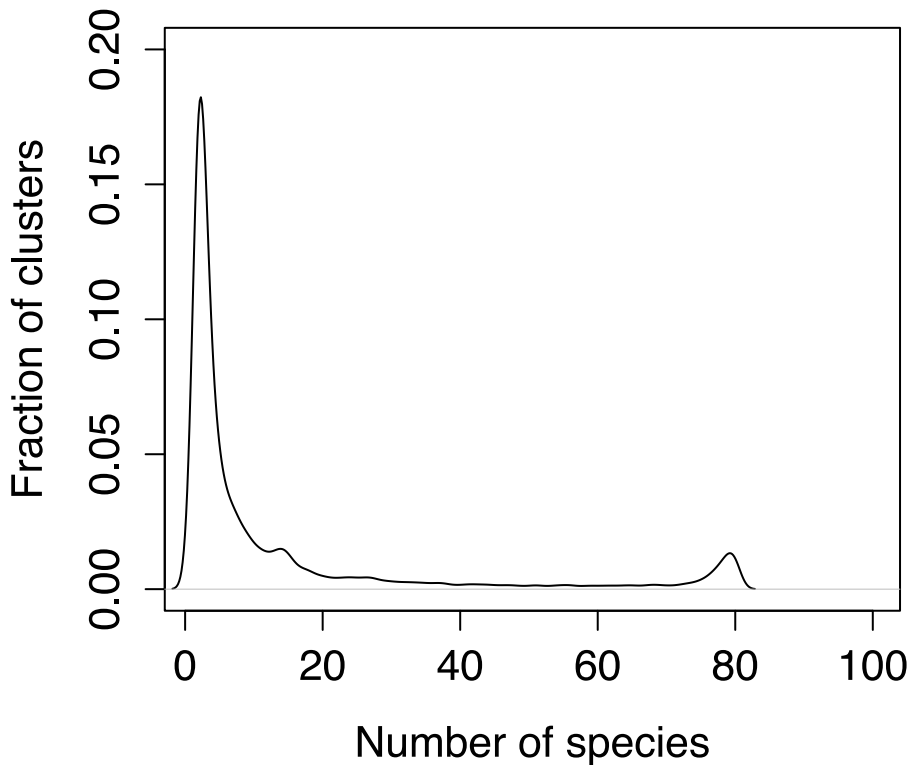

Supplement: Figure S2 — Number of species per homologous protein family. Density distribution of number of species per homologous protein family (cluster) predicted by TRIBE-MCL (inflation parameter = 2.5). Haloarchaeal core protein families are those represented by the minor mode at the right edge of the distribution. (PDF) [file pgen.1004784.s002.pdf]

A

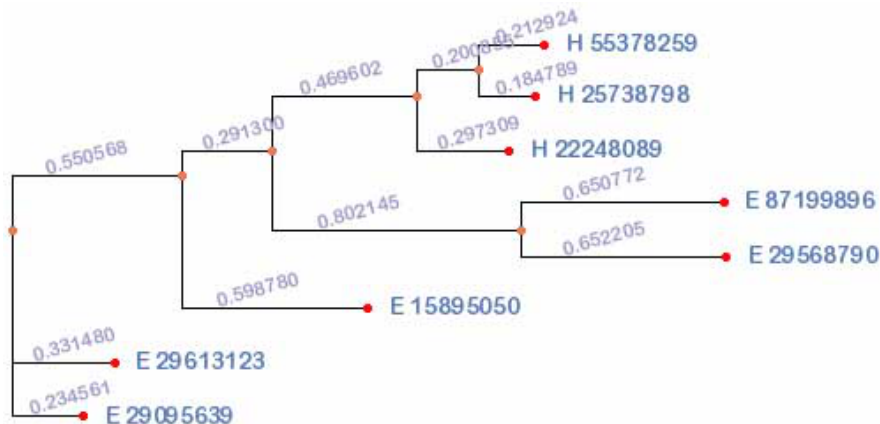

B

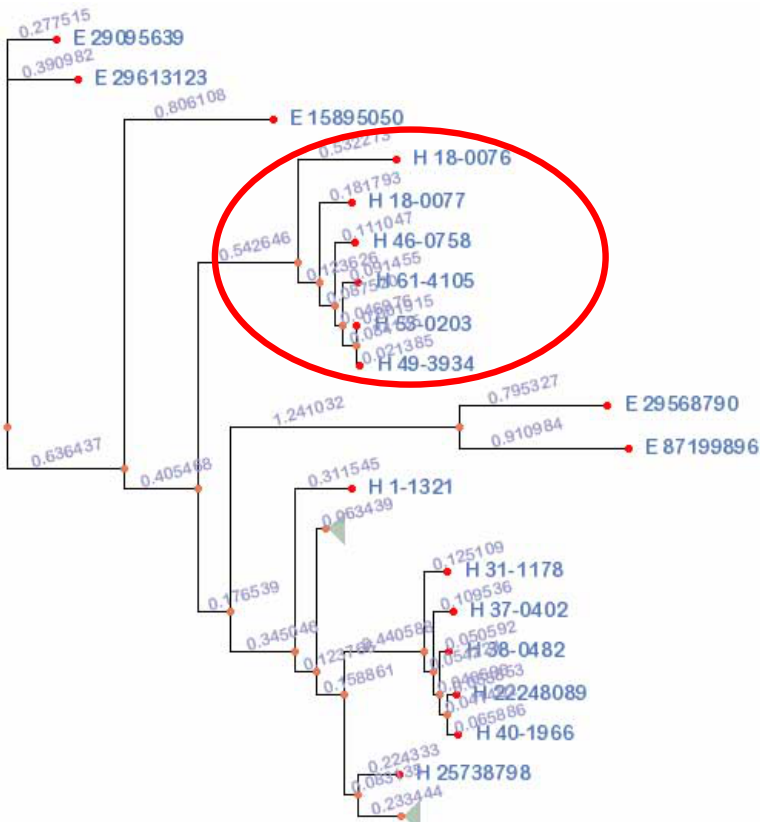

Supplement: Figure S3 — New clade appears in haloarchaea-extended gene tree. The gene tree for protein family 8319 from [25] is consistent with a basal HGT event from bacteria into the haloarchaeal root (A). However, after adding predicted homologs from 65 additional haloarchaeal genomes, the gene tree includes an new group of haloarchaeal homologs not monophyletic with the previously identified group (B, red circle). This is inconsistent with the basal HGT hypothesis, and could indicate an HGT event preceding that identified by [25] for this gene. E = (eu)bacteria, H = haloarchaea. Numerical IDs not containing a dash refer to NCBI gene IDs, those containing a dash refer to proteins from the set of newly-sequenced haloarchaea. Collapsed nodes in (B) contain exclusively haloarchaea. (PDF) [file pgen.1004784.s003.pdf]

A

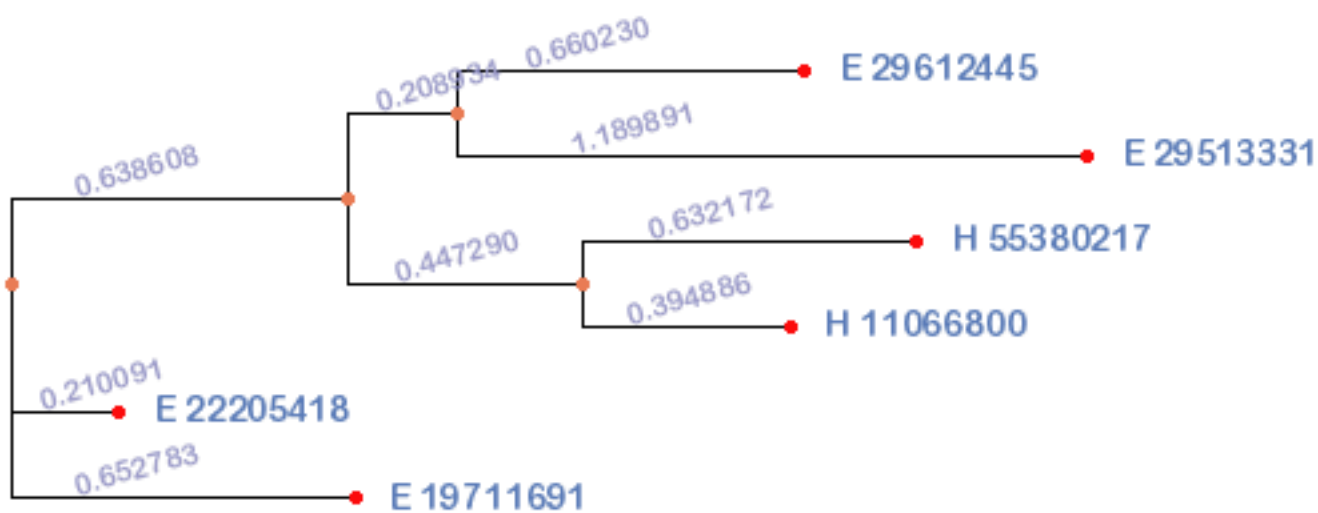

B

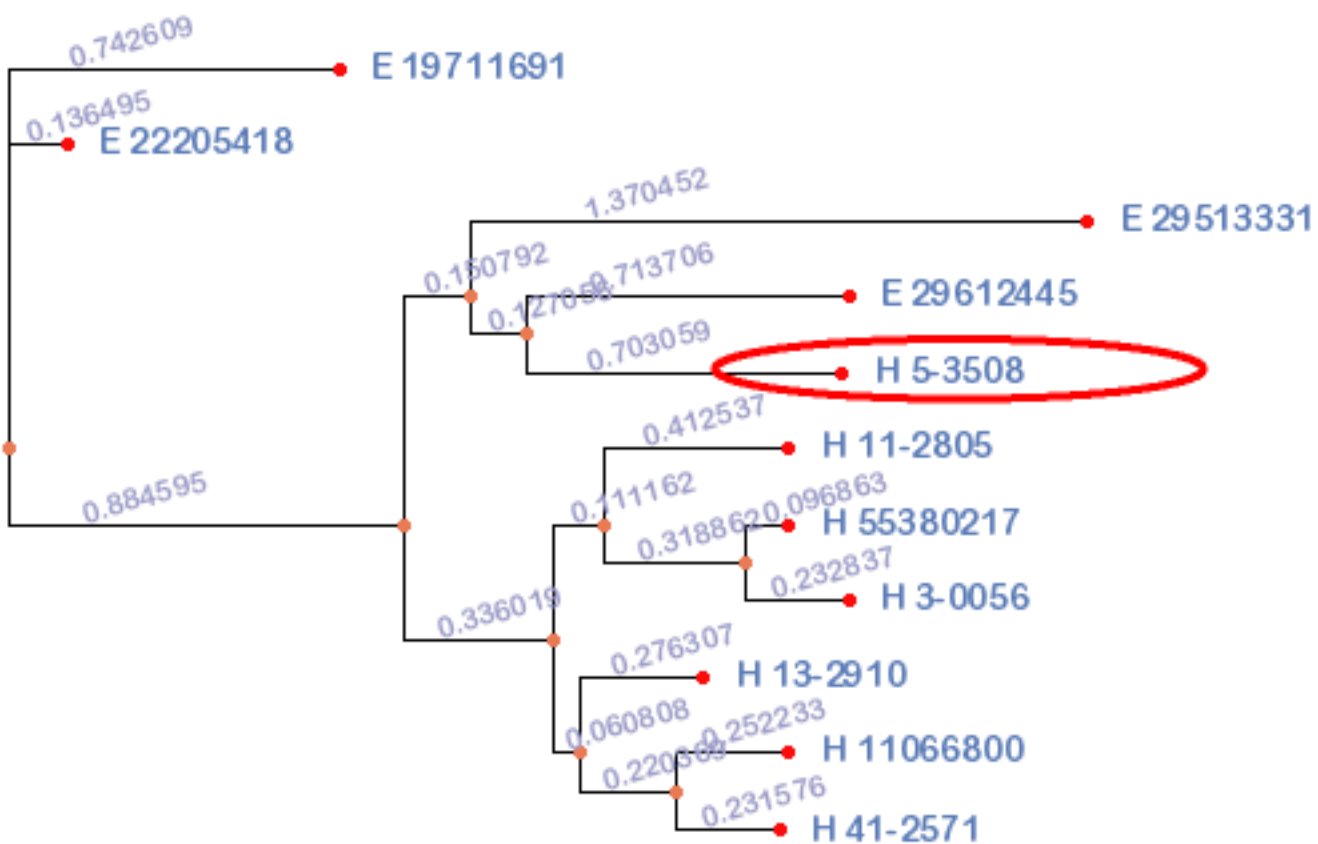

Supplement: Figure S4 — Potential secondary HGT event in haloarchaea-extended gene tree. The gene tree for protein family 12957 from [25] is consistent with a basal HGT event from bacteria into the haloarchaeal root (A). However, after adding predicted homologs from 65 additional haloarchaeal genomes, the gene tree includes a single haloarchaeal homolog which groups with bacterial rather than the remaining haloarchaeal homologs (B, red circle). This is inconsistent with the basal HGT hypothesis, and could indicate a subsequent HGT event, following that identified by [25] for this gene. E = (eu)bacteria, H = haloarchaea. Numerical IDs not containing a dash refer to NCBI gene IDs, those containing a dash refer to proteins from the set of newly-sequenced haloarchaea. (PDF) [file pgen.1004784.s004.pdf]

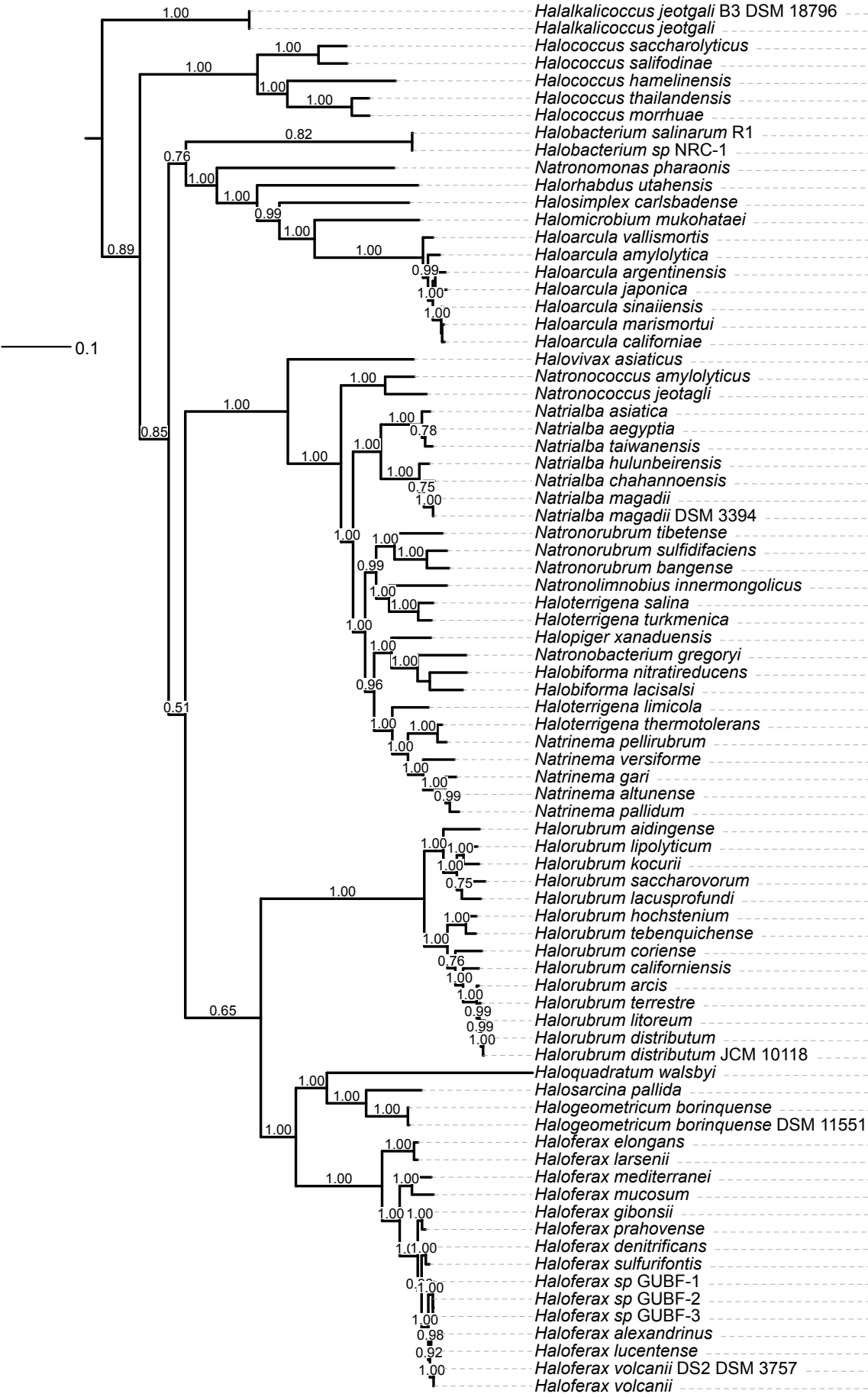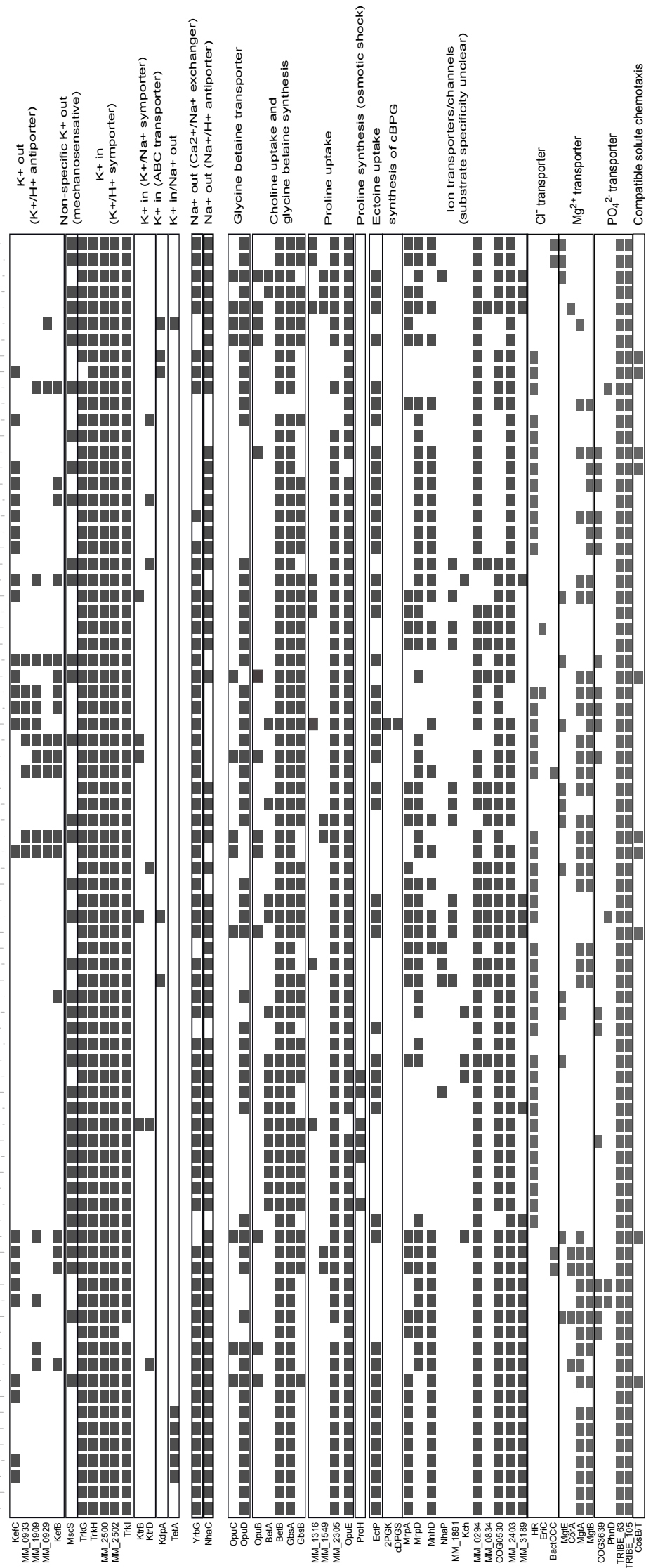

Supplement: Figure S5 — Phylogenetic distribution of genes involved in ion transport, and compatible solute transport and biosynthesis. Presence/absence pattern of 54 genes involved in ion transport, compatible solute transport or compatible solute biosynthesis. Homologs were detected by BLAST searches, filtered according to metrics given in Materials and Methods, and presence/absence data superimposed on haloarchaeal phylogenetic tree (Dataset S11). Gray squares represent presence of a particular homolog. Homologs are grouped by functional category - listed across the top of the figure. Each column represents one homolog – listed along the bottom of the figure. Tree roots in the Methanocella (not shown). Genes in search set with no haloarchaeal homologs are not shown (see Datasets S4 & S12). (PDF) [file pgen.1004784.s005.pdf]

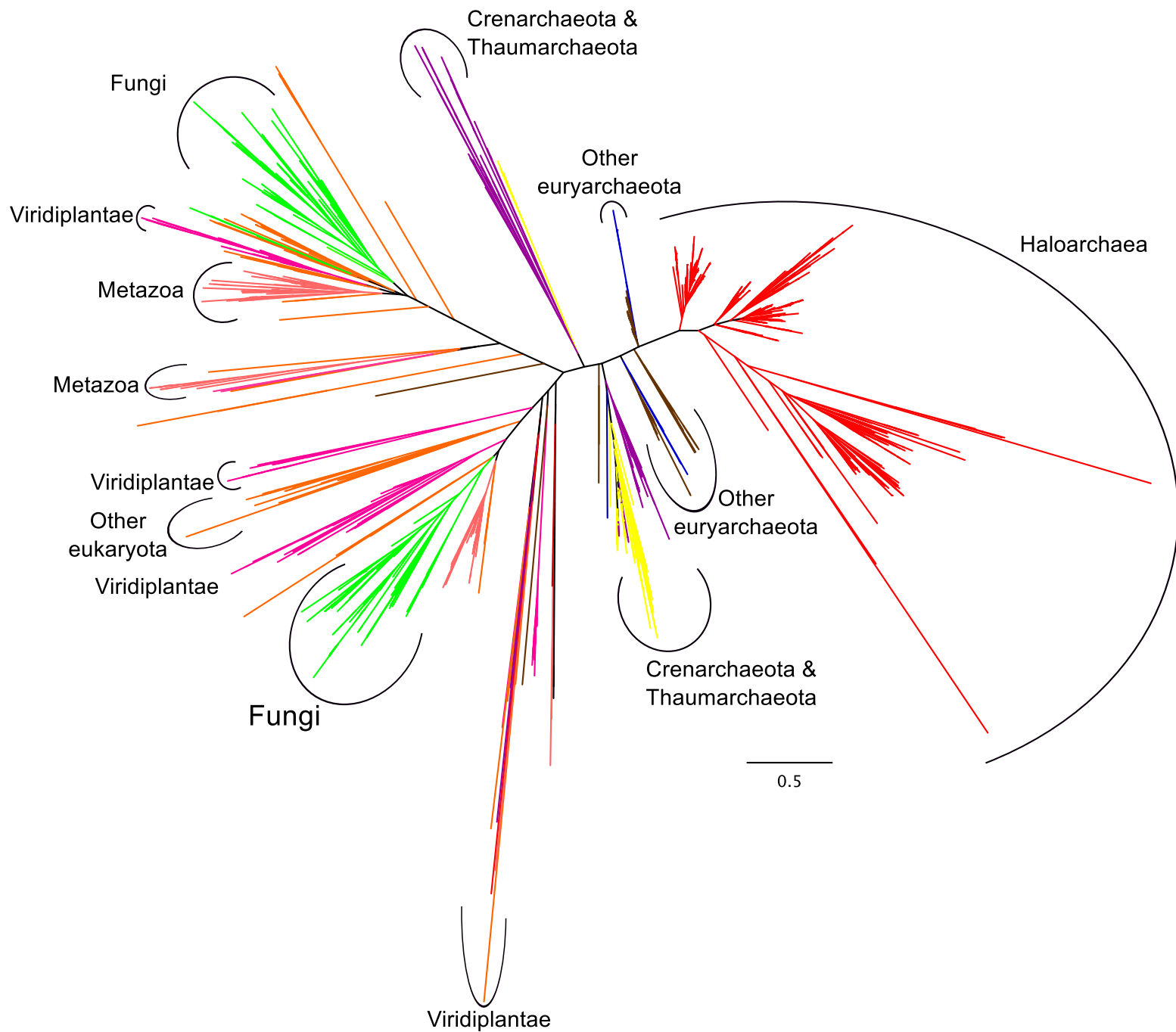

Supplement: Figure S9 — Universal transcription factor B phylogeny. Unrooted phylogenetic tree of transcription factor B (TFB) homologs from NCBI's non-redundant protein database and recently sequenced haloarchaeal genomes. Red - haloarchaea, brown - other euryarchaeota, bright purple - crenarchaeota, yellow - thaumarchaeota, dark blue - other/unclassified archaea, salmon - metazoa, bright green - fungi, hot pink - viridiplantae, orange - other eukaryotes. See Datasets S6 for tree file. (PDF) [file pgen.1004784.s009.pdf]

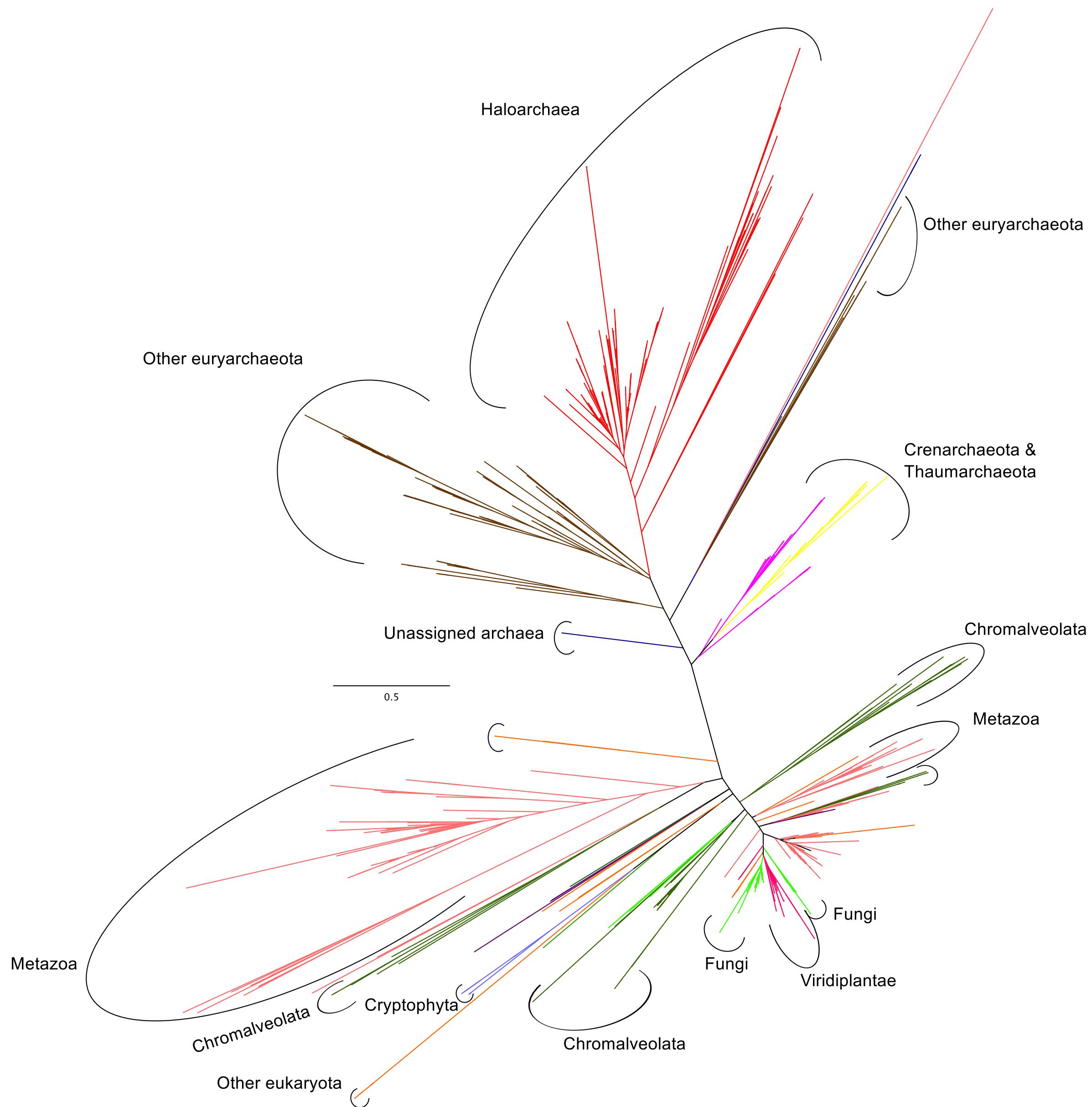

Supplement: Figure S10 — Universal TATA-binding protein phylogeny. Unrooted phylogenetic tree of TATA-binding protein (TBP) homologs from NCBI's non-redundant protein database and recently sequenced haloarchaeal genomes. Red - haloarchaea, brown - other euryarchaeota, bright purple - crenarchaeota, yellow - thaumarchaeota, dark blue - other/unclassified archaea, dark green - chromalveolata, salmon - metazoa, dark purple - cryptophyta, lavender - excavata, bright green - fungi, hot pink - viridiplantae, orange - other eukaryotes. See Dataset S7 for tree file. (PDF) [file pgen.1004784.s010.pdf]

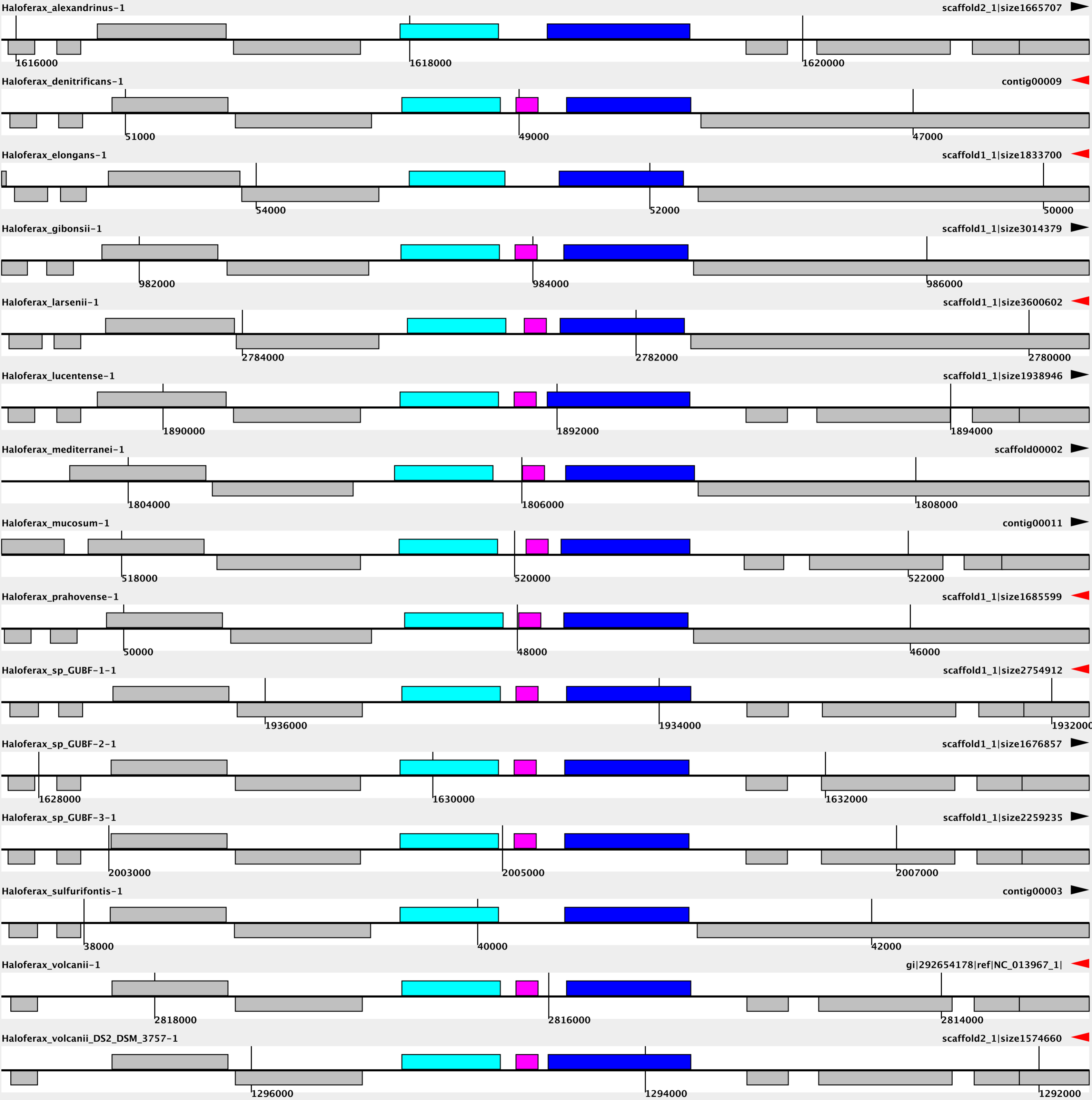

Supplement: Figure S11 — Genomic regions in Haloferax suggestive of a missed gene call. Analysis of genomic neighborhoods for 15 Haloferax species revealed a small hypothetical protein (Tribe4688, magenta) between a hypothetical protein (Tribe966, light blue) and a protein annotated as co-occurring with transport systems (Tribe458, dark blue), in most species. However this gene was not called in three species. The high degree of genomic context conservation suggests that the RAST gene caller may have missed a copy of Tribe4688 in these species. Species name is shown in the upper left of each segment. Black arrow = forward strand, red arrow = reverse strand. Grey boxes represent genes not involved in this analysis. (PDF) [file pgen.1004784.s011.pdf]

A

B

C

D

E

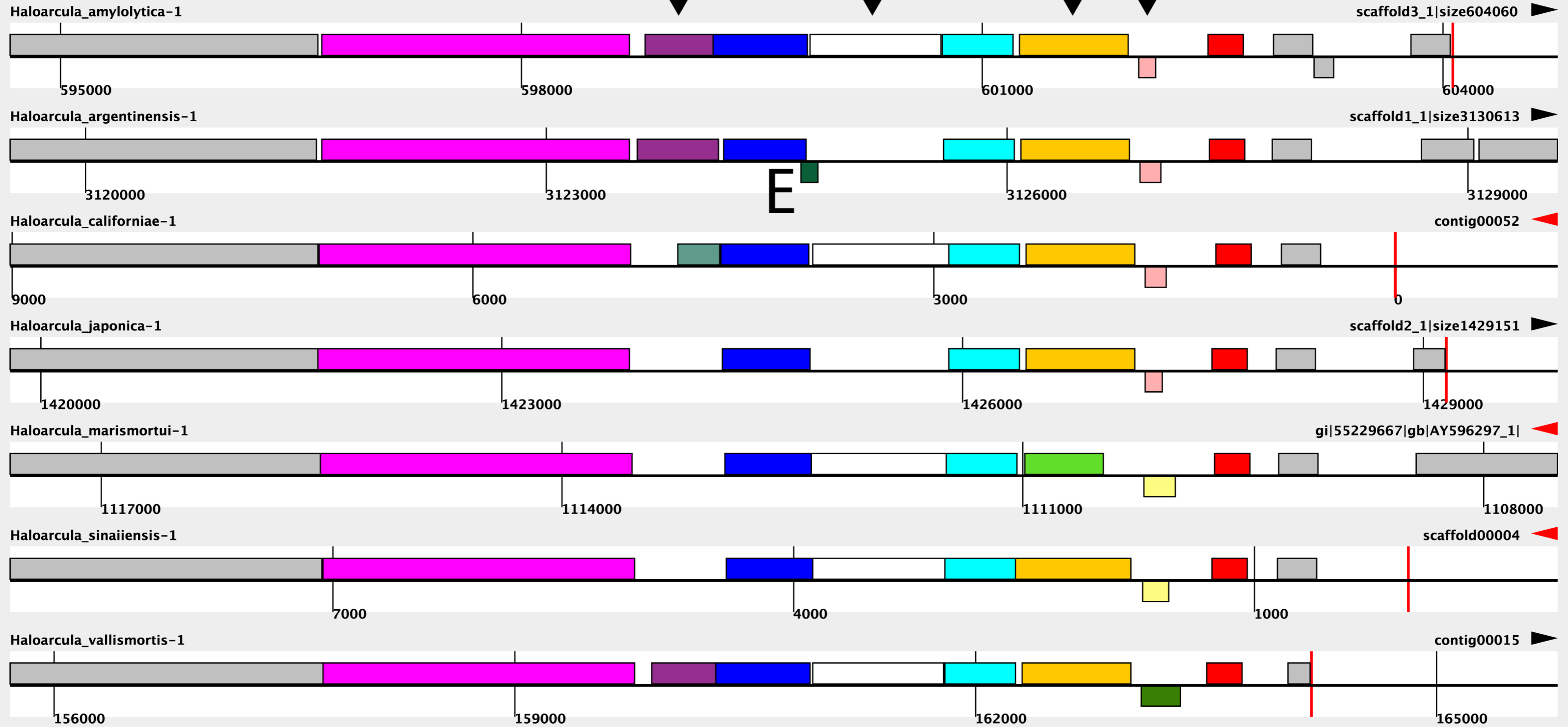

Supplement: Figure S13 — A region of poor gene call consistency in Haloarcula spp. Genomic regions are shown between Tribe254 and Tribe369 when these genes fall within 5 Kb. Only four of the intervening genes are consistent across all six Haloarcula species (245, magenta; 5724, dark blue; 6141, cyan; and 369, red). Tribe7403 (white) may be an example of a missed gene call (absent in two species) (B). This may also be the case for Tribe9660 (purple)/Tribe18431 (grayish-blue), which is missing in three species and apparently truncated in one (A). Tribes 6775 (orange), 18947 (lime green), 8276 (pink), 12986 (light yellow), and 19374 (green) may be cases where translation start and stop sites have not been correctly called (C and D). Finally, the short, hypothetical protein Tribe18194 (dark green) may be a spurious gene call (E). This region serves as an example to highlight the necessity for improvements in automated gene call prediction. (PDF) [file pgen.1004784.s013.pdf]

(A)

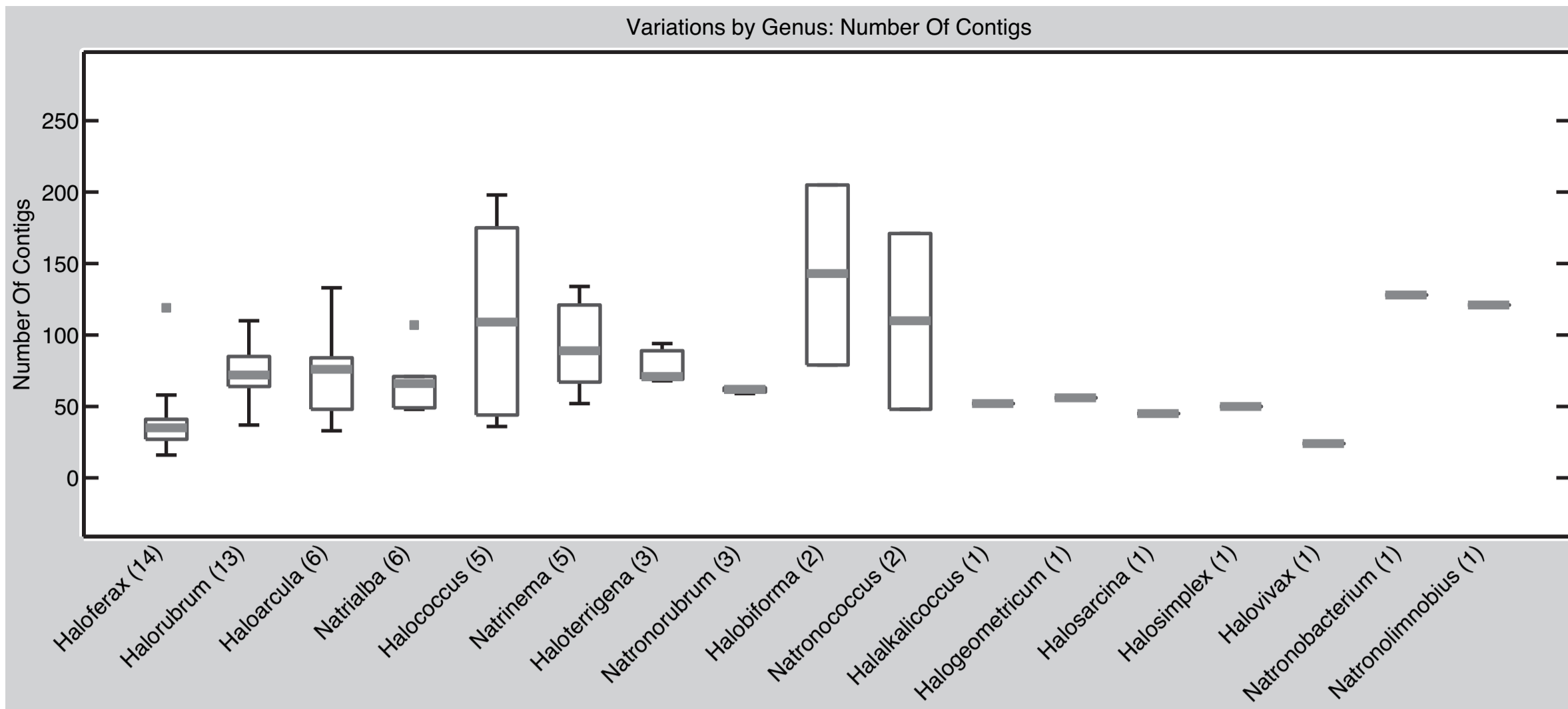

(B)

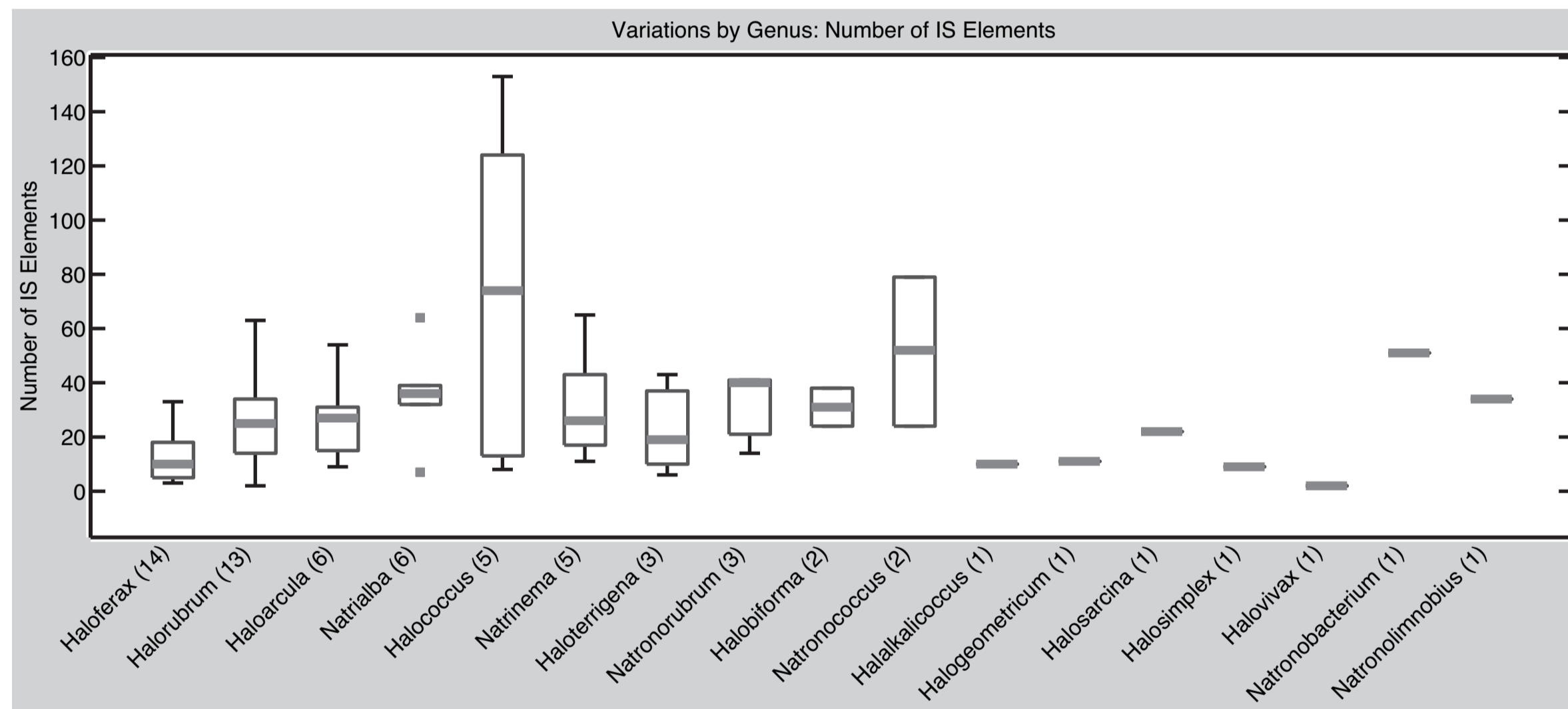

(C)

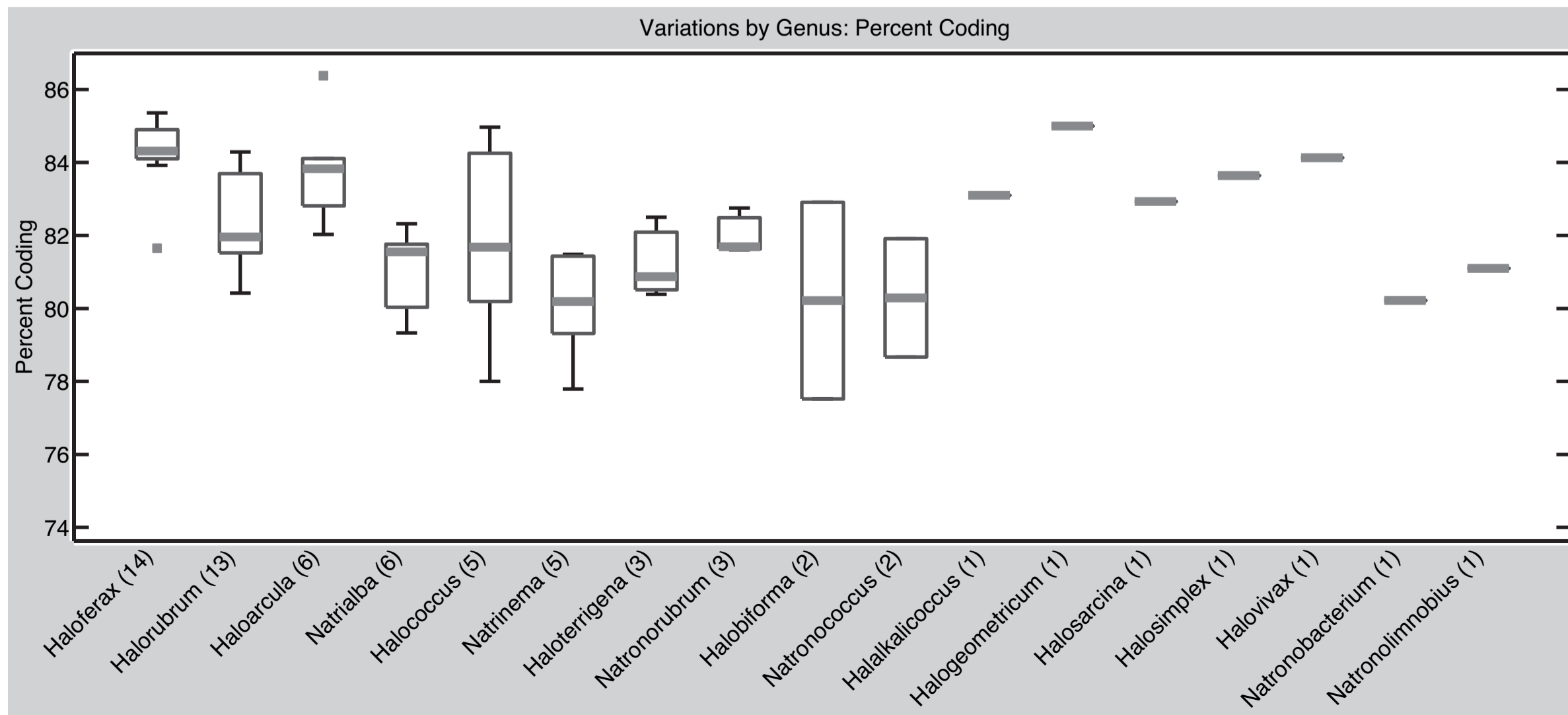

Supplement: Figure S23 — Genera-level comparisons of genomic features (part II). Number of contigs (A), number of insertion elements (B) and percent coding DNA (C) were extracted from each genome, organized by genus, and boxplots calculated using MATLAB's Statistics toolbox [93]. Boxplots were generated using 25th and 75th percentile as box edges, with median demarcated with horizontal line within box. Genera are ordered by descending number of species sequenced, with the number of species shown in parentheses. Genera with only a single sequenced member are shown as horizontal lines. (PDF) [file pgen.1004784.s023.pdf]
