# Supplementary material for: Phylogenetically Driven Sequencing of Extremely Halophilic Archaea Reveals Strategies for Static and Dynamic Osmo-response
Source: PLoS Genet. 2014 Nov 13;10(11):e1004784. doi: 10.1371/journal.pgen.1004784 (PMC4230888; doi:10.1371/journal.pgen.1004784)
Supplement: Figure S6 — Pattern of TATA-binding protein acidification. Comparative acidification of TATA-binding protein (TBP) binding to DNA for three non-halophilic archaea (left, black text) and two haloarchaea (right, red text). Acidified regions are shown in red, while basic regions are blue. Close-up of DNA-binding site reveals that these regions remain unacidified in the haloarchaea. The opposite side of the TBP protein (bottom), which does not interact directly with the DNA, demonstrates significant acidification in haloarchaea. A. fulgidus = Archaeoglobus fulgidus, M. hungatei = Methanospirillum hungatei, M. paludicola = Methanocella paludicola, Nmn. = Natronomonas, Har. = Haloarcula. Structural model used was that for Pyrococcus woesei (ID3U) [100]. (PDF) [file pgen.1004784.s006.pdf]

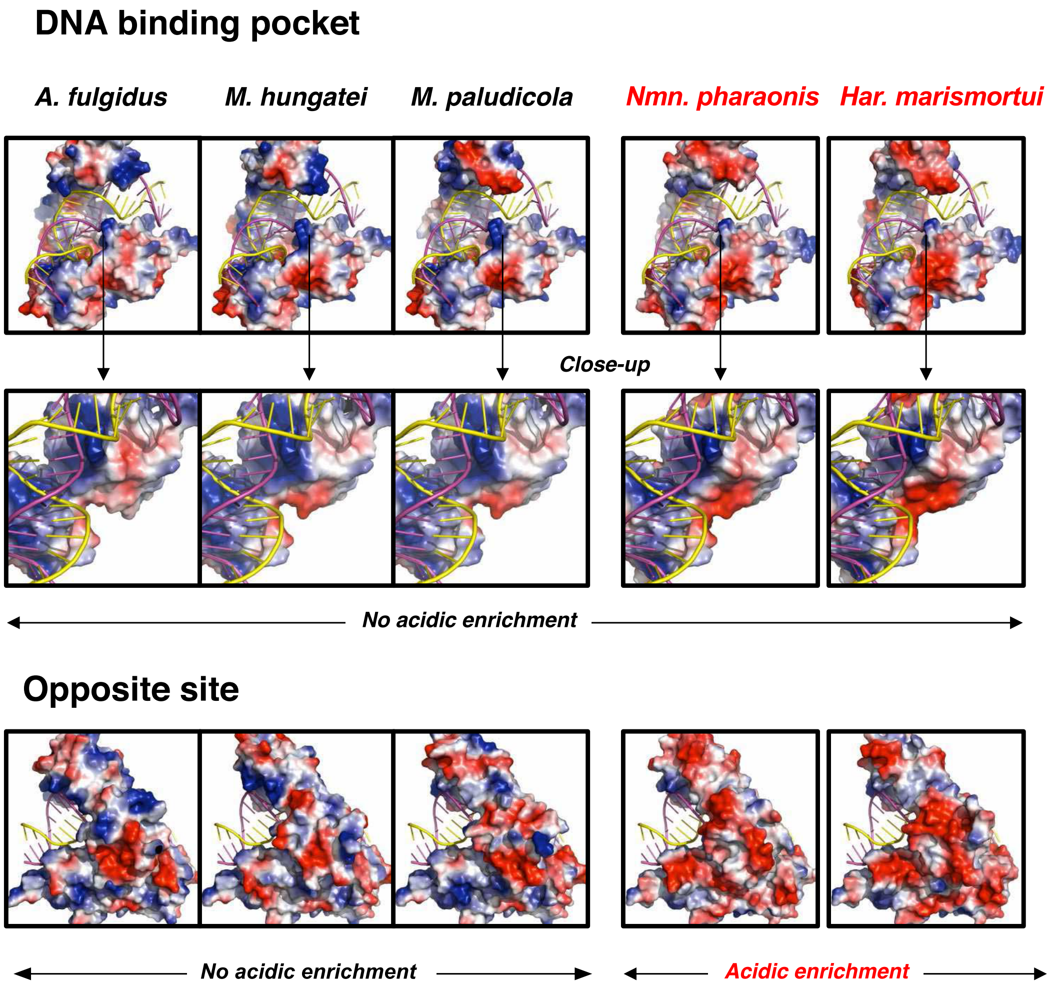
DNA binding pocket
A. fulgidus
M. hungatei
M. paludicola
Nmn. pharaonis
Har. marismortui
Close-up
No acidic enrichment
Opposite site
No acidic enrichment
Acidic enrichment
