# Supplementary material for: Phylogenetically Driven Sequencing of Extremely Halophilic Archaea Reveals Strategies for Static and Dynamic Osmo-response
Source: PLoS Genet. 2014 Nov 13;10(11):e1004784. doi: 10.1371/journal.pgen.1004784 (PMC4230888; doi:10.1371/journal.pgen.1004784)
Supplement: Figure S7 — Pattern of ribosome elongation factor α-1 acidification. Comparative acidification of tRNA-binding versus non-tRNA-binding side of ribosomal elongation factor α-1 for five non-halophilic archaea (left) and three haloarchaea (right). Acidified regions are shown in red, while basic regions are blue. No acidic enrichment was detected for the tRNA-binding face (top), while acidic enrichment was apparent in solvent-exposed face of haloarchaeal homologs (bottom). P. torridus = Picrophilus torridus, A. fulgidus = Archaeoglobus fulgidus, M. maripaludis = Methanococcus maripaludis, M. acetivorans = Methanosarcina acetivorans, M. mazei = Methanosarcina mazei, Hqt. = Haloquadratum, Hbt. = Halobacterium, Nmn. = Natronomonas. Structural model used was that for Aeropyrum pernix K1 (3VMF) [101]. (PDF) [file pgen.1004784.s007.pdf]

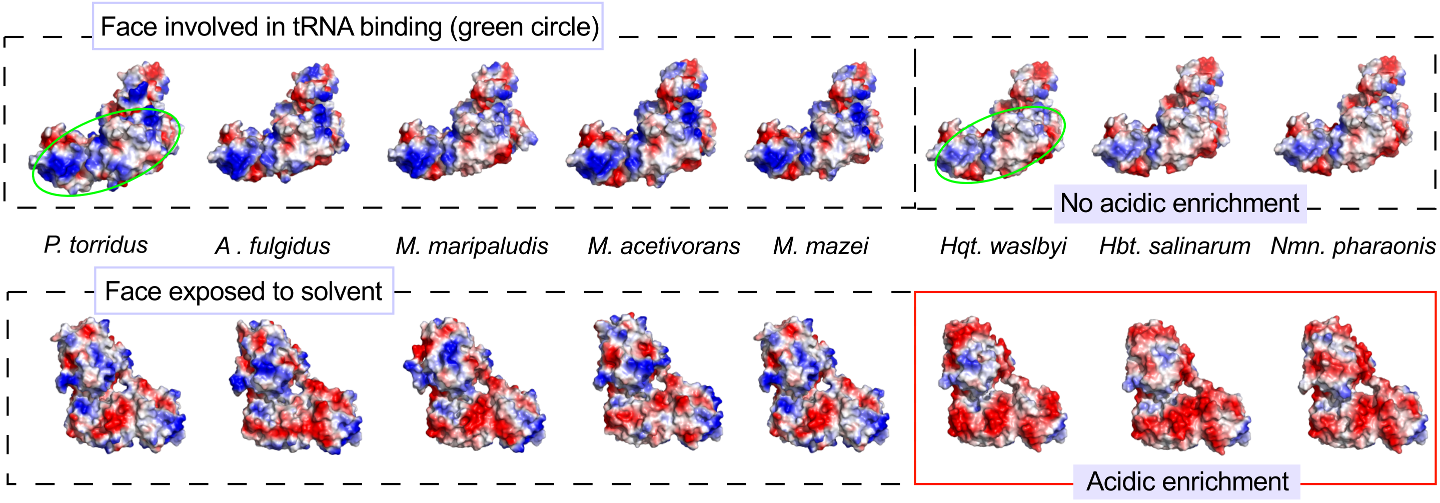

Face involved in tRNA binding (green circle)
No acidic enrichment
P. torridus
A . fulgidus
M. maripaludis
M. acetivorans
M. mazei
Hqt. waslbyi
Hbt. salinarum
Nmn. pharaonis
Face exposed to solvent
Acidic enrichment
