# Supplementary material for: Phylogenetically Driven Sequencing of Extremely Halophilic Archaea Reveals Strategies for Static and Dynamic Osmo-response
Source: PLoS Genet. 2014 Nov 13;10(11):e1004784. doi: 10.1371/journal.pgen.1004784 (PMC4230888; doi:10.1371/journal.pgen.1004784)
Supplement: Figure S8 — Representative selected changepoints for G+C content analysis. Representative G+C content plots for (A) Haloferax mediterranei, (B) Halorubrum litoreum, (C) Haloarcula argentinensis, and (D) Natrialba aegyptia showing manually curated changepoints. Each of these four species is a representative from the four most populous genera in the set, which collectively contain 66% of the organisms in the study. Black line represents calculated G+C percent for each 100 bp window and vertical green lines represent points of inflection in the mean G+C percent which were selected as changepoints. Genomic regions between changepoints were taken as regions of abnormal G+C content, extracted and analyzed for enrichment of functional groups. The horizontal axis displays the number of 20 bp steps taken along the genome. Representative species selected correspond to those shown in Figure 7. (PDF) [file pgen.1004784.s008.pdf]

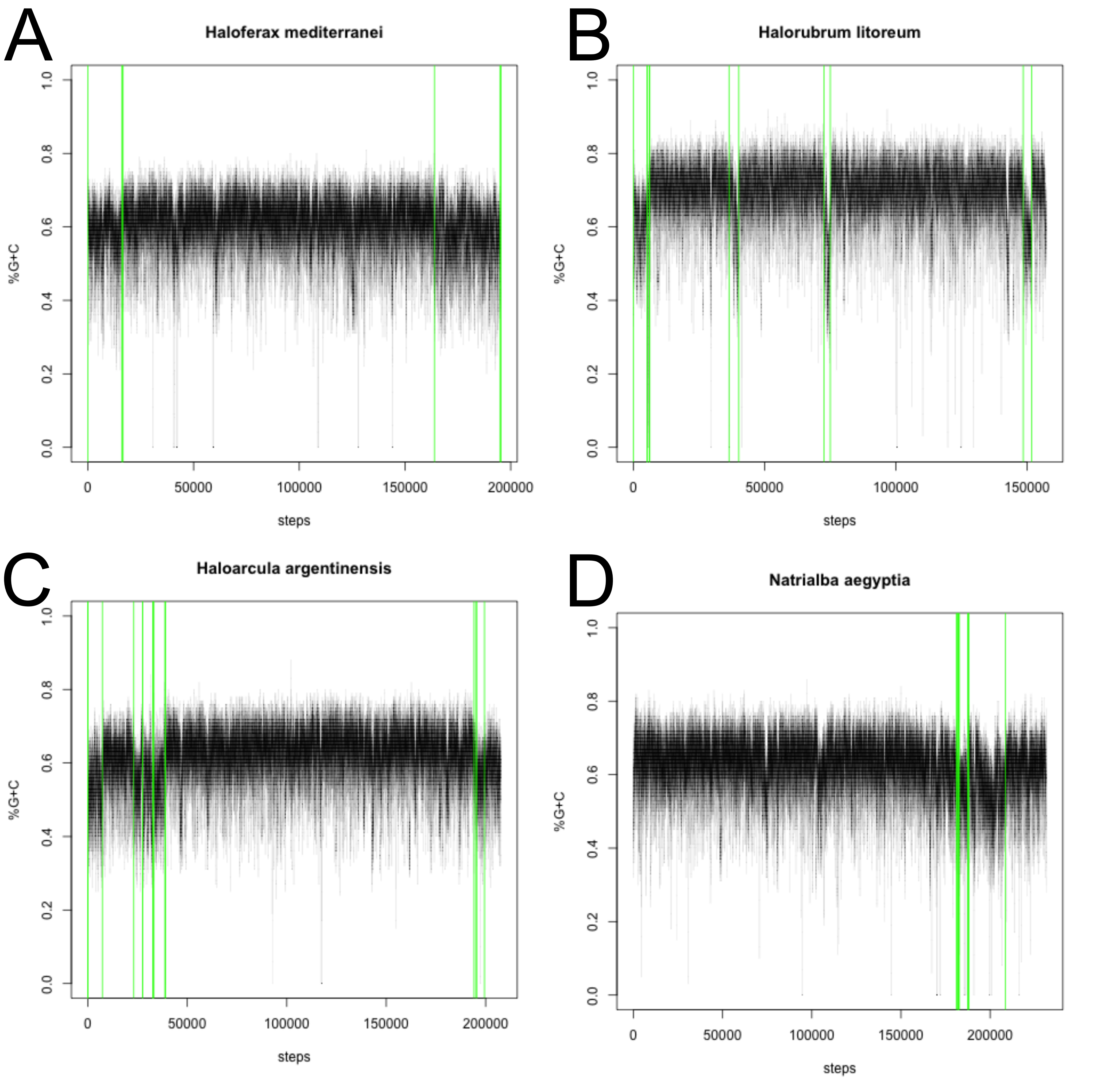
A
Haloferax mediterranei
B
Halorubrum litoreum
C
Haloarcula argentinensis
D
Natrialba aegyptia
%G+C
steps
0.0
0.2
0.4
0.6
0.8
1.0
0
50000
100000
150000
200000
