# Supplementary material for: Phylogenetically Driven Sequencing of Extremely Halophilic Archaea Reveals Strategies for Static and Dynamic Osmo-response
Source: PLoS Genet. 2014 Nov 13;10(11):e1004784. doi: 10.1371/journal.pgen.1004784 (PMC4230888; doi:10.1371/journal.pgen.1004784)
Supplement: Figure S12 — Alignments of Tribe4688 with predicted missed gene calls. The complete protein sequence is shown for every instance of Tribe4688 in the Haloferax, with three additional protein sequences identified in the gap regions where a protein was expected to be found. The homologs sorted into two groups, with the three proposed missing genes (red boxes) sorting among these groups, suggesting that gene caller misses were not based on sequence conservation thresholds. The high degree of sequence conservation and match in length strongly suggests that the observed absence of this gene in Hfx. alexandrinus, sulfurifontis, and elongans is the result of gene caller errors rather than a biological phenomenon. (PDF) [file pgen.1004784.s012.pdf]

```
pred  Haloferax alexandrinus    MVSMRSCMCCGEPISETRHLCGVCIQNGCTSYADACGR
4688  Haloferax denitrificans   MGSMRSCMCCGEPISETRHLCGVCIQNGCTSYADACGQ
4688  Haloferax gibonsii        MVSMRSCMCCGEPISETRHLCGVCIQNGCTSYADACGQ
4688  Haloferax lucentense      MVSMRSCMCCGEPISETRHLCGVCIQNGCTSYADACGQ
4688  Haloferax prahovense      MVSMRSCMCCGEPISETRHLCGVCIQNGCTSYADACGQ
4688  Haloferax sp_GUBF-1       MVSMRSCMCCGEPISETRHLCGVCIQNGCTSYADACGQ
4688  Haloferax sp_GUBF-2       MVSMRSCMCCGEPISETRHLCGVCIQNGCTSYADACGQ
4688  Haloferax sp_GUBF-3       MVSMRSCMCCGEPISETRHLCGVCIQNGCTSYADACGQ
pred  Haloferax sulfurifontis   MGSMRSCMCCGEPISETRHLCGVCIQNGCTSYADACGQ
4688  Haloferax volcanii_DS2    MVSMRSCMCCGEPISETRHLCGVCIQNGCTSYADACGQ
4688  Haloferax volcanii        MVSMRSCMCCGEPISETRHLCGVCIQNGCTSYADACGQ

pred  Haloferax elongans        MGELRTCLCCGAPVSKTRHLCGVCVQNGCTSYANACGQ
4688  Haloferax larsenii        MDESNTCLCCGAPVSKTRHLCGVCVQNGCTSYANVCGQ
4688  Haloferax mediterranei    MERPRSCMCCGAQISETRHLCGVCIQNGCTSYANICGQ
4688  Haloferax mucosum         MTRTRACMCCGATISDVRHLCGVCIQNGCTSYANTCGQ
                                    .:*:***  :*..*******:********: **:
```
