# Supplementary material for: Phylogenetically Driven Sequencing of Extremely Halophilic Archaea Reveals Strategies for Static and Dynamic Osmo-response
Source: PLoS Genet. 2014 Nov 13;10(11):e1004784. doi: 10.1371/journal.pgen.1004784 (PMC4230888; doi:10.1371/journal.pgen.1004784)
Supplement: Figure S15 — Phylogenetic profiling assisted gene annotation (cobalamin metabolism). Phylogenetic distribution patterns of unannotated genes assist with prediction of gene function. Cases where unannotated genes have similar phylogenetic distribution to a number of genes with predicted functions allow for hypotheses to be made about the functions of unannotated group members. Visualization and hierarchical clustering of protein presence and absence data was done using Mev [108]. Black represents absence and red represents presence of a protein family. Consense annotations and numbers corresponding to TRIBE-MCL protein families are shown on the right. (PDF) [file pgen.1004784.s015.pdf]

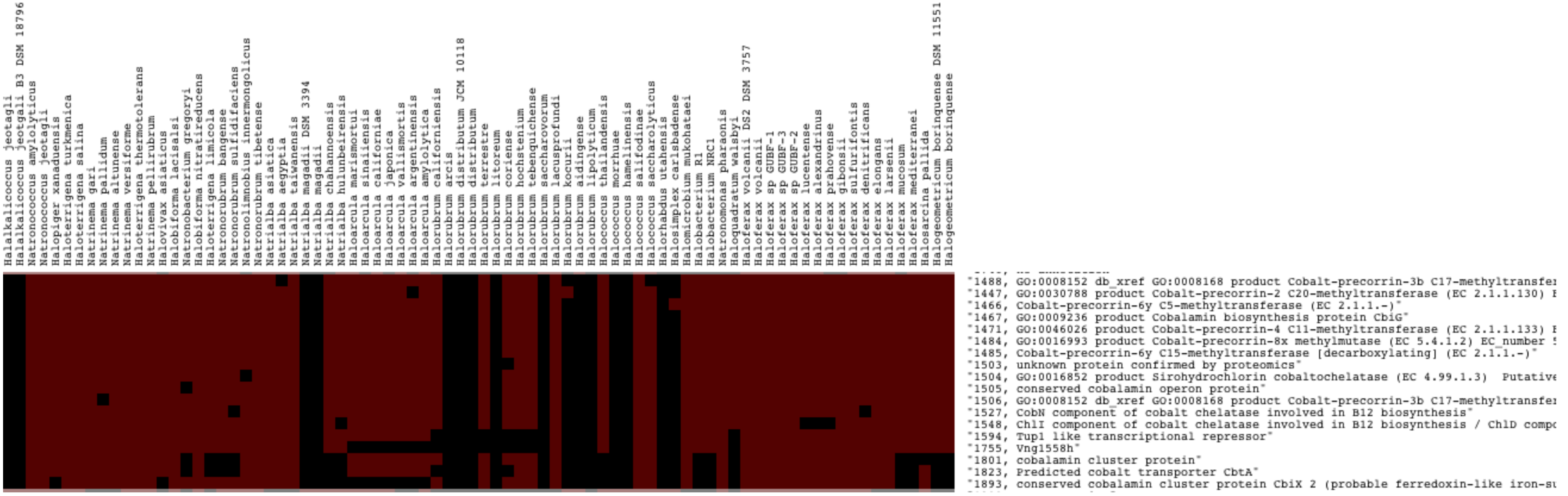
Halalkalicoccus jeotagli
Halalkalicoccus jeotgali B3 DSM 18796
Natronococcus amylolyticus
Natronococcus jeotgali
Halopiger xanaduensis
Haloterrigena turkmenica
Haloterrigena salina
Natrinema gari
Natrinema pallidum
Natrinema altunense
Natrinema versiforme
Haloterrigena thermotolerans
Natrinema pellirubrum
Halovivax asiaticus
Halobiforma lacisalsi
Natronobacterium gregoryi
Halobiforma nitratireducens
Haloterrigena limicola
Natronorubrum bangense
Natronorubrum sulfidifaciens
Natronolimnobius innermongolicus
Natronorubrum tibetense
Natrialba asiatica
Natrialba aegyptia
Natrialba taiwanensis
Natrialba magadii DSM 3394
Natrialba magadii
Natrialba chahannaoensis
Natrialba hulunbeirensis
Haloarcula marismortui
Haloarcula sinaiiensis
Haloarcula californiae
Haloarcula japonica
Haloarcula vallismortis
Haloarcula argentinensis
Haloarcula amylolytica
Halorubrum californiensis
Halorubrum arcis
Halorubrum distributum JCM 10118
Halorubrum distributum
Halorubrum terrestre
Halorubrum litoreum
Halorubrum coriense
Halorubrum hochstenium
Halorubrum tebenquichense
Halorubrum saccharovorum
Halorubrum lacusprofundi
Halorubrum kocurii
Halorubrum aidingense
Halorubrum lipolyticum
Halococcus thailandensis
Halococcus morrhuae
Halococcus hamelinensis
Halococcus salifodinae
Halococcus saccharolyticus
Halorhabdus utahensis
Halosimplex carlsbadense
Halomicrobium mukohataei
Halobacterium R1
Halobacterium NRC1
Natronomonas pharaonis
Haloquadratum walsbyi
Haloferax volcanii DS2 DSM 3757
Haloferax volcanii
Haloferax sp GUBF-1
Haloferax sp GUBF-3
Haloferax sp GUBF-2
Haloferax lucentense
Haloferax alexandrinus
Haloferax prahovense
Haloferax gibonsii
Haloferax sulfurifontis
Haloferax denitrificans
Haloferax elongans
Haloferax larsenii
Haloferax mucosum
Haloferax mediterranei
Halosarcina pallida
Halogeometricum boringuense DSM 11551
Halogeometricum boringuense
"1488, GO:0008152 db_xref GO:0008168 product Cobalt-precorrin-3b C17-methyltransfe
"1447, GO:0030788 product Cobalt-precorrin-2 C20-methyltransferase (EC 2.1.1.130)
"1466, Cobalt-precorrin-6y C5-methyltransferase (EC 2.1.1.-)"
"1467, GO:0009236 product Cobalamin biosynthesis protein CbiG"
"1471, GO:0046026 product Cobalt-precorrin-4 C11-methyltransferase (EC 2.1.1.133)
"1484, GO:0016993 product Cobalt-precorrin-8x methylmutase (EC 5.4.1.2) EC_number
"1485, Cobalt-precorrin-6y C15-methyltransferase [decarboxylating] (EC 2.1.1.-)"
"1503, unknown protein confirmed by proteomics"
"1504, GO:0016852 product Sirohydrochlorin cobaltochelatase (EC 4.99.1.3) Putative
"1505, conserved cobalamin operon protein"
"1506, GO:0008152 db_xref GO:0008168 product Cobalt-precorrin-3b C17-methyltransfe
"1527, CobN component of cobalt chelatase involved in B12 biosynthesis"
"1548, ChlI component of cobalt chelatase involved in B12 biosynthesis / ChlD comp
"1594, Tup1 like transcriptional repressor"
"1755, Vng1558h"
"1801, cobalamin cluster protein"
"1823, Predicted cobalt transporter CbtA"
"1893, conserved cobalamin cluster protein CbiX 2 (probable ferredoxin-like iron-s
