# Supplementary material for: Phylogenetically Driven Sequencing of Extremely Halophilic Archaea Reveals Strategies for Static and Dynamic Osmo-response
Source: PLoS Genet. 2014 Nov 13;10(11):e1004784. doi: 10.1371/journal.pgen.1004784 (PMC4230888; doi:10.1371/journal.pgen.1004784)
Supplement: Text S1 — Miscellaneous. Includes (A–C) instructions for accessing haloarchaeal genomic data through JContextExplorer and SQL database, (D) a list of other haloarchaeal genome sequencing projects, (E) a list of species without annotated photolyases, and (F) a list of species not included in GC bias analysis. (DOCX) [file pgen.1004784.s049.docx]

1. **Accessing haloarchaeal genomic data through JContextExplorer.**
2. Navigate to the Facciotti Web site, software page.

<http://www.bme.ucdavis.edu/facciotti/resources_data/software/>

1. Launch JContextExplorer, via the directions described on the software page.
2. Once in the program, in the menu bar, navigate to Genomes -> Retrieve Popular Genome Set -> Haloarchaea


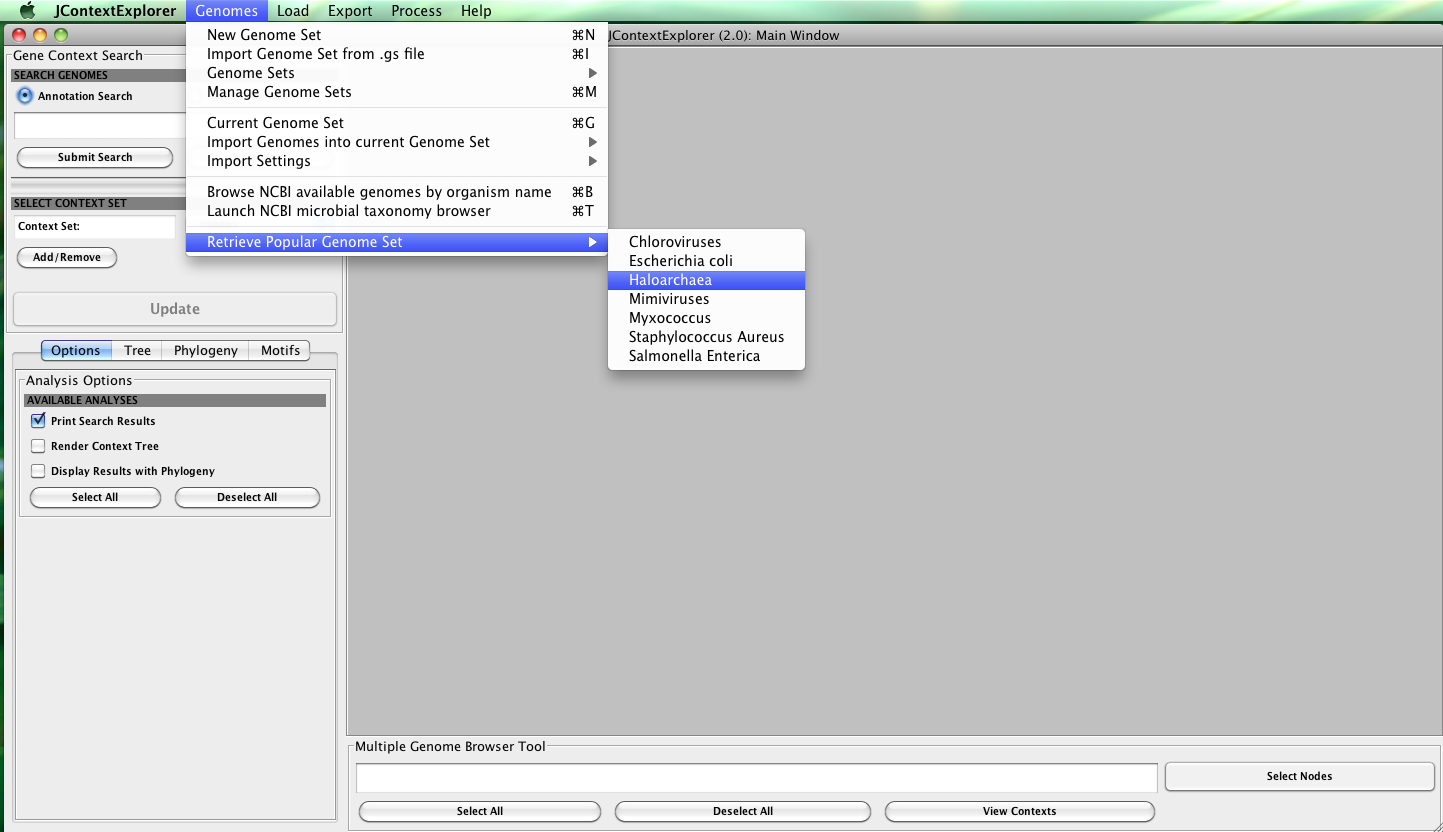


1. If this fails, you may also download the Haloarchaea genome set as a .gs file, which is available on the Facciotti lab software page given in step 1. Once this file has been downloaded and unzipped, the haloarchaea genome set may be imported by navigating to Genomes -> Import Genome Set from .gs file, and selecting the unzipped .gs file


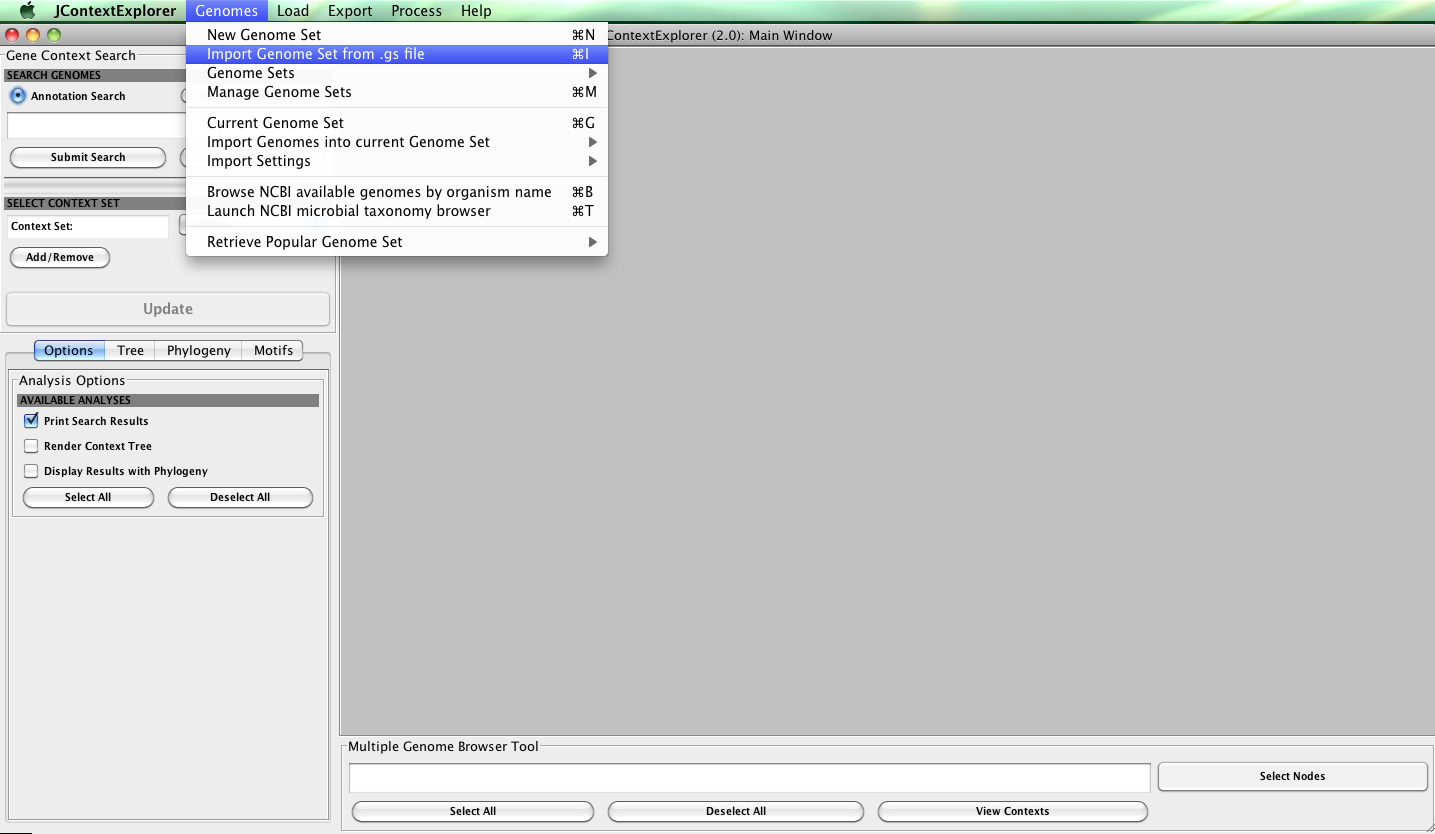


1. **Basic Information retrieval from JContextExplorer**

**(1) Extended instructions**

This short tutorial refers only how to retrieve basic information

from JContextExplorer, and does not explain how to use

many of JContextExplorer’s powerful features. A complete

user’s manual and several video tutorials are accessible directly through

the software, however, by navigating to the Help Menu:


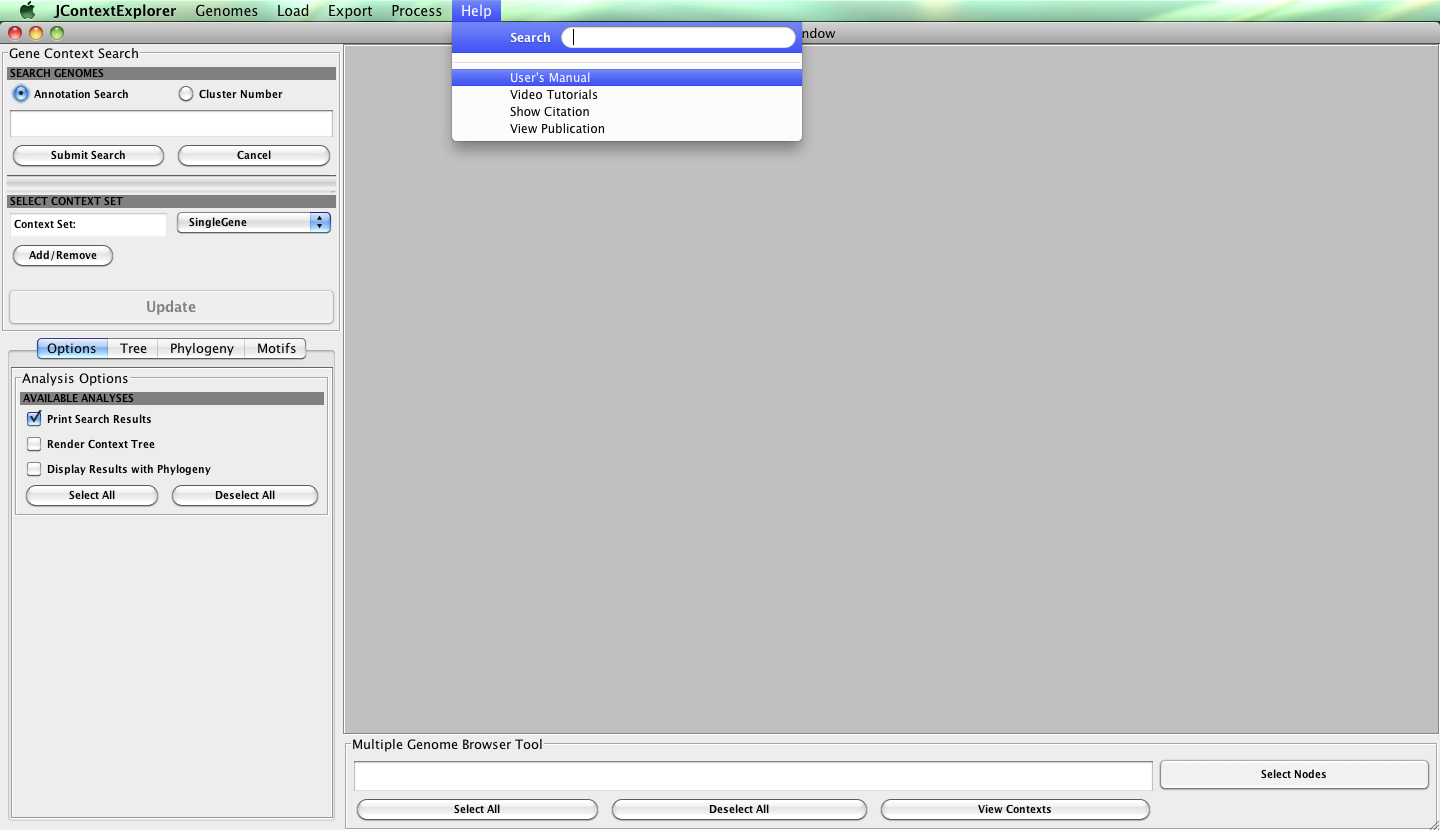


**(2) Querying the database - tutorial**

Type the locus tag of the specific gene of interest into the search bar located in the upper-left hand corner. Then, click the submit search button.

As an example, let’s type in VNG0410G:


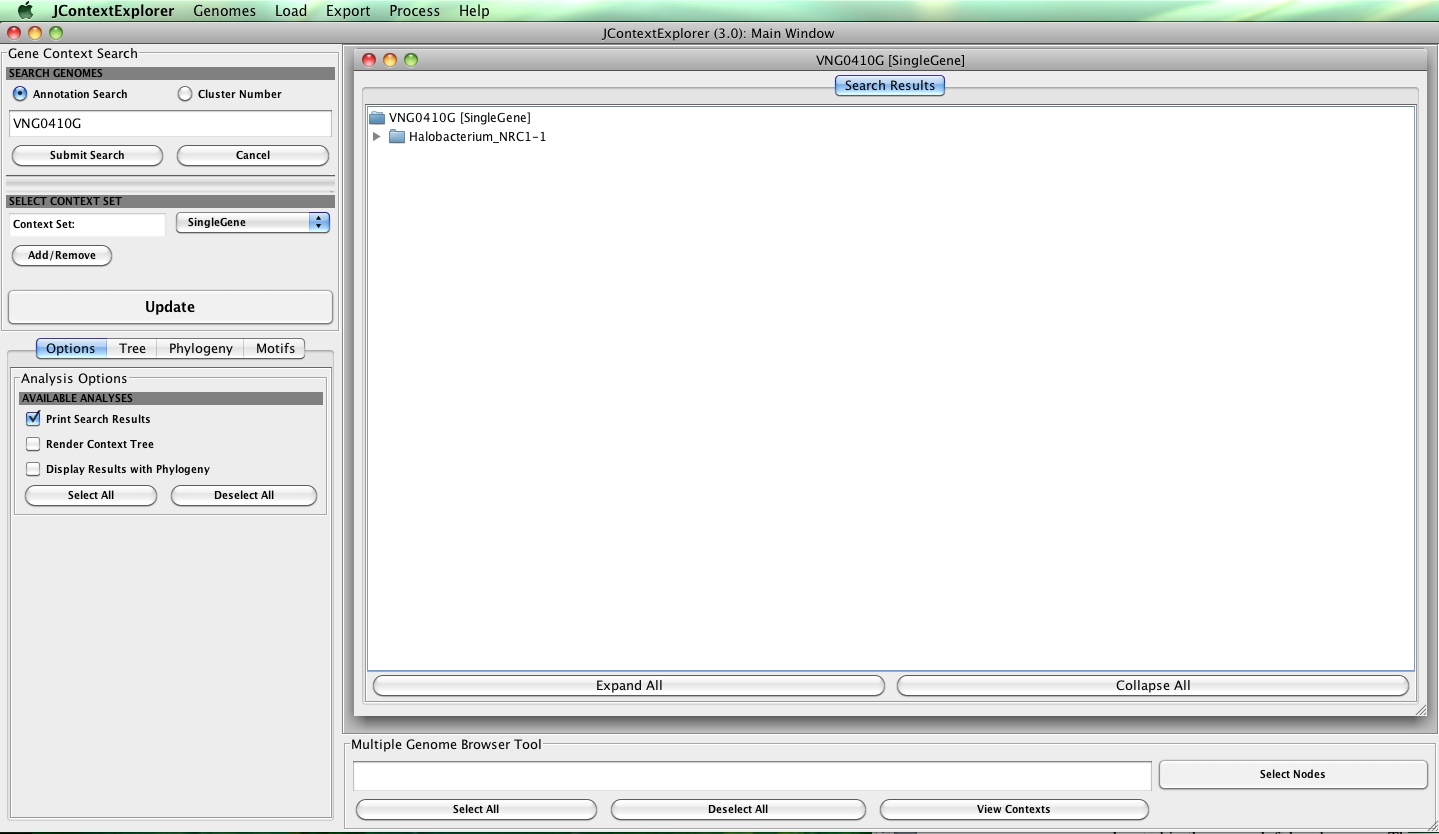


After clicking “Submit Search”, the following internal window appears:


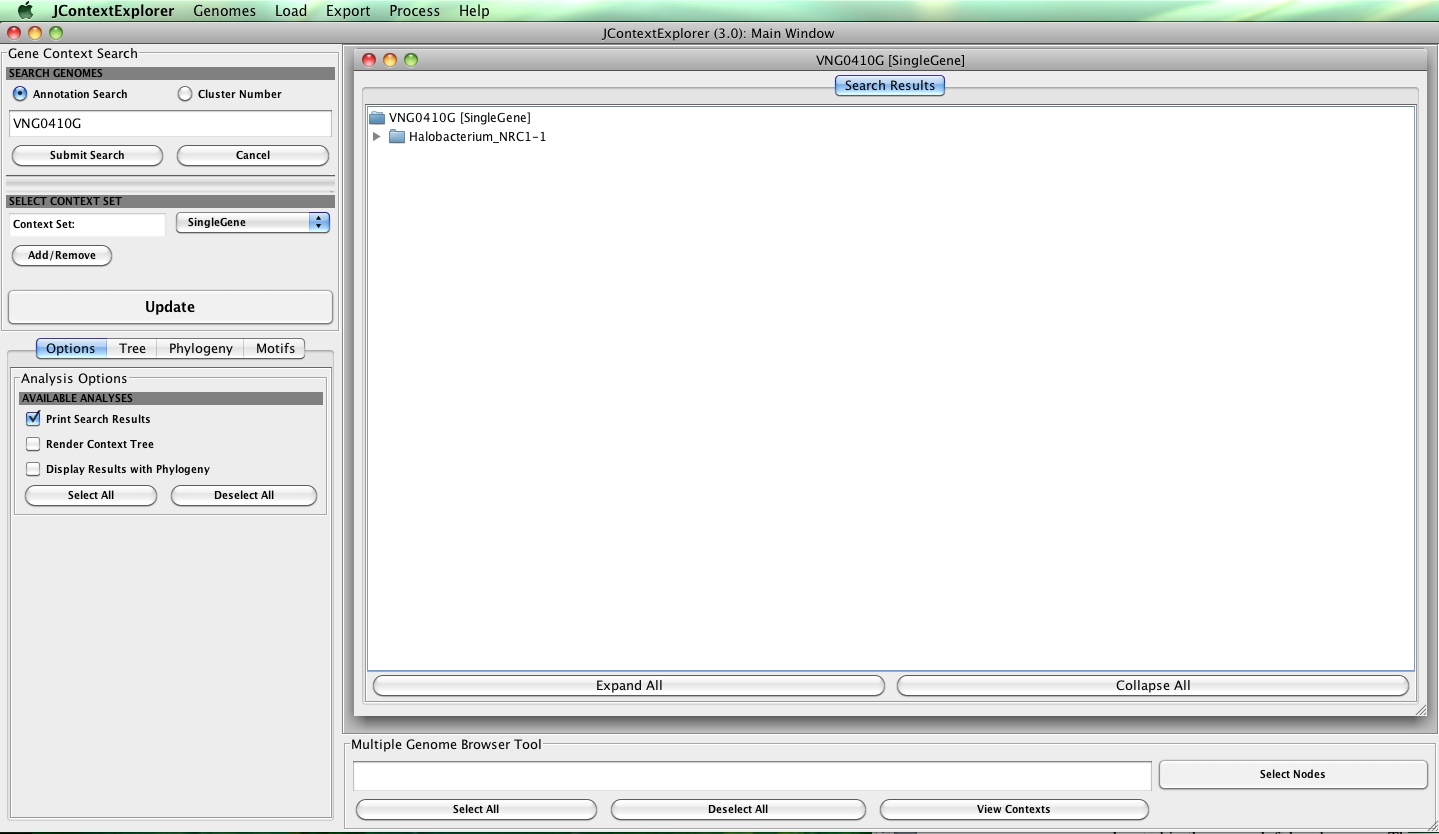


Clicking on the triangle reveals the information about this gene, which is located in the organism Halobacterium_NRC1:


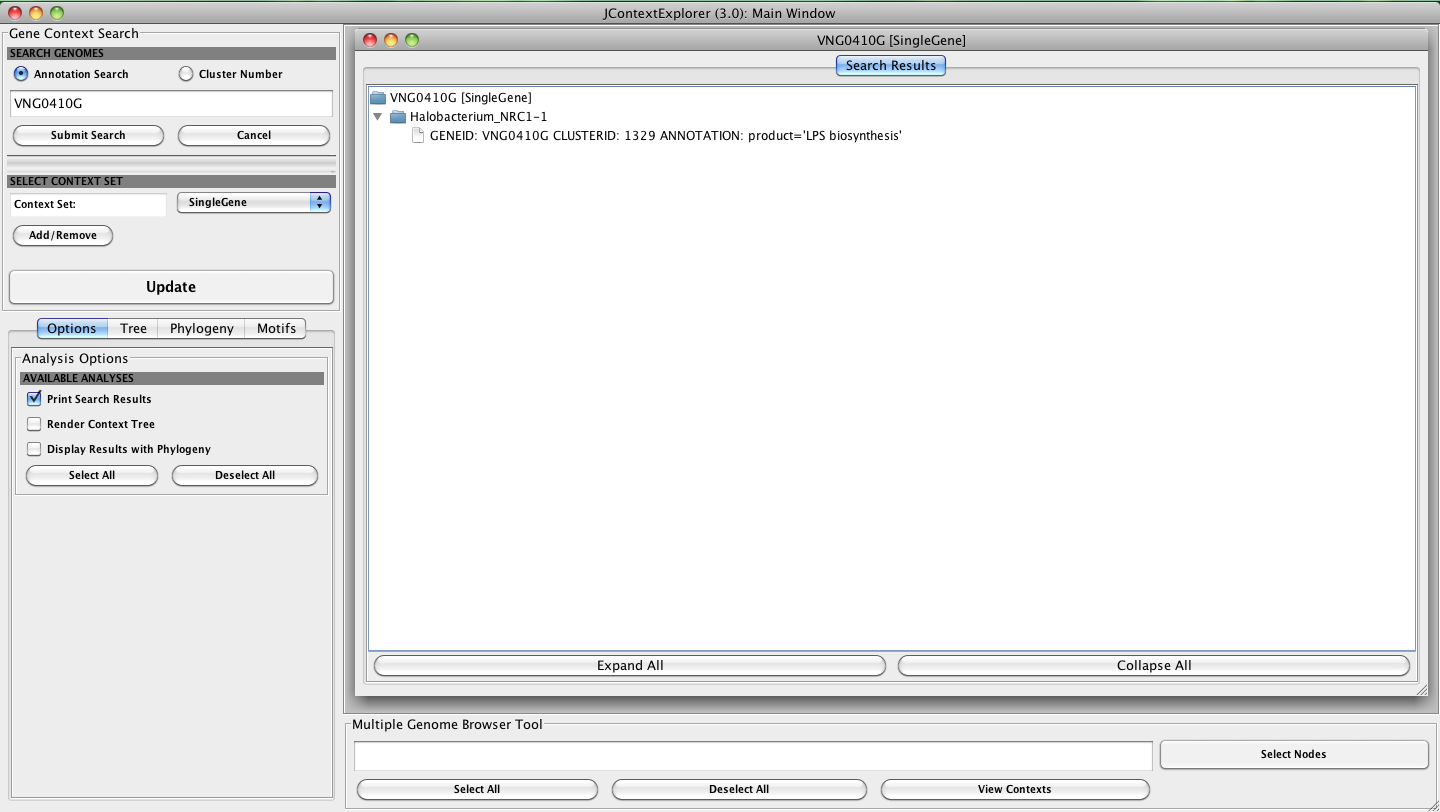


As you can see, this gene has the locus tag VNG0410G (our search), and is annotated as product=”LPS biosynthesis”, and is a member of the homology group with cluster ID 1329.

In the search window, type LPS biosynthesis, and click submit:


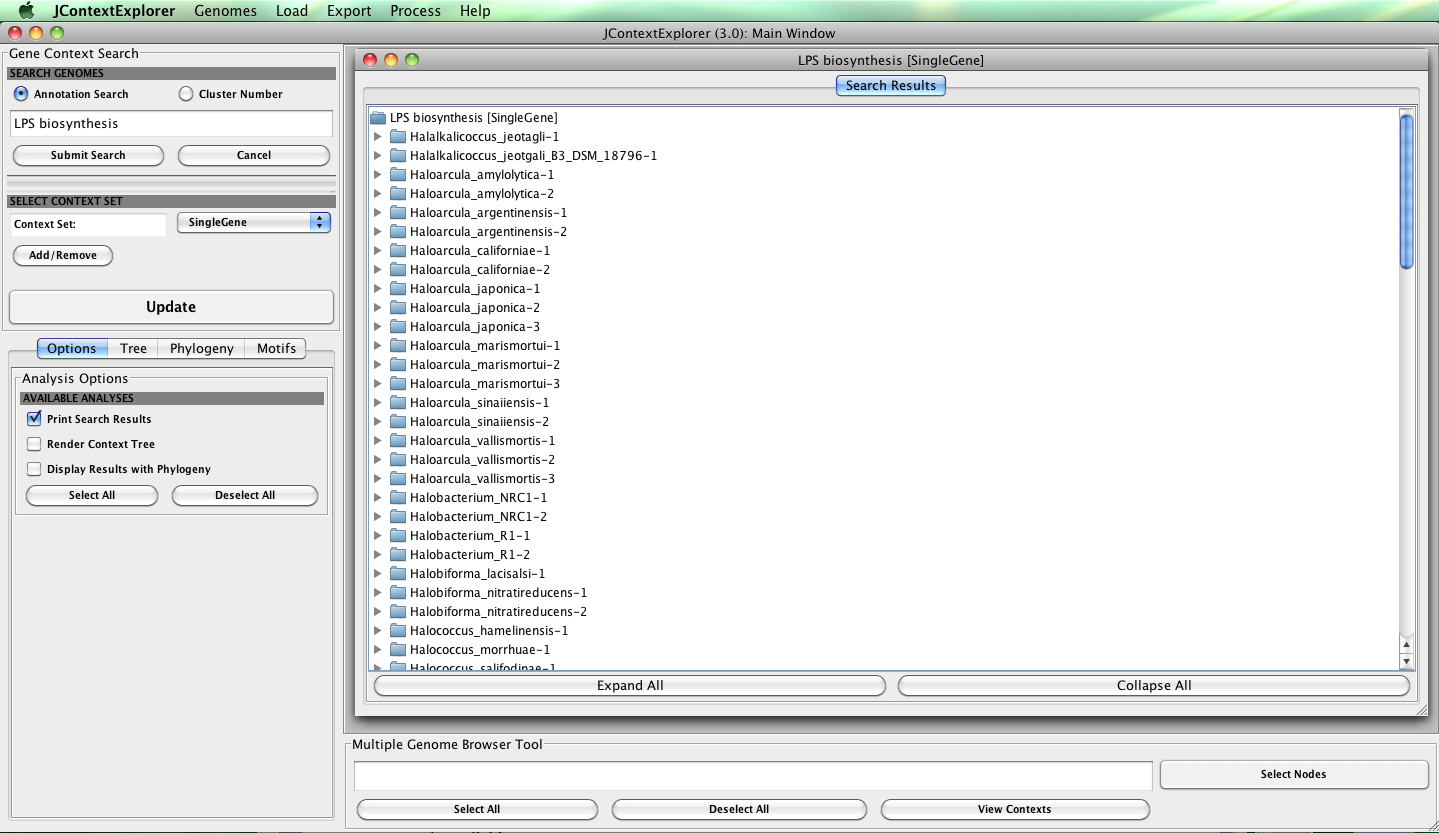


This will return a list of all genes in the haloarchaea that have annotations containing the text fragment LPS biosynthesis. Shown is the results frame:


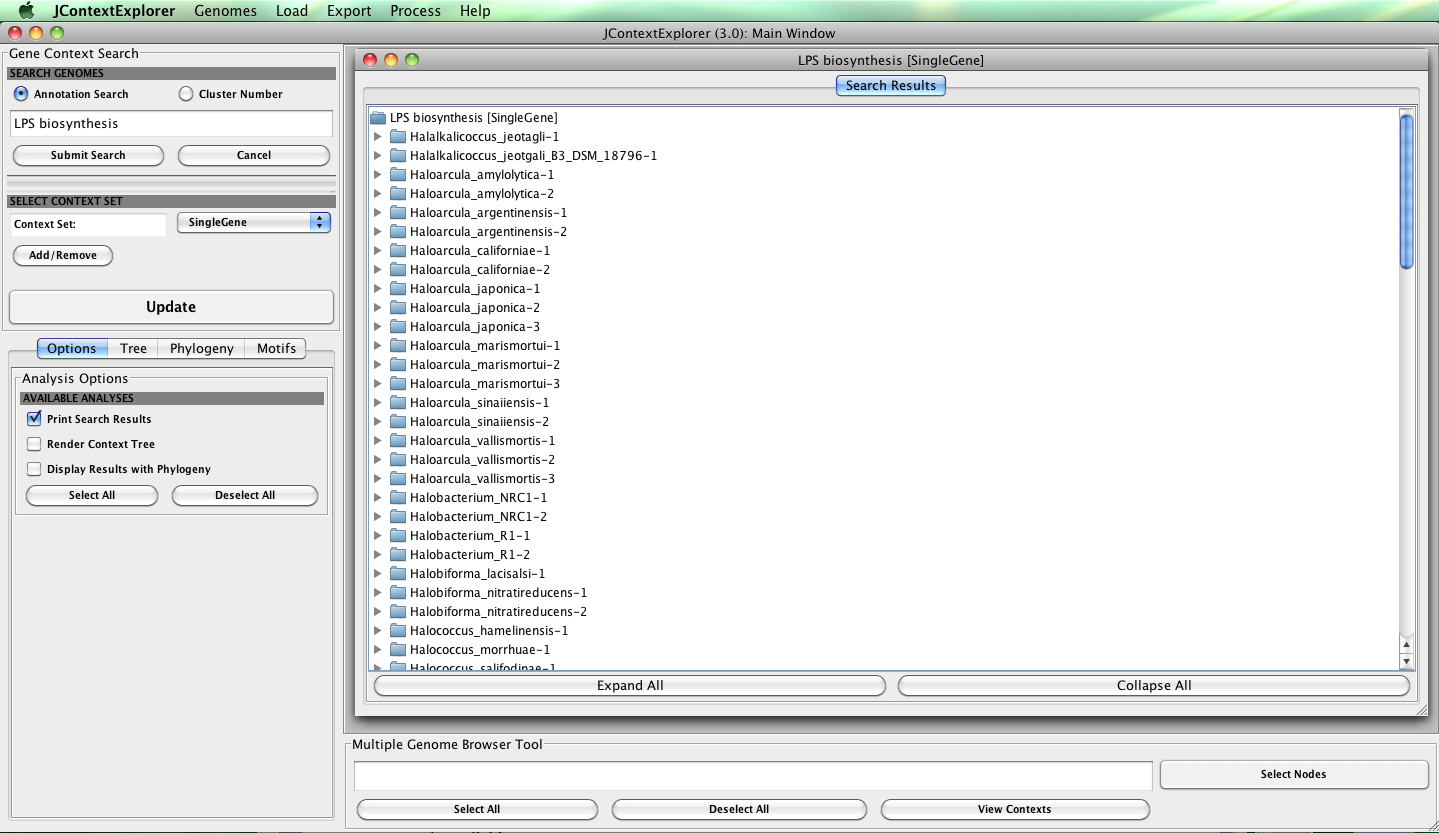


We discovered earlier that the gene VNG0410G is in the cluster group with cluster ID 1329. We can enumerate the protein instances of a given homology cluster by switching to a cluster number search. In the main frame, switch from the “Annotation Search” to “Cluster Number” radio button, type 1329, and click submit search.


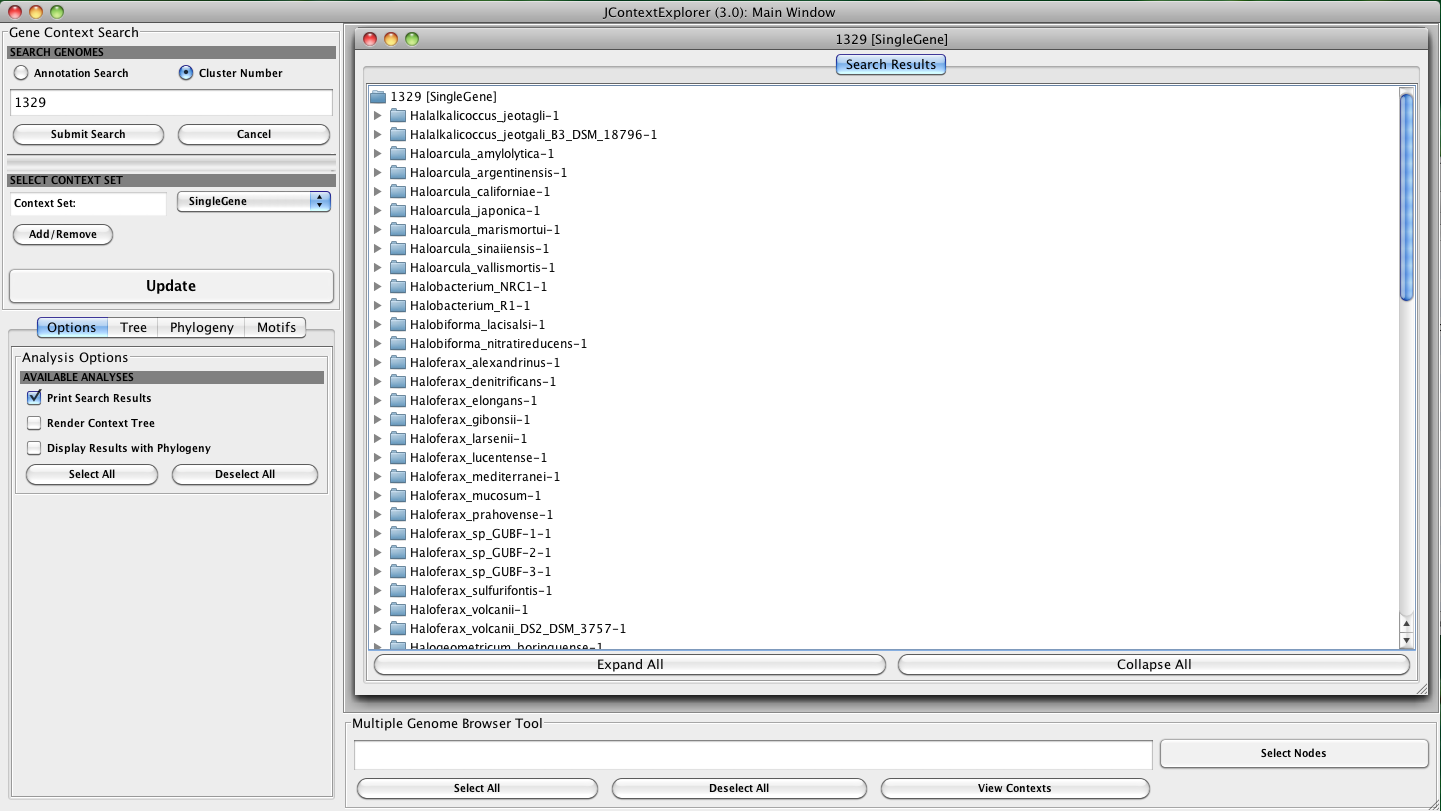


The following results window will appear:


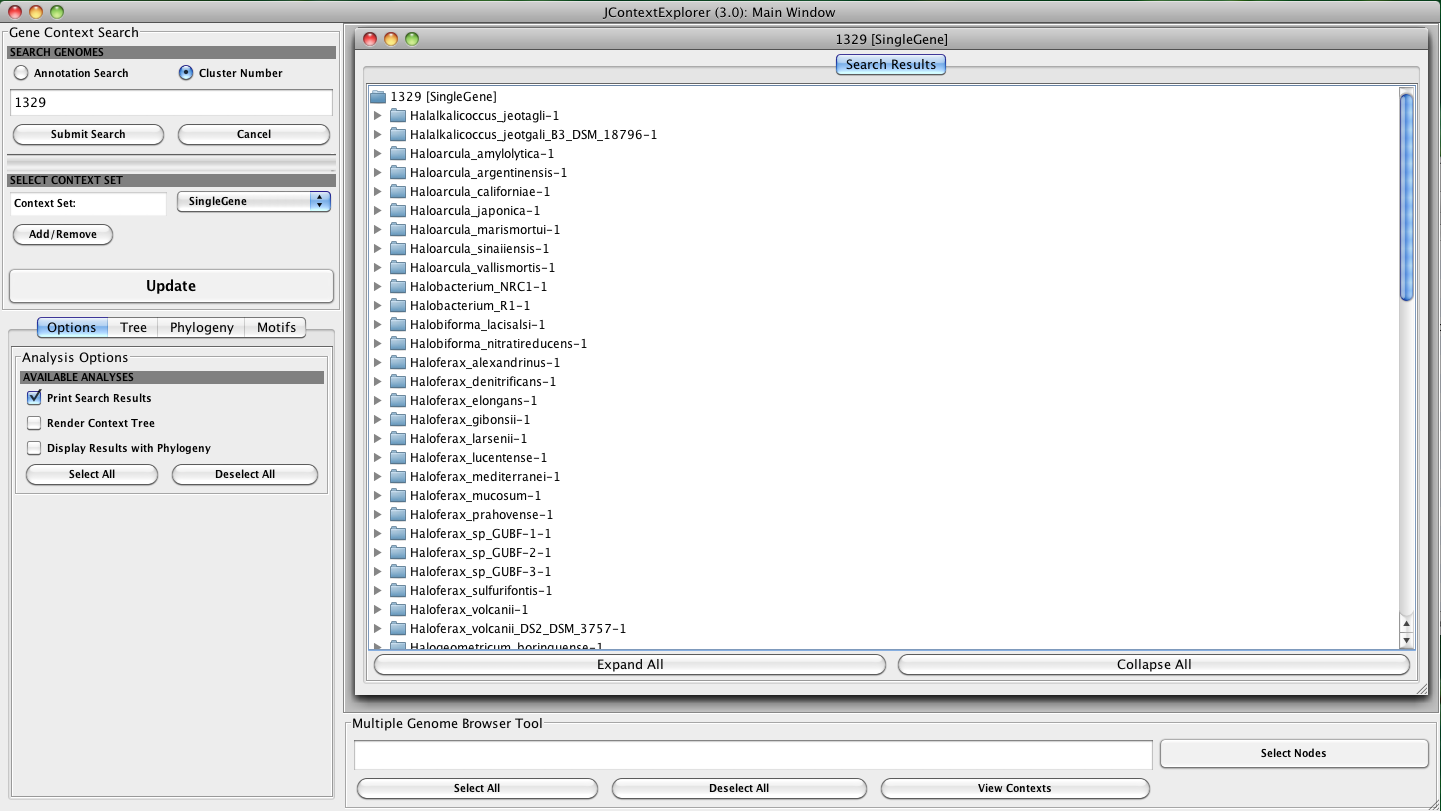


To visualize the occurrence of these genes in these organisms, select one or more folders in the internal frame, and click the “View Contexts” button located in the bottom part of the main frame, under the heading “Multiple Genome Browser Tool”.

In this example, let’s click the “Select All” button which is located in the bottom part of the main frame, under the Multiple Genome Browser tool. After doing so, all folders should appear selected:


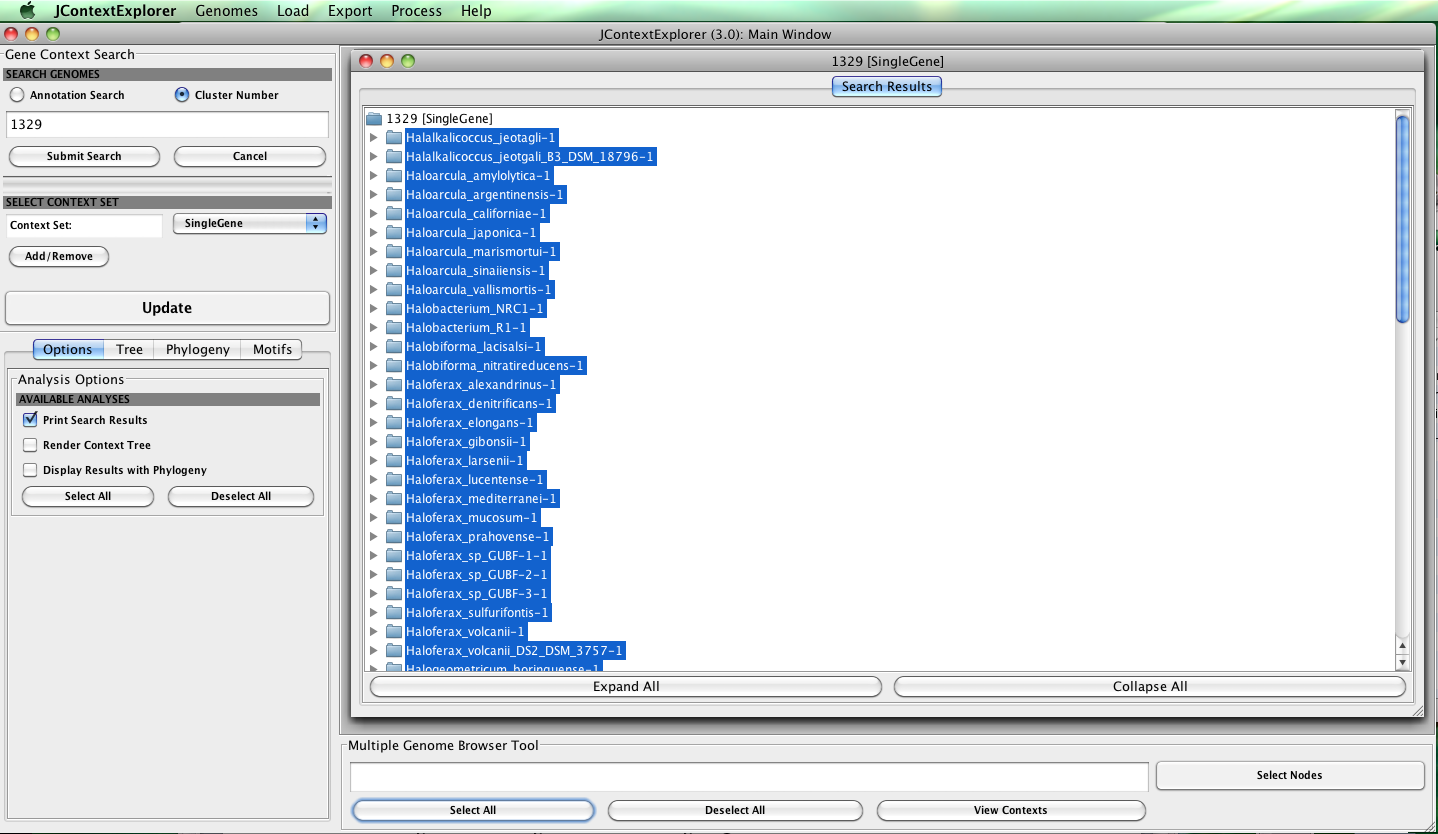


Now, click the “View Contexts” button (selected in blue in image below):


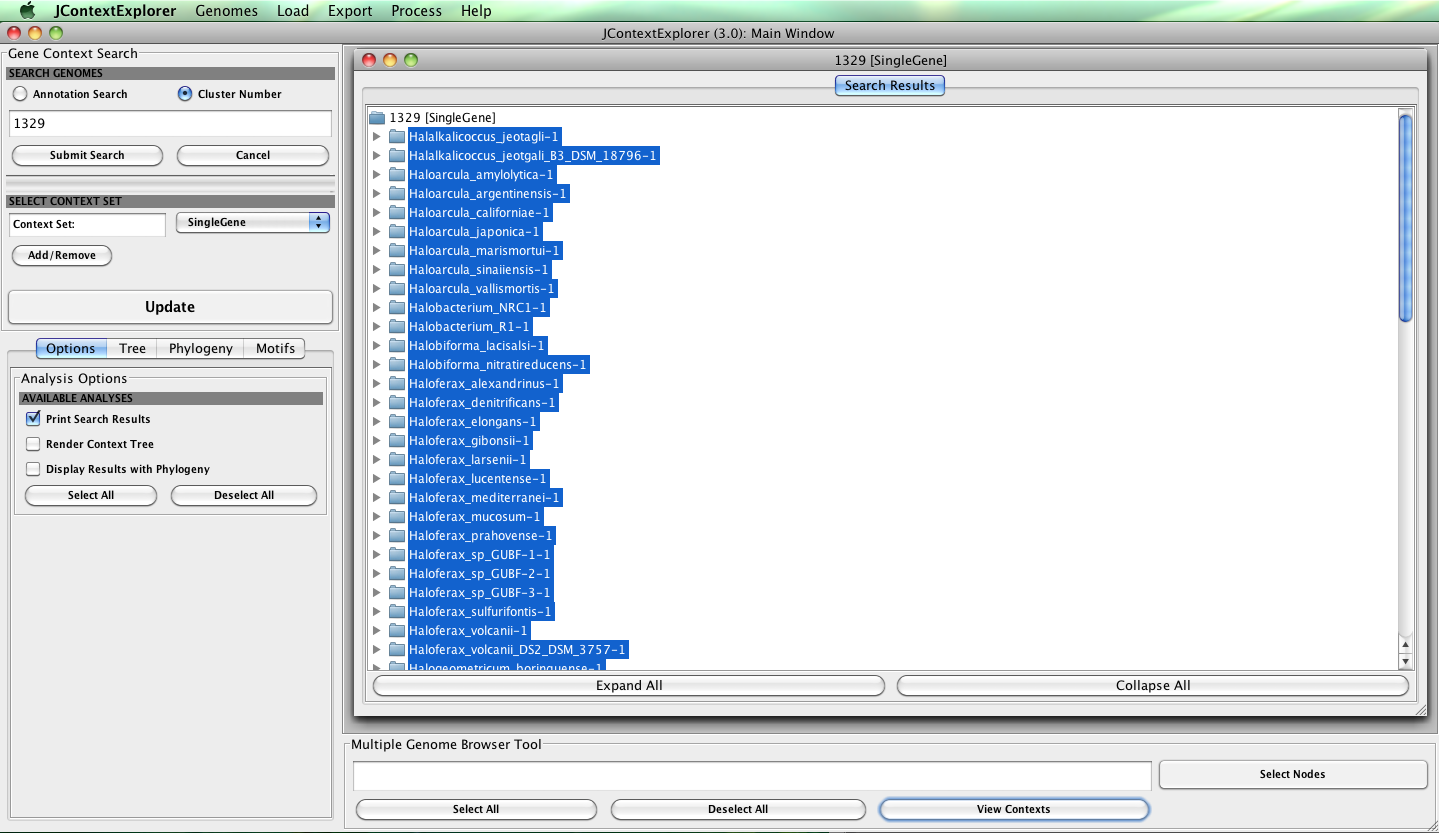


The following window will appear:


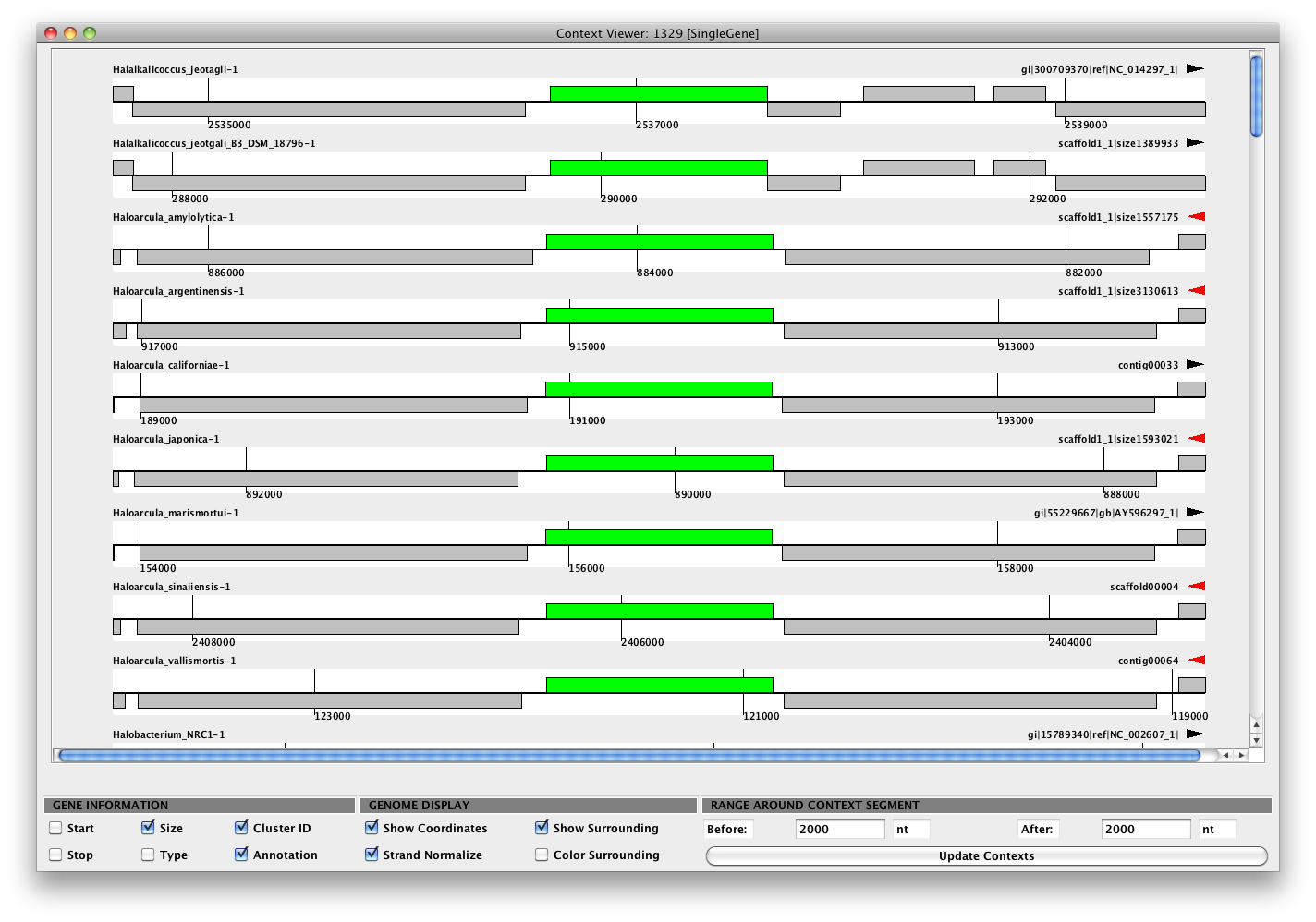


For a more detailed description of how to browse contexts, please refer to the user’s manual + video tutorials.

Close this window.

Now, we will extract the protein sequences for all genes in this cluster. In the main window, all clusters should be selected. Right click on one of the folders. A tiny pop-up window should appear with a number of sequence export options. Select the second option, “Export Protein Sequences”.


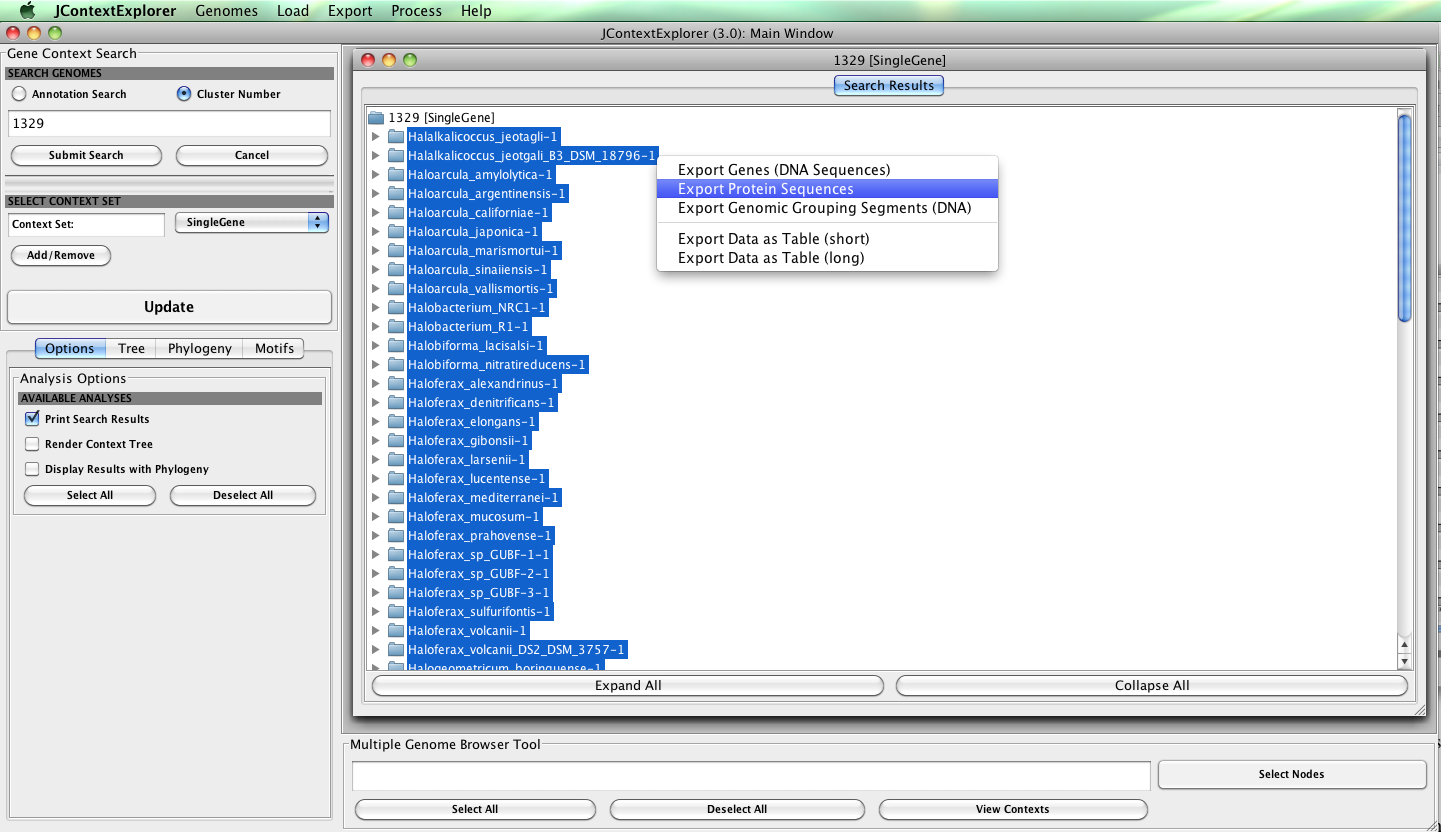


A window will pop up asking you to select a location on your computer to save the exported sequences. Select an appropriate location, and choose an appropriate file name.


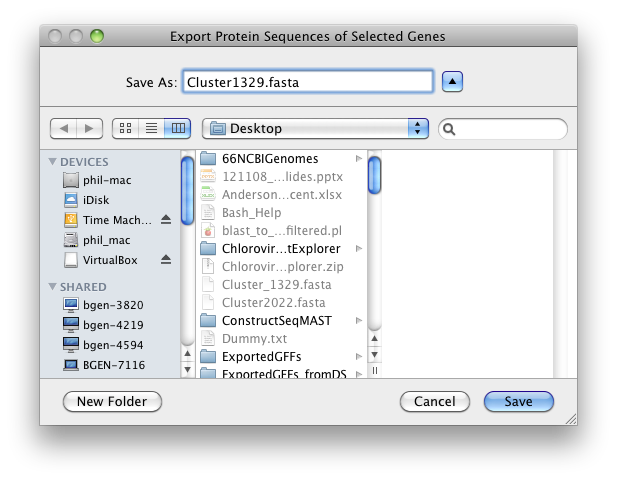


Please wait while the data is downloaded onto your computer. You can follow the progress in the blue progress bar in the main frame.

Finally, the file should appear in your saved location. Opening the file in a text editor reveals a .fasta-formatted file containing all protein sequences in cluster 1329.


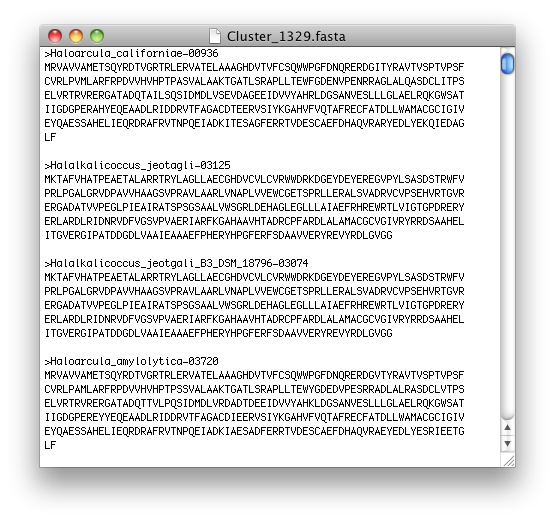


The headers in this file are the gene IDs. Initially, we searched for VNG0410G. We can find the protein sequence for this gene within the file, among other protein sequences:


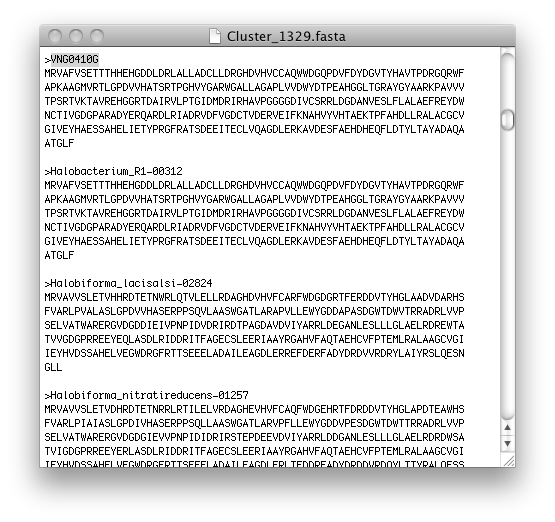


This completes the basic tutorial. For more detailed tutorials and instructions, please refer to the user’s manual + video tutorials.

1. **Accessing haloarchaeal genomic data through SQL database.**

The haloarchaeal genomic data is available as a navigable SQL database, however is not currently being hosted publically because of its large size. A copy of the .sql database file is available upon request.

**D. Other haloarchaeal genome sequencing projects:**

a. Independently sequenced (species included in this project):

i. *Halalkalicoccus jeotgali* (Roh, 2010)

ii. *Halobiforma lacisalsi* (Jiang, 2011)

iii. *Halococcus hamelinensis* (Burns, 2012)

iv. *Haloferax mediterranei* (Han, 2012)

v. *Halogeometricum borinquense* (Malfatti, 2009)

vi. *Natrialba magadii* (Siddaramappa, 2012)

b. Sequencing data available (species not included in this project):

i. *Haladaptatus paucihalophilus* DX253

ii. *Halarchaeum acidiphilum*

iii. *Halogranum salarium* B-1 (Kim, 2012)

iv. *Halomicrobium katesii*

v. halophilic archaeon True-ADL

vi. *Haloplanus natans*

vii. *Halorhabdus tiamatea* (Antunes, 2011)

viii. *Halorubrum ezzemoulense*

ix. *Halovivax ruber*

x. *Natronococcus occultus*

xi. *Natronomonas moolapensis* (Dyall-Smith, 2013)

c. No sequencing data available:

i. *Haloarcula sp.* AS7094

ii. *Halobaculum gomorrense*

iii. *Halococcus sp.* 197A

iv. *Halostagnicola larsenii*

**E. Species without annotated photolyases.**

a. *Haloterrigena turkmenica*

b. *Haloterrigena salina*

c. *Halovivax asiaticus*

d. *Natrialba hulunbeirensis*

e. *Natrialba asiatica*

f. *Natrialba taiwanensis*

g. *Natrialba aegyptia*

**F. Species not included in GC bias analysis.**

a. *Haloarcula californiae* (too many contigs)

b. *Haloarcula vallismortis* (too many contigs)

c. *Halobiforma nitratireducens* (too many contigs)

d. *Halococcus morrhuae* (too many contigs)

e. *Halococcus salifodinae* (too many contigs)

f. *Haloferax mucosum* (too many contigs)

g. *Haloferax sulfurifontis* (too many contigs)

h. *Halomicrobium mukohataei* (no changepoints)

i. *Halorubrum californiensis* (too many contigs)

j. *Halovivax asiaticus* (no changepoints)
